# HMMR as a robust prognostic biomarker correlates with immune infiltration and cell cycle pathways in oral squamous cell carcinoma: a multi-cohort bioinformatics analysis based on TCGA and GEO databases

**DOI:** 10.3389/fgene.2026.1764943

**Published:** 2026-06-05

**Authors:** Lingling Liu, Shiyan Chen, Hongxun Gong, Xingfeng Qi, Maoxin Wang

**Affiliations:** 1 Fujian Key Laboratory of Oral Diseases & Fujian Provincial Engineering Research Center of Oral Biomaterial & Stomatological Key Lab of Fujian College and University, School and Hospital of Stomatology, Fujian Medical University, Fuzhou, China; 2 Department of Otolaryngology, Fujian Medical University Union Hospital, Fuzhou, China; 3 Department of Otorhinolaryngology Head and Neck, Fuzong Clinical College of Fujian Medical University, The 900th Hospital of Joint Logistic Support Force of PLA, Fuzhou, China; 4 Department of Pathology, Fuzong Clinical Medical College of Fujian Medical University/The 900th Hospital of Joint Logistic Support Force, PLA, Fuzhou, China

**Keywords:** bioinformatics, HMMR, oral squamous cell carcinoma, prognostic value, TCGA

## Abstract

**Introduction:**

Oral squamous cell carcinoma (OSCC), a prevalent head and neck malignancy with complex molecular pathogenesis and poor prognosis, remains a critical clinical challenge. This study aimed to elucidate the role of hyaluronic acid mediated motility receptor (HMMR) in OSCC progression and evaluate its potential as a diagnostic and prognostic biomarker.

**Methods:**

Transcriptomic data was integrated from the TCGA OSCC cohort and two independent GEO cohorts (GSE37991, GSE41613). Differential expression analysis was performed using DESeq2. Common differentially expressed genes (DEGs) were identified via Venn diagram analysis, followed by functional enrichment analysis using the STRING database. Univariate and multivariate Cox regression analyses were conducted with the survival package to identify prognostic markers, and the predictive performance was evaluated by receiver operating characteristic (ROC) analysis. Immune infiltration analysis, pathway enrichment analysis, and protein–protein interaction (PPI) network analysis via GeneMANIA were further applied to explore biological functions.

**Results:**

A total of 2,477 upregulated and 2,214 downregulated differentially expressed genes (DEGs) were identified, with 245 common DEGs pinpointed across datasets. Cluster1 genes were significantly associated with OSCC pathogenesis in functional enrichment. CCNA1, HMMR, and RAG1 were identified as potential prognostic markers, with HMMR demonstrating the strongest predictive value (AUC = 0.919 in ROC analysis). HMMR was significantly overexpressed in OSCC tissues compared to normal controls (P < 0.001), and its high expression correlated with advanced T stage, N stage, pathological grade, shorter disease-specific survival, and disease-free survival. Multivariate analysis confirmed HMMR as an independent prognostic factor for overall survival. Immune infiltration analysis revealed that HMMR expression was positively correlated with 11 immune cell subsets, particularly Th2 cells (r = 0.465) and T helper cells (r = 0.356), suggesting a role in modulating the tumor immune microenvironment. Pathway enrichment linked HMMR to tumor proliferation, hypoxia response, DNA damage repair, and G2/M cell cycle checkpoints. The PPI network analysis identified HMMR-associated complexes involving cell cycle regulators (e.g., CDK1, CCNB1, AURKA), implicating roles in cell cycle regulation, DNA repair, and mitotic progression.

**Discussion:**

Collectively, this study establishes HMMR as a robust prognostic biomarker for OSCC, tightly associated with malignant progression, immune cell infiltration, and key oncogenic pathways. HMMR and its interacting network represent promising targets for OSCC precision medicine, offering new insights into diagnostic strategies and therapeutic development.

## 1 Introduction

Oral Squamous Cell Carcinoma (OSCC) ranks amongst the most prevalent neoplasms globally, constituting in excess of 90% of all oral neoplasms (Pekarek, 2023). The frequency and fatality rates of OSCC exhibited substantial variation worldwide, primarily attributable to lifestyle practices and environmental constituents (Simba et al., 2023). Tobacco and alcohol consumption emerged as the principal risk determinants for OSCC, with their combined effect considerably amplifying the OSCC risk (Yang et al., 2023). Moreover, infection with Human Papillomavirus (HPV), particularly HPV16, was deemed a significant risk determinant for OSCC (Saleh et al., 2023). In certain regions, such as Hu’nan Province of China, dietary practices (e.g., betel nut mastication) also posed substantial risk factors for OSCC (Wang et al., 2023). Furthermore, age, gender, socioeconomic standing, and genetic susceptibility were also correlated with morbidity and mortality in OSCC. The primary therapeutic strategies for OSCC encompassed surgical excision, radiotherapy, and chemotherapy (Kende et al., 2024). Despite these conventional therapies enhancing patient survival rates somewhat, their therapeutic efficacy remained unsatisfactory due to issues such as severe side effects, restricted treatment impact, and high recurrence frequency.

In recent years, the advent of targeted therapies and immunotherapies had ushered in renewed optimism for OSCC treatment (Sharon et al., 2023). Consequently, a comprehensive comprehension of OSCC’s pathological mechanisms is pivotal to augmenting the prevention, diagnosis, and treatment of OSCC.

The Hyaluronan-Mediated Motility Receptor (HMMR) was a cell surface receptor that modulates cell migration and proliferation via hyaluronic acid (HA) binding (Carvalho et al., 2023). Recent studies have discovered that HMMR was upregulated in a multitude of neoplasms, exhibiting a close association with tumor onset, progression, metastasis, and prognosis (Hinneh et al., 2023; Tarullo et al., 2023; Rizzardi et al., 2014). Contemporary research has elucidated that HMMR was instrumental in the genesis and progression of an array of neoplasms, encompassing breast, colon, lung, gastric, and nasopharyngeal malignancies (Purnell, 2017; Tang et al., 2021; Gao et al., 2019; Zhang et al., 2019). Nevertheless, the function of HMMR in OSCC remained enigmatic.

In this article, we undertook a comprehensive exploration of the expression and role of HMMR in OSCC, with the objective of unearthing the function of HMMR in the inception and evolution of OSCC, thereby offering novel targets for the precocious diagnosis and intervention of OSCC.

## 2 Methods

### 2.1 Presentation of data and procurement of clinical data

RNA-seq transcriptome data and corresponding clinical information for Head and Neck Squamous Cell Carcinoma (HNSC) were downloaded from The Cancer Genome Atlas (TCGA) database, retaining samples corresponding to oral cancer sites (alveolar ridge, tongue root, buccal mucosa, oral floor, hard palate, oral cavity, oral tongue) within the clinical information (inclusive of 32 adjacent normal tissues and 362 neoplastic tissues).

Identification of Genes Correlated with Differential Expression and Prognosis

Disease status (neoplastic or normal) was employed as a variable to screen DEGs in OSCC using the DEseq2 package of R software. The filtering criteria were set as follows: baseMean>100, |log2FoldChange|>1, padj<0.05, and only protein-coding genes were retained. The threshold of baseMean >100 was applied to filter out low-abundance genes with high technical noise, which effectively reduces false positive results in differential expression analysis. The |log2FoldChange|>1 threshold (corresponding to a 2-fold change in expression) is a widely accepted standard in cancer transcriptomics studies to identify biologically meaningful expression alterations. A total of 362 OSCC tumor tissues and 32 adjacent normal oral tissues from the TCGA-OSCC dataset were included in this analysis.

### 2.2 Construction of protein-protein interaction network and module examination

The STRING online repository (http://string-db.org) was employed for the prediction of PPI networks, and the scrutiny of functional interplayed between proteins can furnish insights into the mechanisms underlying disease inception or progression. The STRING repository was utilized to investigate the protein-protein interactions of genes correlated with differential expression and prognosis in OSCC.

Functional enrichment analysis was performed on the gene set using the clusterProfiler R package (v4.8.3). The threshold was set as adjusted P-value (FDR) < 0.05 to screen for significantly enriched functional terms. An enrichment bubble plot was generated using the ggplot2 package, with Signal value on the x-axis, FDR value for bubble color, gene count for bubble size, and terms clustered by functional similarity.

### 2.3 Clinicopathological characteristics, prognosis, model formulation and evaluation

The Kaplan-Meier methodology was employed to evaluate the differences in overall survival (OS), progression-free interval (PFI), and disease-specific survival (DSS) between HMMR high and low expression cohorts. In the TCGA cohort, patients were divided into high and low HMMR expression groups using the median expression value as the cutoff. In the GSE41613 cohort, the optimal cutoff value (8.055) determined by maximally selected rank statistics was used for dichotomization, which is a standard and unbiased approach for dichotomization in prognostic biomarker studies.

Univariate and multivariate Cox regression analyses were performed using the R software survival package to identify independent prognostic factors for OS. The proportional hazards assumption of the Cox regression models was verified using the Schoenfeld residual test, and all variables included in the final model satisfied the proportional hazards assumption (all P > 0.05). Hazard ratios (HRs) and 95% confidence intervals (CIs) were calculated for each prognostic factor.

### 2.4 Analysis of immune cell infiltration

The relative tumor infiltration degrees of 24 immune cell types were quantified by the single-sample gene set enrichment assay (ssGSEA) in the GSVA package of R software, and the correlation between HMMR and immune cell infiltration and HMMR high and low cohorts was explored via Wilcoxon rank sum test and Spearman correlation and visualized analysis. For single-cell sequencing analysis, the scRNA-seq dataset GSE103322 (containing 5902 cells from 18 OSCC patients) was downloaded from the Gene Expression Omnibus (GEO) database in.h5 format. The data preprocessing and analysis were performed using the Seurat and MAESTRO packages in R software following the standard workflow. Firstly, cells with <200 or >6,000 detected genes, and cells with >10% mitochondrial gene expression were filtered out to remove low-quality cells and doublets. Then,t he data were normalized using the LogNormalize method, and the top 2000 highly variable genes were identified for downstream analysis. Thirdly, Principal component analysis (PCA) was performed, and the first 30 principal components were used for clustering with a resolution of 0.5. The t-distributed stochastic neighbor embedding (t-SNE) method was used for visualization of cell clusters. Lastly, cell clusters were annotated using the cell type annotations provided by the TISCH database, and the annotations were manually verified using canonical cell type markers. For protein expressions in clinical samples, The Human Protein Atlas database was employed to verify the expression of HMMR in OSCC.

### 2.5 Protein–protein interaction networks (PPI) construction

To dissect the interaction landscape of HMMR in OSCC, we first targeted genes that potentially interact with HMMR. These candidate interacting genes were retrieved from the GeneMANIA database (https://genemania.org/). Leveraging this platform, we harnessed its integrated algorithm, which aggregated multi-omics data (including physical interactions, co-expression patterns, and genetic interactions) to screen and curate HMMR-associated interactors systematically. Subsequently, these retrieved genes, together with HMMR, were utilized to construct a comprehensive PPI network. This network construction step aimed to map out the intricate molecular connections centered on HMMR, enabling us to visualize and analyze how HMMR engages with other proteins at a system-level in the context of OSCC. By integrating diverse interaction evidence from the database, the resulting PPI network served as a foundational framework for further deciphering the collaborative functional modules and regulatory mechanisms involving HMMR in OSCC biology.

### 2.6 Statistical analysis

The Mann-Whitney U test was employed to assess the disparities in ssGSEA scores of immune cells or pathways amid risk groups, with P-values were adjusted using the Benjamini–Hochberg (BH) method. Kaplan-Meier scrutiny and log-rank test were utilized to juxtapose the OS of each faction. Univariate and multivariate Cox regression analyses pinpointed independent prognostic determinants for OS. P < 0.05 is deemed statistically significant.

## 3 Results

### 3.1 Screening of DEGs through differential expression analysis and prognostic evaluation

**FIGURE 1:**
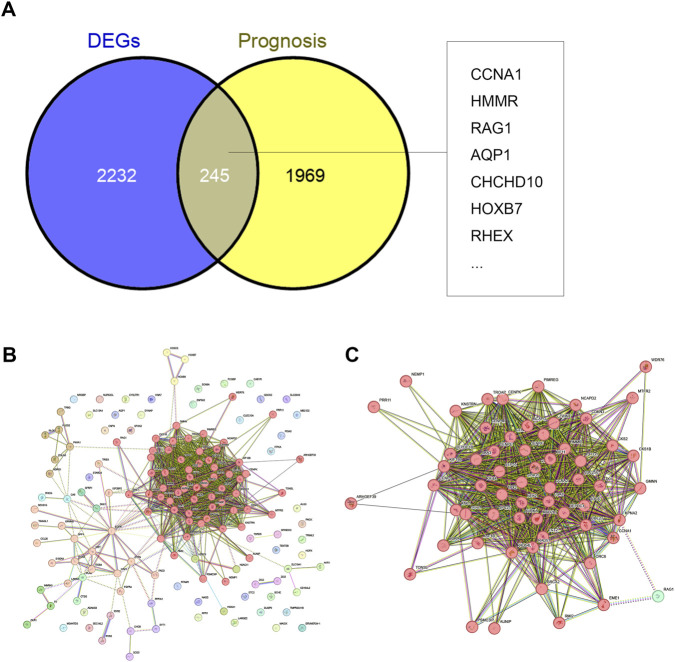
Differential Expression and Prognostic-Related Gene Selection in OSCC. **(A)** A bar chart illustrates the number of differentially expressed genes (DEGs) identified using DEseq2 (2,477 genes) and survival analysis (2,214 genes) in TCGA OSCC data. A Venn diagram depicts the overlap between these two gene sets, revealing 245 common genes. **(B,C)** Functional enrichment analysis of the 245 selected genes was conducted using the String website. Cluster one is particularly noteworthy for its close association with OSCC, as evident from the heatmap and functional annotation.

Employing the DEseq2 and survival packages, a comprehensive analysis of the TCGA OSCC dataset was conducted, resulting in the identification of 2,477 and 2,214 differentially expressed genes (DEGs), respectively (Supplementary Table S1). Subsequently, the intersection of these two gene sets was determined through a Venn diagram analysis, yielding a total of 245 common DEGs (Figure 1A). Functional enrichment analysis of the 245 DEGs was conducted using the clusterProfiler package in R. GO enrichment analysis revealed that these genes were significantly enriched in biological processes (BP) such as organelle fission, nuclear division and nuclear chromosome segregation; cellular components (CC) such ascondensed chromosome, kinetochore and mitotic spindle; and molecular functions (MF) such as organic acid binding, carboxylic acid binding and cyclin-dependent protein serine/threonine kinase regulator activity (Supplementary Figure S1; Supplementary Table S3). KEGG pathway analysis showed that these genes were mainly involved in Cell cycle, Calcium signaling pathway and Progesterone-mediated oocyte maturation. These results were consistent with the known pathogenesis of OSCC and further support the critical role of cell cycle dysregulation in OSCC progression. These genes were then subjected to functional enrichment analysis on the String website (http://string-db.org), revealing that Cluster1 exhibited a stronger association with OSCC (Supplementary Table S2; Figures 1B,C).

Furthermore, univariate and multivariate COX regression analysis revealed that the DEGs CCNA1, HMMR, and RAG1 potentially exert a significant influence on the prognostic outcome of OSCC patients (Table 1).

**TABLE 1:** Genes significantly associated with prognostic outcomes in OSCC.

Characteristics	Total (N)	HR (95% CI) univariate analysis	P Value univariate analysis	HR (95% CI) multivariate analysis	P Value multivariate analysis
ARHGEF39	329	​	​	​	​
Low	165	Reference	​	Reference	​
High	164	1.566 (1.130–2.171)	0.007	1.280 (0.800–2.049)	0.304
ASF1B	329	​	​	​	​
Low	164	Reference	​	Reference	​
High	165	1.412 (1.022–1.952)	0.036	0.861 (0.496–1.495)	0.595
AUNIP	329	​	​	​	​
Low	164	Reference	​	Reference	​
High	165	1.425 (1.028–1.976)	0.034	0.889 (0.526–1.505)	0.662
AURKA	329	​	​	​	​
Low	165	Reference	​	Reference	​
High	164	1.437 (1.040–1.985)	0.028	0.848 (0.483–1.490)	0.567
BORA	329	​	​	​	​
Low	164	Reference	​	Reference	​
High	165	1.607 (1.161–2.225)	0.004	1.162 (0.690–1.955)	0.573
BRCA2	329	​	​	​	​
Low	164	Reference	​	Reference	​
High	165	1.409 (1.019–1.947)	0.038	0.842 (0.502–1.412)	0.514
CCNA1	329	​	​	​	​
Low	165	Reference	​	Reference	​
High	164	1.440 (1.043–1.988)	0.027	1.434 (1.006–2.044)	0.046
CCNA2	329	​	​	​	​
Low	164	Reference	​	Reference	​
High	165	1.579 (1.138–2.190)	0.006	0.951 (0.516–1.751)	0.872
CCNB2	329	​	​	​	​
Low	165	Reference	​	Reference	​
High	164	1.389 (1.007–1.917)	0.045	0.802 (0.446–1.440)	0.459
CDC25C	329	​	​	​	​
Low	165	Reference	​	Reference	​
High	164	1.429 (1.034–1.973)	0.030	0.803 (0.465–1.385)	0.430
CDC6	329	​	​	​	​
Low	165	Reference	​	Reference	​
High	164	1.546 (1.118–2.138)	0.008	1.543 (0.855–2.787)	0.150
CDK1	329	​	​	​	​
Low	164	Reference	​	Reference	​
High	165	1.483 (1.069–2.056)	0.018	0.844 (0.423–1.684)	0.631
CDKN3	329	​	​	​	​
Low	165	Reference	​	Reference	​
High	164	1.451 (1.050–2.006)	0.024	0.974 (0.576–1.648)	0.923
CENPA	329	​	​	​	​
Low	165	Reference	​	Reference	​
High	164	1.726 (1.245–2.392)	0.001	1.359 (0.745–2.476)	0.317
CENPE	329	​	​	​	​
Low	164	Reference	​	Reference	​
High	165	1.686 (1.217–2.336)	0.002	1.508 (0.814–2.792)	0.192
CENPF	329	​	​	​	​
Low	164	Reference	​	Reference	​
High	165	1.521 (1.098–2.108)	0.012	1.352 (0.715–2.556)	0.354
CENPI	329	​	​	​	​
Low	164	Reference	​	Reference	​
High	165	1.439 (1.041–1.990)	0.028	0.699 (0.366–1.337)	0.279
CENPK	329	​	​	​	​
Low	164	Reference	​	Reference	​
High	165	1.493 (1.078–2.068)	0.016	1.184 (0.667–2.101)	0.564
CENPM	329	​	​	​	​
Low	164	Reference	​	Reference	​
High	165	1.384 (1.001–1.914)	0.049	1.019 (0.617–1.682)	0.942
CEP55	329	​	​	​	​
Low	165	Reference	​	Reference	​
High	164	1.521 (1.097–2.109)	0.012	1.159 (0.610–2.202)	0.652
CKS1B	329	​	​	​	​
Low	164	Reference	​	Reference	​
High	165	1.485 (1.073–2.056)	0.017	1.123 (0.648–1.946)	0.680
CKS2	329	​	​	​	​
Low	165	Reference	​	Reference	​
High	164	1.588 (1.148–2.197)	0.005	1.069 (0.646–1.767)	0.795
DEPDC1	329	​	​	​	​
Low	164	Reference	​	Reference	​
High	165	1.565 (1.131–2.168)	0.007	0.808 (0.443–1.476)	0.488
DEPDC1B	329	​	​	​	​
Low	164	Reference	​	Reference	​
High	165	1.389 (1.007–1.918)	0.046	0.736 (0.443–1.222)	0.236
ECT2	329	​	​	​	​
Low	164	Reference	​	Reference	​
High	165	1.383 (1.001–1.912)	0.049	0.913 (0.547–1.522)	0.726
EME1	329	​	​	​	​
Low	165	Reference	​	Reference	​
High	164	1.450 (1.047–2.007)	0.025	0.816 (0.413–1.612)	0.558
GMNN	329	​	​	​	​
Low	165	Reference	​	Reference	​
High	164	1.439 (1.041–1.987)	0.027	1.160 (0.708–1.901)	0.555
HMMR	329	​	​	​	​
Low	165	Reference	​	Reference	​
High	164	1.813 (1.301–2.526)	<0.001	1.786 (1.046–3.050)	0.034
KIF11	329	​	​	​	​
Low	164	Reference	​	Reference	​
High	165	1.393 (1.007–1.926)	0.045	0.981 (0.525–1.830)	0.951
KIF18B	329	​	​	​	​
Low	164	Reference	​	Reference	​
High	165	1.388 (1.004–1.918)	0.047	0.754 (0.418–1.362)	0.349
KIF4A	329	​	​	​	​
Low	164	Reference	​	Reference	​
High	165	1.468 (1.062–2.029)	0.020	1.183 (0.607–2.304)	0.622
KNL1	329	​	​	​	​
Low	164	Reference	​	Reference	​
High	165	1.409 (1.020–1.947)	0.038	0.948 (0.540–1.664)	0.851
KNSTRN	329	​	​	​	​
Low	164	Reference	​	Reference	​
High	165	1.548 (1.119–2.141)	0.008	1.282 (0.690–2.383)	0.431
KPNA2	329	​	​	​	​
Low	165	Reference	​	Reference	​
High	164	1.596 (1.154–2.209)	0.005	1.196 (0.686–2.085)	0.529
MAD2L1	329	​	​	​	​
Low	164	Reference	​	Reference	​
High	165	1.631 (1.177–2.261)	0.003	1.206 (0.674–2.157)	0.527
MTFR2	329	​	​	​	​
Low	165	Reference	​	Reference	​
High	164	1.417 (1.023–1.964)	0.036	0.968 (0.538–1.741)	0.912
NCAPD2	329	​	​	​	​
Low	164	Reference	​	Reference	​
High	165	1.462 (1.052–2.030)	0.024	0.809 (0.458–1.427)	0.464
NCAPG	329	​	​	​	​
Low	164	Reference	​	Reference	​
High	165	1.482 (1.071–2.051)	0.018	1.182 (0.756–1.849)	0.463
NEK2	329	​	​	​	​
Low	165	Reference	​	Reference	​
High	164	1.554 (1.118–2.159)	0.009	0.938 (0.504–1.743)	0.839
NEMP1	329	​	​	​	​
Low	164	Reference	​	Reference	​
High	165	1.551 (1.115–2.159)	0.009	1.232 (0.787–1.930)	0.362
NUF2	329	​	​	​	​
Low	165	Reference	​	Reference	​
High	164	1.575 (1.137–2.181)	0.006	1.193 (0.632–2.255)	0.586
ORC6	329	​	​	​	​
Low	165	Reference	​	Reference	​
High	164	1.406 (1.013–1.952)	0.041	0.760 (0.444–1.302)	0.318
PIMREG	329	​	​	​	​
Low	164	Reference	​	Reference	​
High	165	1.519 (1.097–2.105)	0.012	1.350 (0.856–2.128)	0.197
PRR11	329	​	​	​	​
Low	164	Reference	​	Reference	​
High	165	1.485 (1.074–2.054)	0.017	1.157 (0.704–1.899)	0.565
PSMC3IP	329	​	​	​	​
Low	164	Reference	​	Reference	​
High	165	1.470 (1.063–2.034)	0.020	1.105 (0.648–1.882)	0.715
PTTG1	329	​	​	​	​
Low	165	Reference	​	Reference	​
High	164	1.457 (1.055–2.012)	0.022	1.310 (0.861–1.992)	0.207
RAD51	329	​	​	​	​
Low	164	Reference	​	Reference	​
High	165	1.525 (1.103–2.109)	0.011	1.275 (0.768–2.117)	0.348
RAD54L	329	​	​	​	​
Low	165	Reference	​	Reference	​
High	164	1.382 (1.001–1.907)	0.049	0.846 (0.503–1.423)	0.529
RAG1	329	​	​	​	​
Low	164	Reference	​	Reference	​
High	165	1.588 (1.146–2.201)	0.006	1.699 (1.190–2.426)	0.004
RMI2	329	​	​	​	​
Low	165	Reference	​	Reference	​
High	164	1.472 (1.063–2.038)	0.020	1.217 (0.780–1.901)	0.387
SGO1	329	​	​	​	​
Low	164	Reference	​	Reference	​
High	165	1.430 (1.032–1.981)	0.032	0.888 (0.521–1.513)	0.663
SKA3	329	​	​	​	​
Low	165	Reference	​	Reference	​
High	164	1.474 (1.068–2.035)	0.018	1.073 (0.688–1.674)	0.757
SPAG5	329	​	​	​	​
Low	164	Reference	​	Reference	​
High	165	1.415 (1.024–1.956)	0.036	0.830 (0.486–1.417)	0.494
SPC25	329	​	​	​	​
Low	165	Reference	​	Reference	​
High	164	1.415 (1.024–1.954)	0.035	0.937 (0.574–1.528)	0.793
TK1	329	​	​	​	​
Low	164	Reference	​	Reference	​
High	165	1.406 (1.018–1.943)	0.039	0.917 (0.575–1.463)	0.717
TONSL	329	​	​	​	​
Low	164	Reference	​	Reference	​
High	165	1.472 (1.059–2.045)	0.021	1.115 (0.728–1.707)	0.617
TPX2	329	​	​	​	​
Low	164	Reference	​	Reference	​
High	165	1.413 (1.021–1.957)	0.037	0.869 (0.501–1.507)	0.617
TROAP	329	​	​	​	​
Low	165	Reference	​	Reference	​
High	164	1.448 (1.044–2.009)	0.027	0.836 (0.489–1.429)	0.512
UBE2T	329	​	​	​	​
Low	165	Reference	​	Reference	​
High	164	1.591 (1.143–2.214)	0.006	1.295 (0.684–2.451)	0.427
WDR76	329	​	​	​	​
Low	164	Reference	​	Reference	​
High	165	1.430 (1.035–1.975)	0.030	1.006 (0.586–1.726)	0.984
ZWINT	329	​	​	​	​
Low	165	Reference	​	Reference	​
High	164	1.398 (1.010–1.936)	0.043	0.906 (0.561–1.463)	0.685

### 3.2 Assessment of Differential Gene expression, survival analysis, and ROC Curve Analysis

**FIGURE 2:**
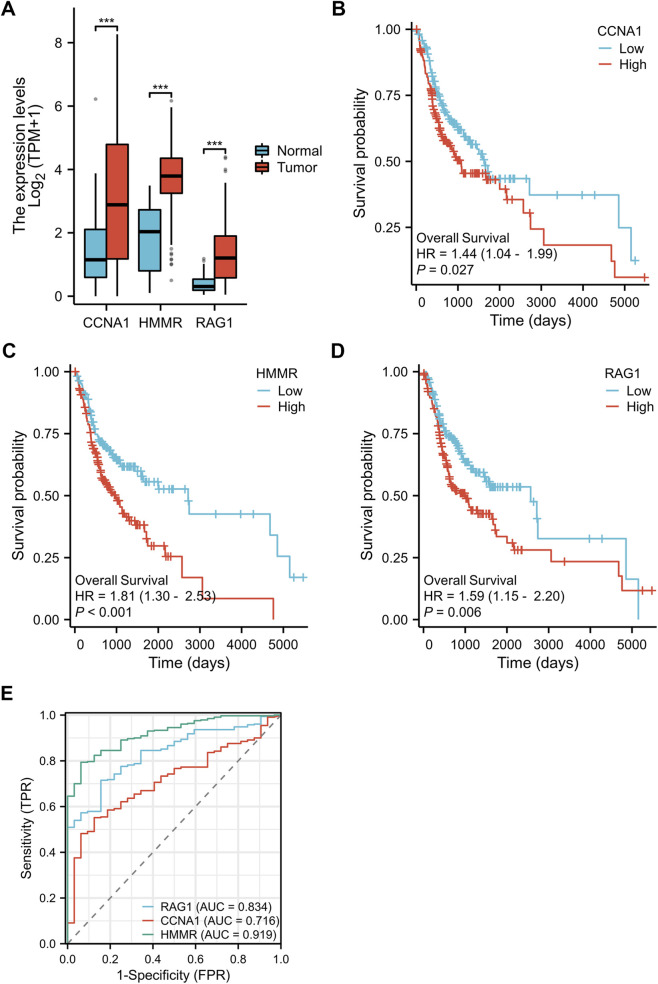
Expression, Survival, and ROC Curve Analysis of Differential Genes in OSCC. **(A)** Box plots compare the expression levels of CCNA1, HMMR, and RAG1 in OSCC tissues with those in normal tissues, demonstrating significant overexpression of all three genes in OSCC tissues compared to normal tissues (*P < 0.001). **(B–D)** Kaplan-Meier survival curves demonstrate the correlation between high expression levels of CCNA1 **(B)**, HMMR **(C)**, and RAG1 **(D)** and poorer overall survival (OS) among OSCC patients. **(E)** ROC curves, along with their respective area under the curve (AUC) values, assess the predictive capabilities of CCNA1, HMMR, and RAG1 in identifying OSCC using TCGA data.

To assess the correlation between the expression of the aforementioned genes and overall survival in OSCC patients, we retrieved and analyzed the gene expression data from the TCGA OSCC cohort. The findings demonstrated that the expression levels of CCNA1, HMMR, and RAG1 were significantly elevated in comparison to their expression in normal tissues (Figure 2A, ***P < 0.001). Survival analysis further corroborated this observation, indicating a significant association between high expression of these three genes and poorer overall survival (OS) in patients (Figures 2B–D). Additionally, a receiver operating characteristic curve (ROC) analysis was conducted on the TCGA OSCC dataset. The ROC curves for CCNA1, HMMR, and RAG1 were analyzed, and the areas under the curve (AUC) within the TCGA dataset were 71.6%, 91.9%, and 83.4%, respectively. Notably, HMMR exhibited the highest predictive capacity for prognosis in the TCGA cohort (Figure 2E).

### 3.3 Analysis of HMMR expression and its association with survival in TCGA OSCC cohort

**FIGURE 3:**
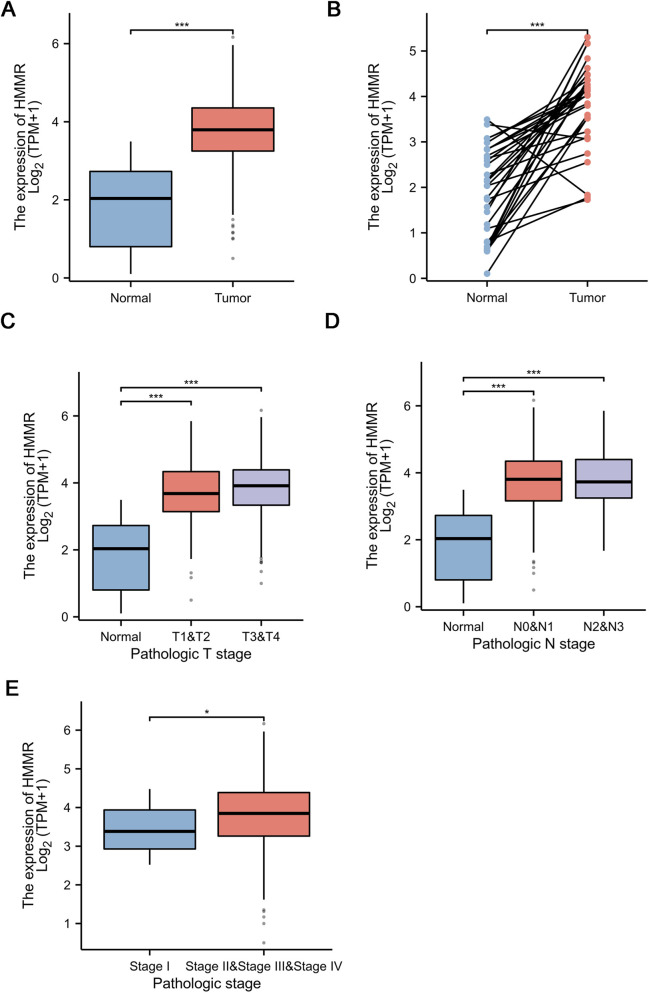
Expression and Correlation Analysis of HMMR in TCGA-OSCC Cohort. **(A)** A box plot compares the expression levels of HMMR in paired tumor and normal tissues from the TCGA-OSCC cohort, revealing a significant increase in HMMR expression in tumor tissues compared to normal tissues (***P < 0.001). **(B)** A box plot illustrates the expression levels of HMMR in non-paired tumor and normal tissues from the TCGA-OSCC cohort. Similarly, a significant elevation in HMMR expression is observed in tumor tissues (***P < 0.001). **(C–E)** Bar charts or scatter plots depicting the correlation between elevated HMMR expression and various clinicopathological features within the TCGA-OSCC cohort. Specifically, the association with T stage **(C)**, N stage **(D)**, and pathological grade **(E)** is explored. Statistically significant associations are indicated with asterisks (*P < 0.05, ***P < 0.001).

An extensive investigation of HMMR expression across multiple normal tissue cohorts revealed that its expression in the oral cavity ranks sixth among other tissues (Supplementary Figure S2). Furthermore, a pan-cancer analysis of the TCGA dataset demonstrated that HMMR is overexpressed in the majority of tumor tissues (Supplementary Figure S3). Notably, in the TCGA OSCC cohort, both paired and unpaired tissue comparisons demonstrated a significant elevation in HMMR expression (Figures 3A,B, ***P < 0.001). This overexpression of HMMR was found to be significantly associated with advanced T stage, N stage, and pathological grade (Figures 3C–E, *P < 0.05, ***P < 0.001).

**FIGURE 4:**
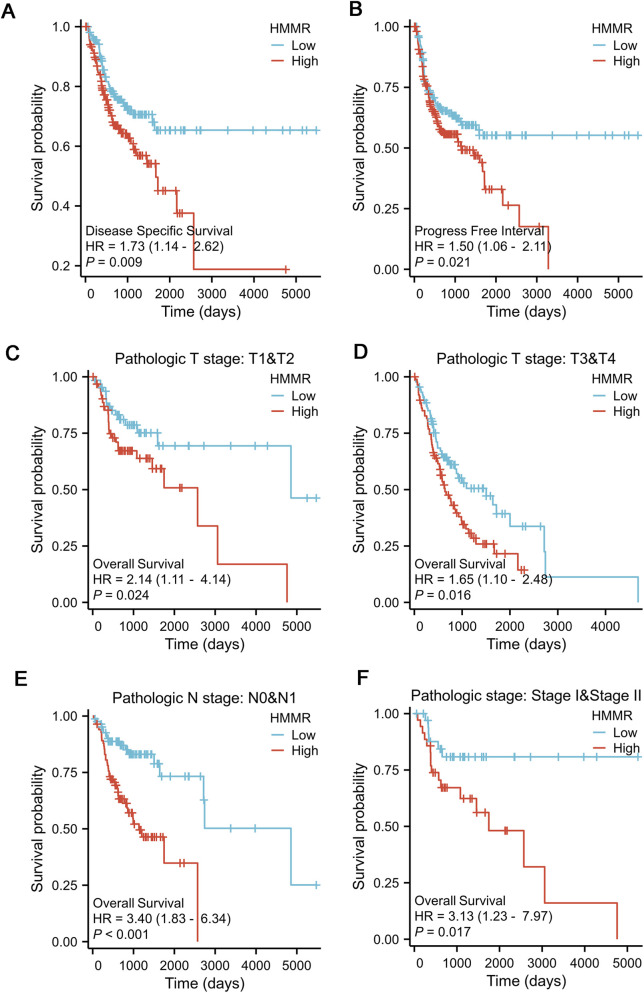
Survival Analysis Stratified by HMMR Expression in TCGA-OSCC Cohort. **(A,B)** Kaplan-Meier survival curves illustrating the impact of high HMMR expression on disease-specific survival **(A)** and disease-free survival **(B)** in the TCGA-OSCC cohort. **(C–F)** Forest plots present the univariate analysis of key factors, including T stage **(C)**, N stage **(D)**, pathological grade **(E)**, and HMMR expression **(F)**, in predicting overall survival in the TCGA-OSCC cohort. Hazard ratios (HRs) and 95% confidence intervals (CIs) are provided for each factor.

To further elucidate the prognostic significance of HMMR, we conducted a comprehensive survival analysis. Our findings indicated that patients with high HMMR expression exhibited significantly shorter disease-specific survival and disease-free survival compared to those with low expression (Figures 4A,B). Additionally, survival analysis revealed that T stage, N stage, pathological grade, and HMMR expression are predictive factors for overall survival in the TCGA OSCC cohort (Figures 4C–F). Single cell sequencing analysis also revealed that HMMR gene expressions were enriched in the malignant cells and CD4^+^/CD8^+^ T cells (Supplementary Figure S4).

### 3.4 Independent validation of HMMR expression and prognostic value in GEO cohorts

To further validate the robustness of our findings from the TCGA-OSCC cohort, we performed independent validation using two publicly available GEO datasets: GSE37991 (expression validation) and GSE41613 (prognostic validation). In the GSE37991 cohort, which included 45 paired OSCC tumor tissues and adjacent normal oral mucosa tissues, HMMR expression was significantly upregulated in OSCC tumor tissues compared with normal controls (t-test, P = 8.55 × 10^−7^, Supplementary Figure S5A). This result was highly consistent with our TCGA analysis, confirming the universal overexpression of HMMR in OSCC. Subsequently, we evaluated the prognostic value of HMMR in the GSE41613 cohort, which contains 97 OSCC patients with complete overall survival (OS) follow-up data. Patients were divided into high and low HMMR expression groups using the optimal cutoff value (8.055) determined by the maximally selected rank statistics. Kaplan-Meier survival analysis showed that patients with high HMMR expression had significantly shorter OS than those with low HMMR expression (Log-rank P = 0.0096, Supplementary Figure S5B). The hazard ratio (HR) for OS in the high HMMR expression group was 1.92 (95% CI: 1.13–3.26), which was comparable to the HR value observed in the TCGA cohort (HR = 1.81, 95% CI: 1.30–2.53). Collectively, these independent validation results from two distinct GEO cohorts strongly supported our conclusion that HMMR was significantly overexpressed in OSCC and served as a robust prognostic biomarker for poor OS in OSCC patients.

### 3.5 Correlation analysis between HMMR expression and immune cell infiltration in OSCC

**FIGURE 5:**
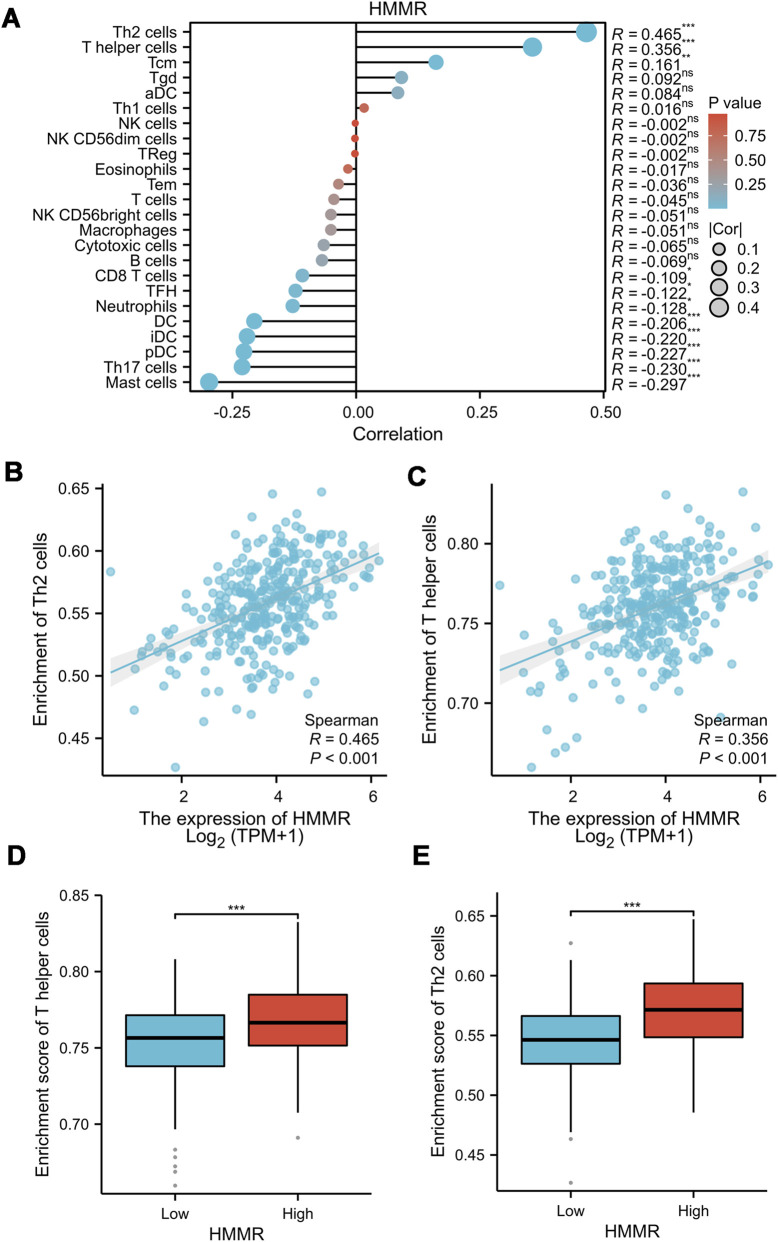
Correlation Analysis of HMMR Expression with Immune Cell Infiltration Patterns in OSCC. **(A)** A heatmap visualizes the differential infiltration abundances of 11 immune cell types between high and low HMMR expression groups in OSCC. Significant differences are represented by distinct color gradients. **(B,C)** Scatter plots reveal a positive correlation between HMMR expression and the infiltration levels of Th2 cells **(B)** and T helper cells **(C)** in OSCC. Correlation coefficients and their corresponding significance levels are indicated. **(D,E)** Box plots compare the infiltration levels of Th2 cells **(D)** and T helper cells **(E)** in the high and low HMMR expression groups, with significant differences denoted by asterisks.

Immune infiltration analysis revealed statistically significant differences in the infiltration of 11 immune cell subsets between patients with high and low HMMR expression (Figure 5A). Specifically, HMMR expression was significantly positively correlated with the infiltration of Th2 cells (r=0.465, P < 0.001), T helper cells (r=0.356, P < 0.001), and Tcm cells (r=0.161, P < 0.01). In contrast, HMMR expression was significantly negatively correlated with the infiltration of Mast cells (r=−0.297, P < 0.001), Th17 cells (r=−0.230, P < 0.001), pDC cells (r=−0.227, P < 0.001), iDC cells (r=−0.220, P < 0.001), DC cells (r=−0.206, P < 0.001), Neutrophils (r = -0.128, P < 0.001), TFH cells (r=−0.122, P < 0.01), and CD8 T cells (r=−0.109, P < 0.05). No significant correlation was observed between HMMR expression and the infiltration of Tgd cells, aDC cells, Th1 cells, NK cells, NK CD56dim cells, Treg cells, Eosinophils, Tem cells, T cells, NK CD56bright cells, Macrophages, Cytotoxic cells, or B cells (all P > 0.05).The positive correlation between HMMR and Th2/T helper cells suggested that HMMR may contribute to the formation of an immunosuppressive tumor microenvironment in OSCC. The negative correlation with Mast cells and NK cells further may support this notion, as these cells play important roles in anti-tumor immunity.

Notably, the infiltration levels of Th2 cells and T helper cells were **significantly positively correlated** with HMMR expression, with Spearman correlation coefficients of 0.465 (P < 0.001) and 0.356 (P < 0.001), respectively (Figures 5B,C). Consistently, the enrichment scores of both T helper cells and Th2 cells were significantly higher in the HMMR high-expression group than in the low-expression group (both P < 0.001, Figures 5D,E).

### 3.6 Signaling pathway enrichment analysis of HMMR in OSCC

**FIGURE 6:**
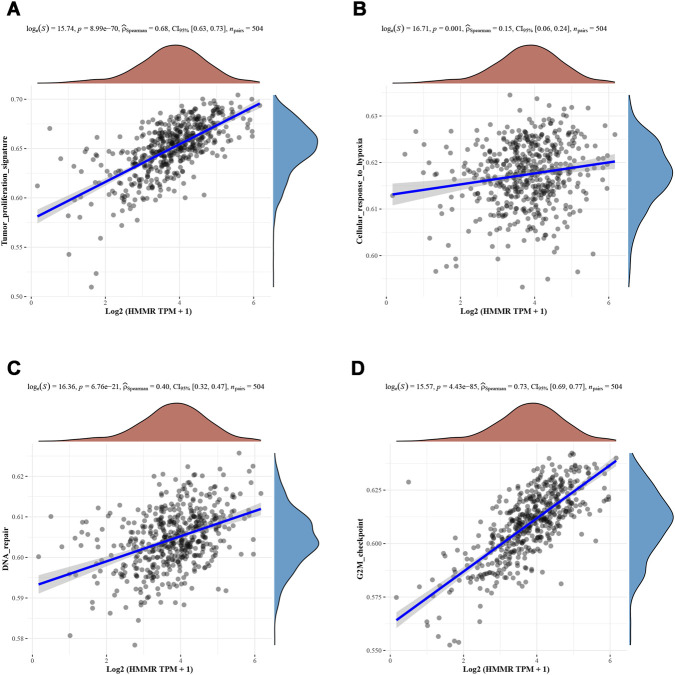
Gene set enrichment analysis (GSEA) of HMMR-associated signaling pathways in OSCC. HMMR was significantly associated with **(A)** tumor proliferation signature, **(B)** cellular response to hypoxia, **(C)** DNA repair, and **(D)** G2/M checkpoint pathways.

To explore the signaling pathways mediated by HMMR in OSCC, we performed gene set enrichment analysis (GSEA) using the clusterProfiler package in R. Genes were ranked by their correlation coefficient with HMMR expression, and the significance threshold was set as padj <0.05 and normalized enrichment score (NES) > 1.5. The results showed that HMMR was significantly associated with multiple oncogenic signaling pathways, including tumor proliferation signature (Spearman r=0.68, P=8.99 × 10^−70^), cellular response to hypoxia (Spearman r=0.15, p=0.001), DNA repair (Spearman r=0.40, p=6.76e-21), and G2M checkpoint (Spearman r=0.73, p=4.43e-85) (Figure 6). All correlations were analyzed in 362 OSCC samples from the TCGA cohort. These results suggested that HMMR may promote OSCC progression by regulating tumor cell proliferation, hypoxic stress response, or cell cycle. Protein expression analysis also revealed that HMMR was highly expressed in OSCC clinical samples (Supplementary Figure S6).

### 3.7 HMMR expression in the PPI network in OSCC

**FIGURE 7:**
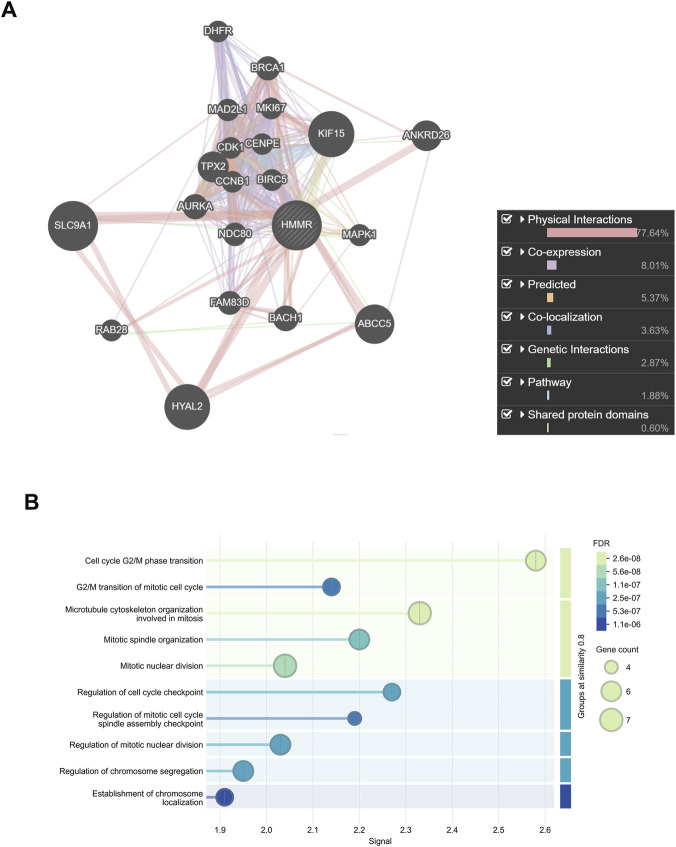
PPI network and functional enrichment of HMMR-related genes. **(A)** PPI network of 21 HMMR-interacting genes, with HMMR as the core node. **(B)** Top nine significantly enriched Gene Ontology (GO) biological process terms for the 21-gene PPI network. The x-axis represents the enrichment factor (Signal value), bubble color indicates the false discovery rate (FDR), and bubble size represents the number of enriched genes.

To explore the potential biological functions mediated by HMMR in OSCC, we performed a thorough screening of HMMR-associated interactions in the GeneMANIA database. We focused on multiple interaction types, namely, physical interactions, co - expression, predicted interactions, co-localization, pathway participation, genetic interactions, and shared protein domains. This detailed analysis enabled us to identify a complex network consisting of proteins that interact with HMMR. The interacting proteins include DHFR, BRCA1, MAD2L1, MKI67, KIF15, ANKRD26, CDK1, CENPE, TPX2, CCNB1, BIRC5, AURKA, SLC9A1, NDC80, MAPK1, FAM83D, BACH1, RAB28, and HYAL2. Subsequently, we constructed a network diagram to vividly illustrate these elaborate interactions (Figure 7A).

Functional enrichment analysis showed that the PPI network of the 21 genes was significantly enriched in tumor proliferation-related pathways, including cell cycle regulation, mitotic progression, spindle assembly, and chromosome segregation, which was highly consistent with the classic role of HMMR in tumor cell cycle regulation. Key node genes such as HMMR, CDK1, CCNB1, AURKA, and TPX2 were all critical regulators of G2/M phase transition and spindle assembly, and their enrichment results further validated the central role of this PPI network in tumor proliferation.

Collectively, the biological functions related to this HMMR-interacting network covered a variety of processes, such as cell cycle regulation, DNA repair, and mitotic progression. These findings contributed to a better understanding of the potential roles of HMMR and its interacting partners in the biological processes relevant to OSCC.

## 4 Discussion

Oral Squamous Cell Carcinoma (OSCC) was one of the most common malignant tumors in the world, and its incidence and mortality ranks first among oral cancer diseases (Barsouk et al., 2023). Despite significant advances in diagnostic and therapeutic techniques in recent years, the 5-year survival rate for OSCC was still below 50%, mainly due to the difficulty of early diagnosis and resistance to radiotherapy and chemotherapy. Therefore, it is of great significance to deeply explore the pathogenesis of OSCC and find new therapeutic targets to improve the survival rate of OSCC patients.

Hyaluronic acid (HA) was a macromolecular polysaccharide that was widely distributed in various tissues of the human body, especially connective tissue. HA played an important role in maintaining the stability of tissue structure and function, regulating cell proliferation and migration, and participating in physiological processes such as inflammatory response (Zheng et al., 2023). Contemporary research has elucidated that HA and its associated receptors were instrumental in instigating and fostering a multitude of neoplasms, encompassing breast cancer, colorectal cancer, gastric cancer, and nasopharyngeal carcinoma (Bhattacharyya et al., 2023).

The Hyaluronan-Mediated Motility Receptor (HMMR), a principal receptor of HA, was ubiquitously expressed across diverse cellular surfaces. The correlation between the expression of HMMR and immune cell infiltration was an intriguing aspect that deserved careful examination (Jiang et al., 2011). Given the complexity of the immune system and the diverse functions of immune cells, it was crucial to understand how HMMR expression may influence the infiltration patterns of these cells in OSCC. In the present analysis, we observed statistically significant differences in the infiltration of 11 immune cell subsets between patients with high and low HMMR expression (Figure 5).

Notably, the infiltration of Th2 cells and T helper cells positively correlated with HMMR expression. This finding suggested that the expression level of HMMR may modulate the recruitment and infiltration of specific immune cell types in OSCC. Th2 cells, known for their role in humoral immunity, were associated with antibody production and allergic responses (Lozano-Ojalvo et al., 2023). Their positive correlation with HMMR expression may indicate a role in promoting a pro-tumorigenic immune environment in OSCC. Similarly, T helper cells, which orchestrated immune responses by regulating the activation of other immune cells, may also contributed to the tumor-promoting immune microenvironment when their infiltration was associated with high HMMR expression (Lei et al., 2023). It was important to note that the cell-autonomous and non-cell-autonomous mechanisms mediated by HMMR were not mutually exclusive but were closely interconnected. High HMMR expression may drive excessive proliferation of OSCC cells by dysregulating the cell cycle, which lead to increased metabolic demand and hypoxia in the tumor microenvironment. Hypoxia, in turn, upregulates the expression of HMMR and various chemokines, which further promote tumor cell invasion and recruit immunosuppressive immune cells such as Th2 cells. Conversely, the immunosuppressive tumor microenvironment created by these immune cells allows tumor cells to evade immune surveillance and continue to proliferate uncontrollably. This vicious cycle between tumor cell proliferation and immune suppression contributes to the aggressive progression and poor prognosis of OSCC. Our study provided a comprehensive view of the dual role of HMMR in OSCC and lays the foundation for the development of novel therapeutic strategies targeting both tumor cells and the tumor immune microenvironment. Future studies are needed to elucidate the molecular mechanisms underlying this correlation and to determine whether targeting HMMR can modulate immune cell infiltration and, thereby, influence the progression and prognosis of OSCC.

As for signaling pathway associated with HMMR, upon binding with HA, HMMR was capable of initiating an array of signaling cascades, thereby modulating cellular proliferation, migration, and apoptosis (Rizzardi et al., 2014). To the best of our knowledge, this is the first comprehensive study to systematically investigate the expression, prognostic value, and biological functions of HMMR in OSCC, which may expedite the onset and progression of OSCC via DNA damage repair and hypoxia signaling (Figure 6). This discovery furnished us with a novel therapeutic target and holds promise for a groundbreaking strategy for the precocious diagnosis and treatment of OSCC. In the PPI network, we also found that beyond just visualizing the network, we further investigated the biological significance of these identified proteins (Figure 7). For example, BRCA1 was well-known for its role in DNA repair and maintaining genomic stability. MKI67 was a widely used proliferation marker. AURKA was also involved in cell cycle regulation and mitosis. CDK1 plays a crucial part in cell cycle progression. Collectively, the biological functions related to this HMMR-interacting network covered a variety of processes, such as cell cycle regulation, DNA repair, and mitotic progression, which was consistent with the previous predictive analysis of the enriched signaling pathway. HMMR may confer chemoresistance and radioresistance in OSCC by regulating G2/M checkpoint and DNA repair pathways. Nonetheless, the precise mechanism of HMMR in OSCC warrants further exploration.

This study had several limitations that should be acknowledged. First, this was a retrospective bioinformatics study based solely on public databases. Although we validated our findings using an independent GEO dataset, the results still need to be confirmed in large, multi-center clinical cohorts. Second, the TCGA-OSCC dataset was mainly composed of patients of European descent, which may introduce population bias and limit the generalizability of our findings to other ethnic groups. Third, OSCC was a heterogeneous disease that arise from different anatomical sites in the oral cavity. Our study did not analyze the differences in HMMR expression and function between OSCCs from different sites. Fourth, all our findings were based on computational predictions, and the exact molecular mechanisms by which HMMR regulates cell cycle, DNA repair, and immune infiltration in OSCC remain to be elucidated through experimental studies.

Future research directions include: Validating HMMR expression in clinical OSCC samples using immunohistochemistry and qRT-PCR, and analyzing its correlation with clinicopathological features and prognosis; Investigating the functional role of HMMR in OSCC using *in vitro* cell culture models and *in vivo* xenograft models; Elucidating the molecular mechanisms by which HMMR interacts with other proteins to regulate cell cycle and DNA repair using co-immunoprecipitation and immunofluorescence assays; Exploring the effect of HMMR inhibition on the sensitivity of OSCC cells to radiotherapy and chemotherapy; Investigating the paracrine mechanisms by which HMMR modulates immune cell infiltration in the tumor microenvironment.

## 5 Conclusion

In conclusion, our study demonstrated that HMMR was overexpressed in OSCC and served as an independent prognostic biomarker. HMMR may promote OSCC progression through dual mechanisms: driving cell cycle dysregulation and modulating the immunosuppressive tumor microenvironment.

## Data Availability

The original contributions presented in this study are publicly available. The data can be found in the Gene Expression Omnibus (GEO) repository under the accession numbers GSE37991, GSE41613, and GSE103322.

## Supplementary material

SUPPLEMENTARY FIGURE S1 GO/KEGG analysis of 245 common DEGs in OSCC.

SUPPLEMENTARY FIGURE S2 HMMR expression across multiple normal tissue cohorts.

SUPPLEMENTARY FIGURE S3 Pan-cancer analysis of HMMR expression in TCGA database.

SUPPLEMENTARY FIGURE S4 Single cell sequencing analysis of HMMR expressions in GSE103322 dataset.

SUPPLEMENTARY FIGURE S5 Independent validation of HMMR expression and prognostic value in GEO cohorts.

SUPPLEMENTARY FIGURE S6 Protein expression analysis of HMMR in The Human Protein Atlas database.

SUPPLEMENTARY TABLE S1 List of Differentially Expressed Genes and Their Expression Patterns in OSCC.

SUPPLEMENTARY TABLE S2 Functional Annotations and Enrichment Analysis of Selected Genes in OSCC.

SUPPLEMENTARY TABLE S3 GO KEGG analysis of DEGs.
