# Supplementary figures and images for: HMMR as a robust prognostic biomarker correlates with immune infiltration and cell cycle pathways in oral squamous cell carcinoma: a multi-cohort bioinformatics analysis based on TCGA and GEO databases
Source: Front Genet. 2026 Jun 5;17:1764943. doi: 10.3389/fgene.2026.1764943 (PMC13278685; doi:10.3389/fgene.2026.1764943)

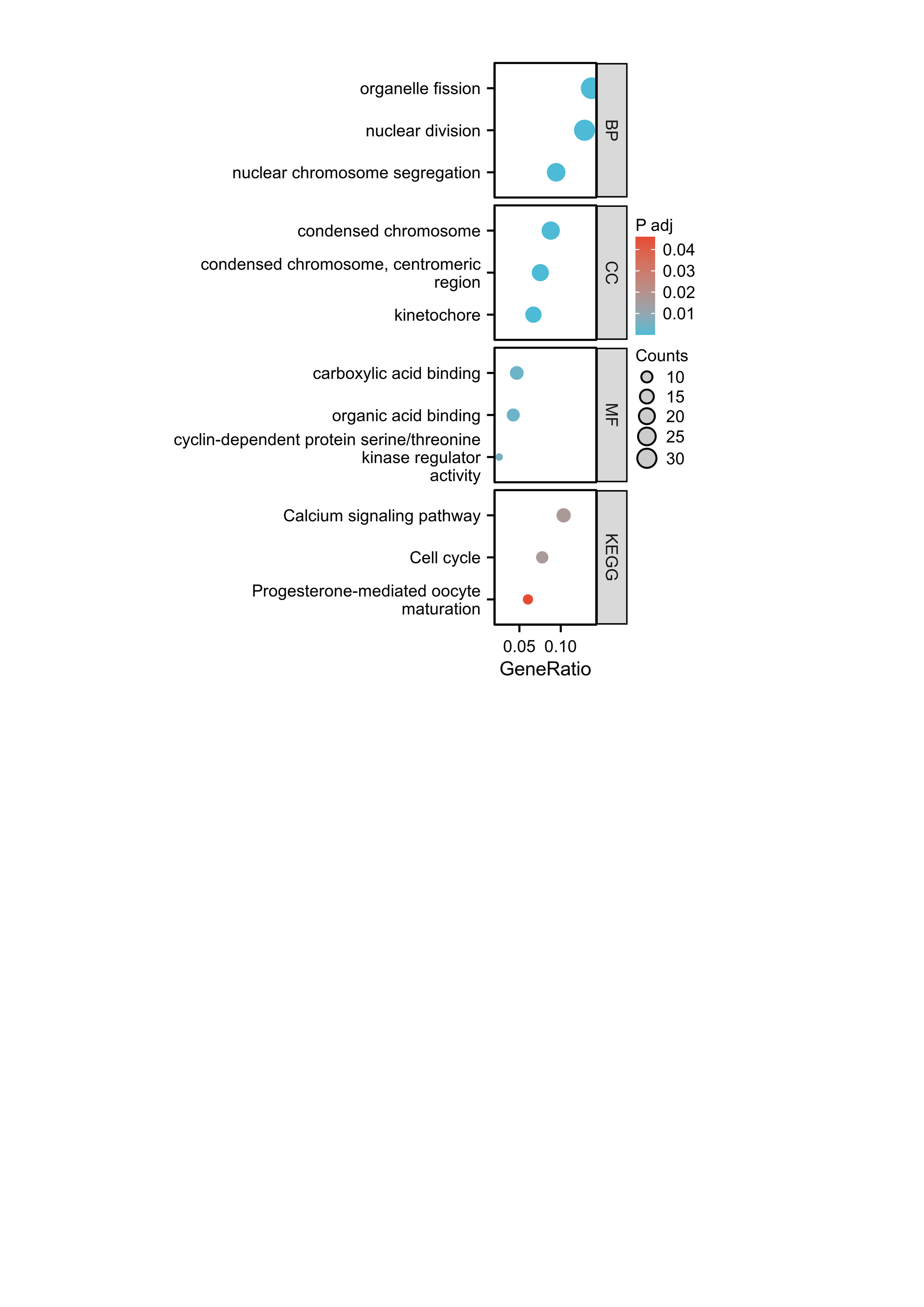

Supplement: Supplementary file 1 [file Image1.tiff]

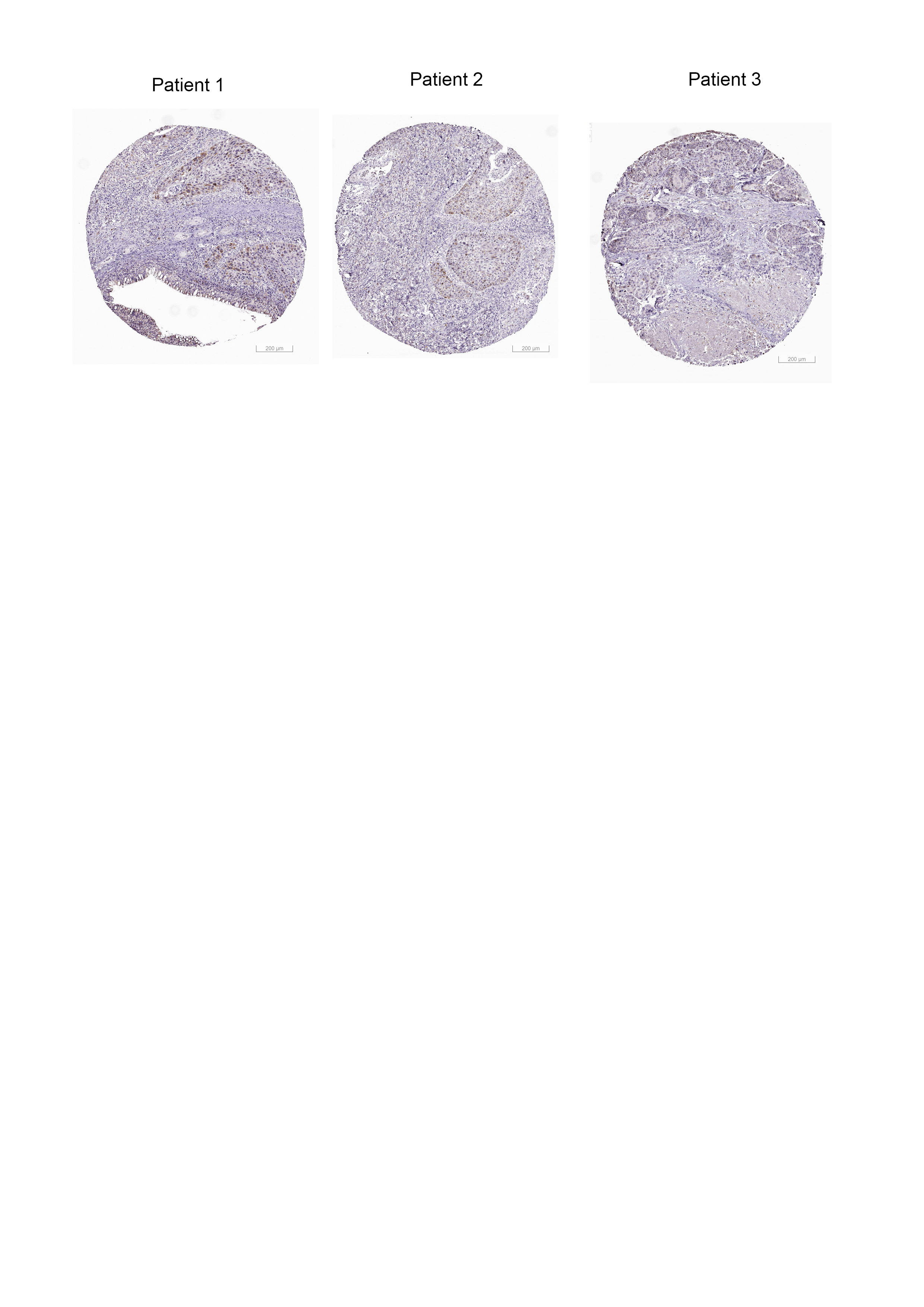

Supplement: Supplementary file 3 [file Image6.tif]

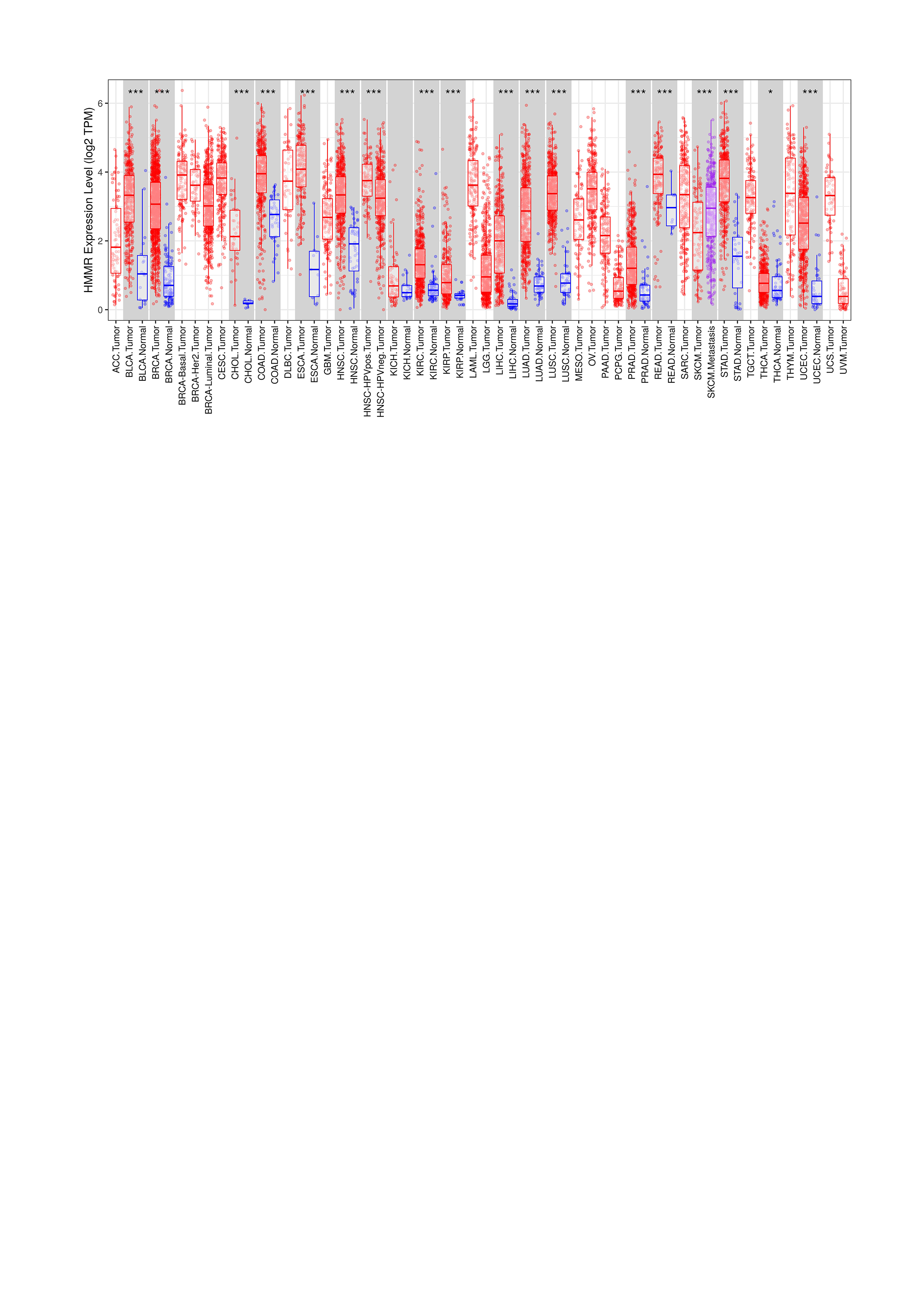

Supplement: Supplementary file 5 [file Image3.tif]

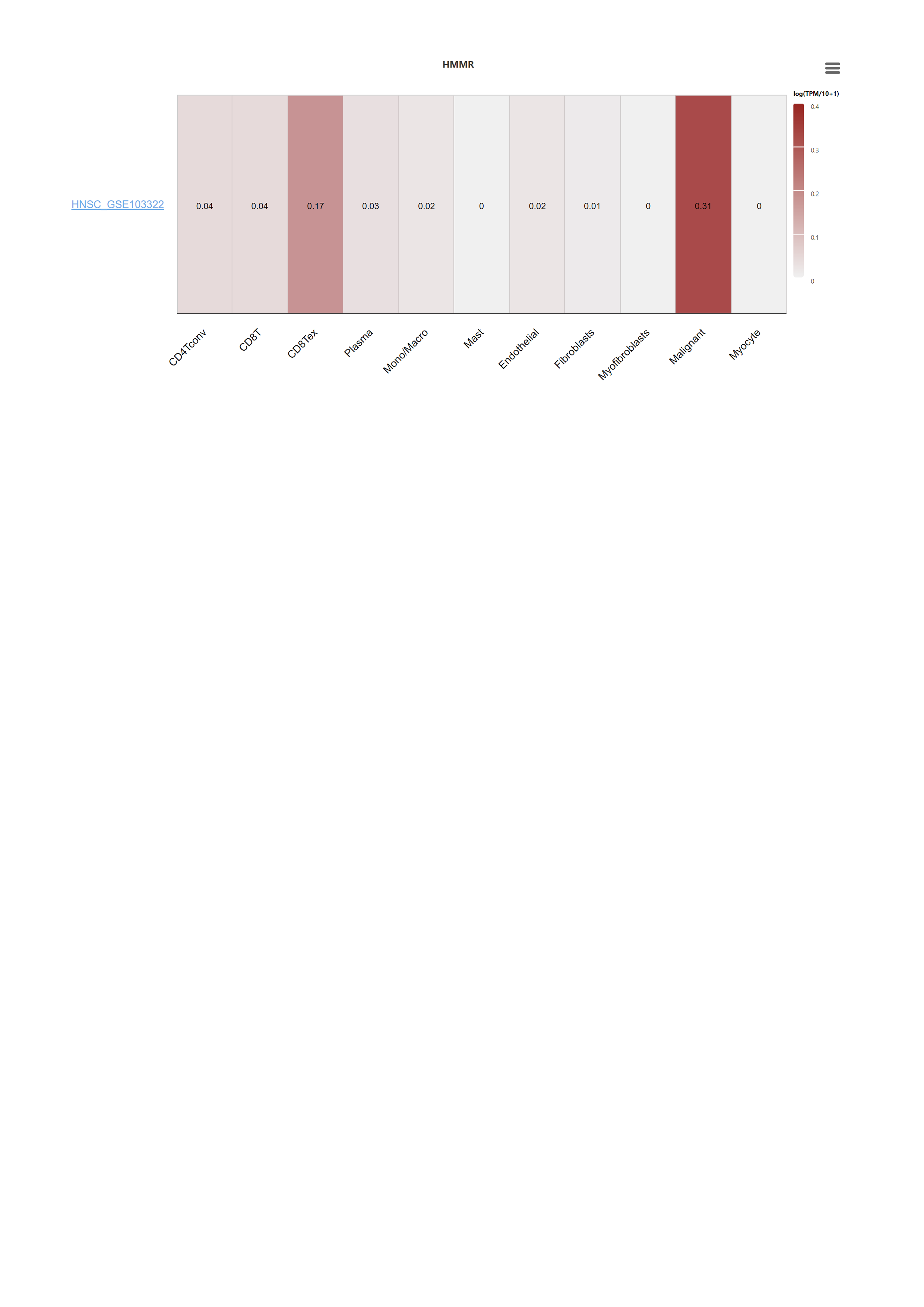

Supplement: Supplementary file 6 [file Image4.tif]

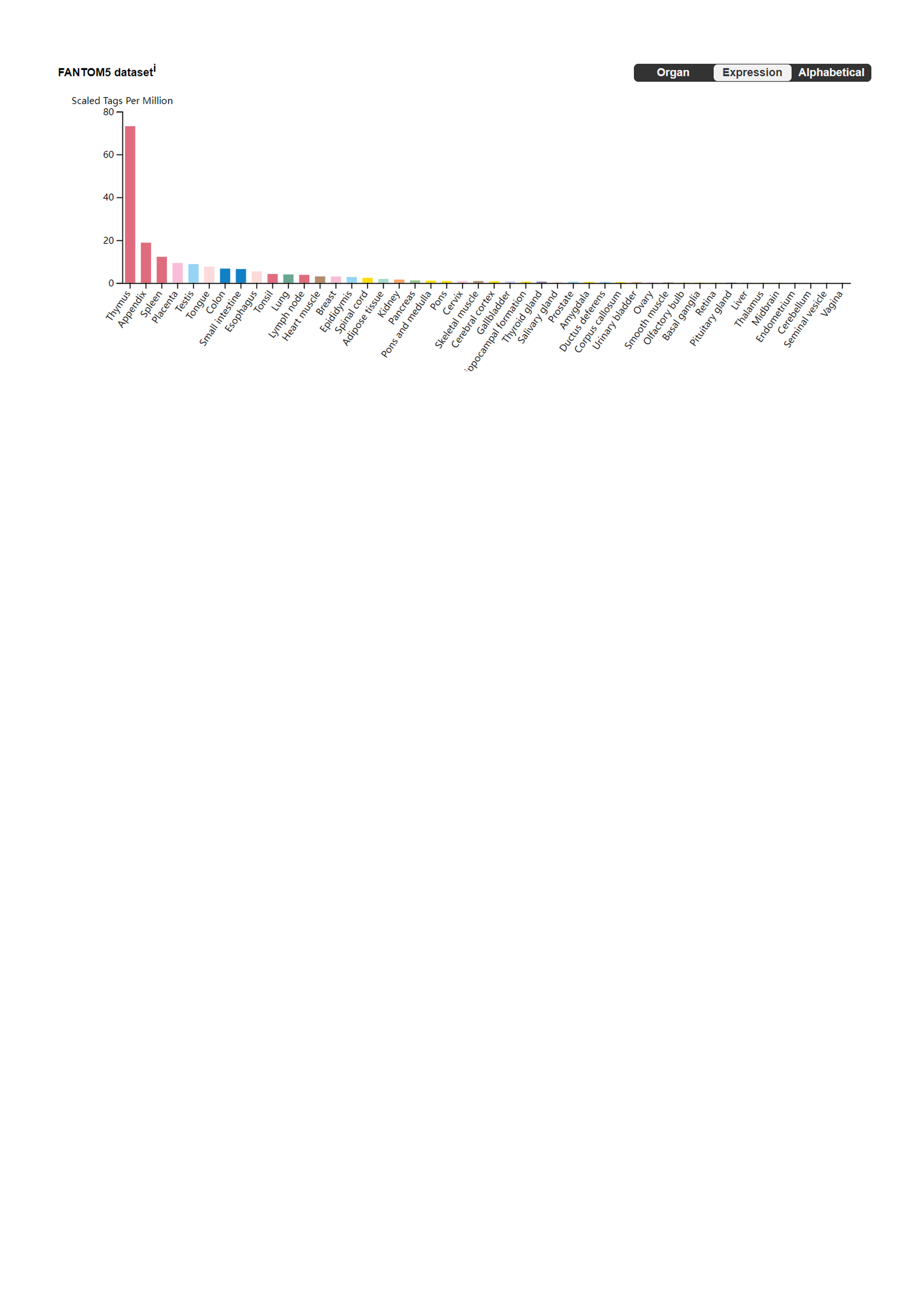

Supplement: Supplementary file 7 [file Image2.tif]

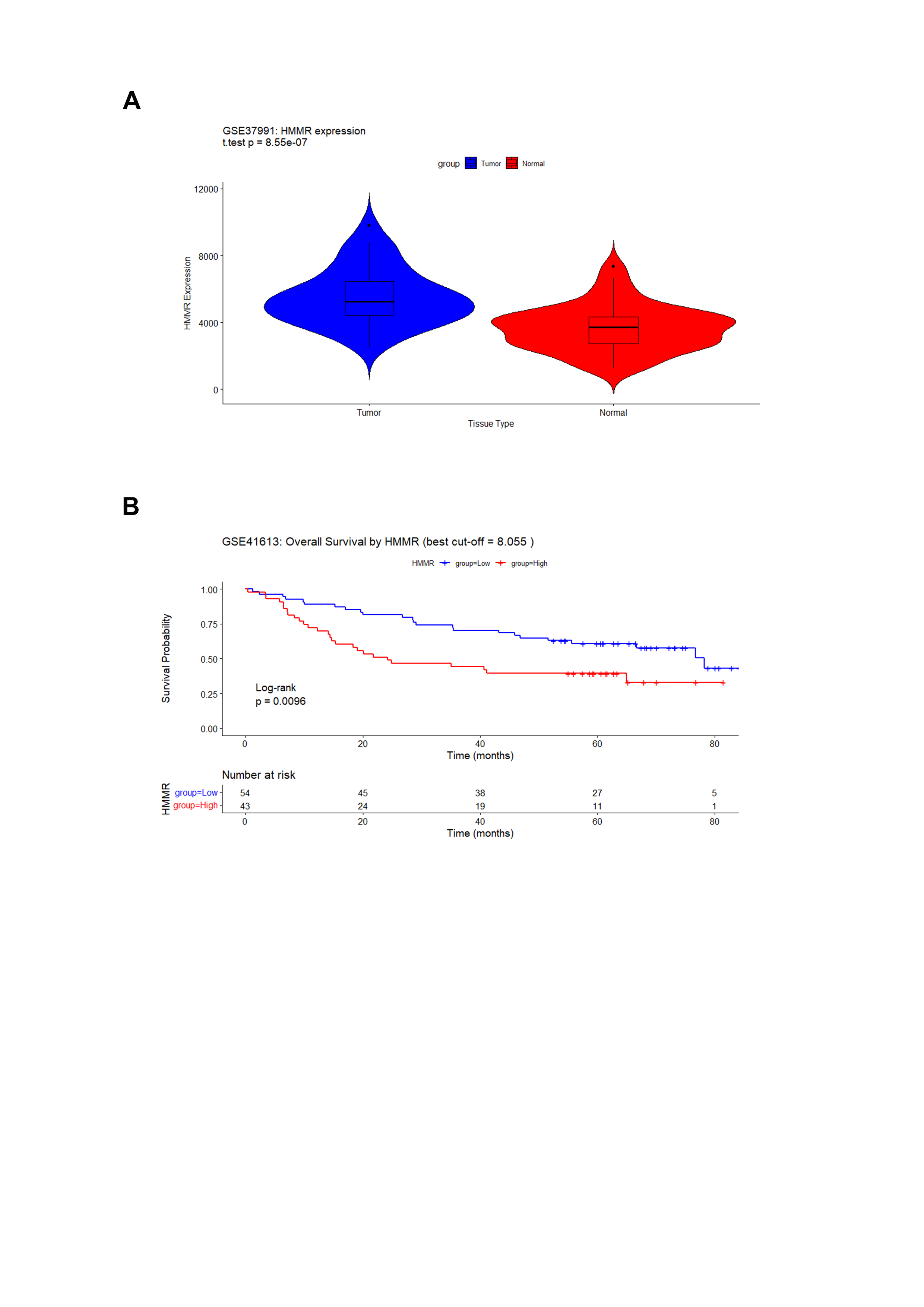

Supplement: Supplementary file 9 [file Image5.tif]
