# Supplementary material for: HMMR as a robust prognostic biomarker correlates with immune infiltration and cell cycle pathways in oral squamous cell carcinoma: a multi-cohort bioinformatics analysis based on TCGA and GEO databases
Source: Front Genet. 2026 Jun 5;17:1764943. doi: 10.3389/fgene.2026.1764943 (PMC13278685; doi:10.3389/fgene.2026.1764943)
Supplement: Supplementary file 4 [file Table1.docx]

| **DEGs** | **Prognosis** | **Common** |
| --- | --- | --- |
| TSPAN6 | TSPAN6 | TSPAN6 |
| C1orf112 | DPM1 | CELSR3 |
| ENPP4 | BAD | NCAPD2 |
| CFTR | AOC1 | ATP1A2 |
| HS3ST1 | M6PR | DEPDC1 |
| CYP26B1 | NDUFAF7 | DEPDC1B |
| ICA1 | FKBP4 | RTN4R |
| DBNDD1 | NDUFAB1 | ZIC2 |
| SLC7A2 | CDC27 | PPP1R3F |
| HSPB6 | RPAP3 | RAD51 |
| PDK4 | REXO5 | PDIA5 |
| PRKAR2B | CIAPIN1 | ISOC1 |
| TSPOAP1 | GDE1 | MSANTD3 |
| WDR54 | CRLF1 | SYT1 |
| PDK2 | GGCT | PRR11 |
| ITGA3 | COX10 | SPEG |
| TMEM132A | MARK4 | ALDH3A2 |
| CX3CL1 | CEACAM21 | HMMR |
| TNFRSF12A | CD79B | IGF2BP2 |
| DLX6 | BAIAP3 | SPAG5 |
| ETV1 | NOX1 | UBE2T |
| ALDH3B1 | ADAM22 | SMARCD3 |
| CCL26 | SYPL1 | PLOD1 |
| ARHGAP44 | CELSR3 | CTTN |
| SCIN | MED24 | MGST2 |
| PROM1 | SEC62 | RAD54L |
| NOS2 | TENM1 | AURKA |
| GAS7 | TRAF3IP3 | TPX2 |
| CEACAM7 | MLXIPL | CHGB |
| SCN4A | ZNF207 | KIF4A |
| SELE | UQCRC1 | LAMB4 |
| CELSR3 | NCAPD2 | ORC6 |
| PLEKHG6 | LRRC23 | WDR76 |
| MGST1 | HFE | CDC6 |
| NFIX | UTP18 | SEC14L2 |
| HHATL | TMEM159 | CENPM |
| NCAPD2 | KDM5D | CTSG |
| SEMA3G | PSMC4 | CDKN3 |
| PRSS3 | SLC25A39 | ASB2 |
| FMO1 | POLR3B | MRGBP |
| ABHD5 | RNF14 | TRIB3 |
| DPF1 | HEBP1 | TNNC2 |
| ANLN | MAMLD1 | CENPI |
| BRCA1 | CD6 | OLFM4 |
| LTF | ZC3H3 | SFRP1 |
| EHD3 | MDH1 | NDRG1 |
| DDX11 | COX15 | CKM |
| TACC3 | BID | ASF1B |
| POLA2 | XYLT2 | CNFN |
| ACP3 | ATP1A2 | CAV1 |
| CHDH | HGF | MET |
| CLCA4 | MNAT1 | HOXA1 |
| SLC38A5 | RNF10 | CYP3A5 |
| SLC11A1 | ZNF839 | AGFG2 |
| ATP1A2 | GRAMD1B | PRUNE2 |
| VSIG2 | ERP44 | CA9 |
| SNAI2 | STRAP | NPDC1 |
| PLEKHB1 | GCLM | SFXN3 |
| SERPINB1 | DEPDC1 | DKK1 |
| FHL1 | TOMM34 | SGCA |
| NDUFS1 | MIPEP | NCAPG |
| EHD2 | SNX1 | ALDH5A1 |
| DEPDC1 | BCLAF1 | GMNN |
| RRAGD | BAK1 | ARFGEF3 |
| HSD17B6 | SARS1 | GMDS |
| TYMP | ALG1 | HBEGF |
| SH2D2A | DEPDC1B | CCNG1 |
| ANK1 | RPL26L1 | STC2 |
| HMGB3 | AGA | BCHE |
| IKZF2 | RIPOR1 | ECT2 |
| APBA2 | SPDL1 | CCL20 |
| TMSB10 | RTN4R | CENPA |
| DEPDC1B | RIPOR3 | TFAP2E |
| DAPK2 | TDP1 | NEK2 |
| MYOM2 | AIFM2 | CENPF |
| USP2 | ZIC2 | CASQ2 |
| TLL1 | TRIT1 | SPP1 |
| VCAN | DSG2 | GALNT12 |
| RAI14 | GPM6B | ALDH6A1 |
| RTN4R | TSPAN17 | GOT1 |
| TNC | LMO3 | CYSTM1 |
| BARX2 | MRPS10 | PLS1 |
| JADE2 | FAM120A | CLU |
| ZIC2 | VAMP3 | TNFRSF8 |
| PREX2 | UTS2 | PLAU |
| CP | PPP1R3F | P4HA1 |
| ROS1 | HEXB | ZWINT |
| ADAMTS6 | JKAMP | CENPK |
| TNFRSF9 | ARHGEF5 | CKS2 |
| LTBP1 | MAPK9 | ZBP1 |
| ELN | RAD51 | KLHL31 |
| FOXP3 | CYBA | CLYBL |
| PPP1R3F | HEBP2 | GRIA3 |
| NFE2L3 | MSMO1 | SIX1 |
| PTGER3 | CBLN4 | BEST3 |
| HOMER3 | NOP58 | AUNIP |
| RAD51 | CUL1 | KNSTRN |
| POLQ | FAM114A2 | PIMREG |
| LAMA3 | ATG5 | EGLN3 |
| KCNQ1 | PITHD1 | SGO1 |
| FOXC1 | POLR3E | TNNI2 |
| CHRDL2 | DGKG | PNCK |
| RELT | DNAJC25 | DUSP9 |
| EIF2AK2 | SLC2A3 | PSMC3IP |
| GYG2 | PTPRU | PPFIA1 |
| DCBLD2 | GPBP1 | PPARG |
| USP13 | CS | SEC61G |
| LAMC2 | EIF4B | ITGB4 |
| CAMK2B | ISOC2 | CLEC10A |
| PARP12 | INTS13 | PCBD2 |
| COL11A1 | TSPAN32 | CCNA1 |
| MPC1 | ATP2C2 | SAA2 |
| BCAT1 | TNPO3 | TROAP |
| CDH3 | NTHL1 | BORA |
| DGAT2 | GNAI3 | GPNMB |
| LIMCH1 | ROPN1 | ARHGEF39 |
| CCN5 | KARS1 | KNL1 |
| CDON | PDIA5 | ITPKA |
| SLC12A2 | TBC1D22B | HADHB |
| EYA2 | ZC3H15 | KIF11 |
| BCAS1 | MTHFD2 | CEP55 |
| CHI3L2 | SLC9A7 | MMRN1 |
| TRAM2 | ATXN3 | CENPE |
| MCM10 | ISOC1 | EGF |
| PDIA5 | MSANTD3 | BRCA2 |
| PACC1 | PFKP | ZIC5 |
| COL17A1 | DHX8 | RHCG |
| PRKCQ | SYT1 | UNC45B |
| TLE2 | IDH3G | TINAGL1 |
| FAM107B | ATP2B3 | NUF2 |
| SLC9A3 | HEATR6 | XPR1 |
| ASPM | COASY | S100A8 |
| MPPED2 | GRIPAP1 | CCNA2 |
| ISOC1 | PRR11 | CXCL14 |
| MSANTD3 | GNB5 | TPBG |
| STAG3 | HDHD5 | MTFR2 |
| PHKA1 | ELP1 | EGFR |
| CACNB1 | PFN2 | ADAM33 |
| SYT1 | FGF22 | ACSL1 |
| IFI35 | DGCR2 | SLC25A4 |
| PRR11 | JMJD6 | ATP5F1A |
| SIRT2 | CFAP20 | SPC25 |
| PYGM | CSNK2A2 | PFKM |
| PITX1 | SLC12A3 | GPD1L |
| SDK2 | RPL31 | PANK1 |
| ABCC9 | BUD23 | PLOD2 |
| MAOB | FAM3A | CABYR |
| RORA | PRKACA | OBSCN |
| TGFBR3 | SPEG | EME1 |
| HES2 | ALDH3A2 | CCNB2 |
| NUCB2 | HMMR | PWWP3B |
| SPTB | SCARB1 | TENT5B |
| CLTCL1 | IGF2BP2 | CDC25C |
| FSTL3 | NSF | MPZ |
| ST6GALNAC1 | HACD3 | FGFR4 |
| CNGB1 | ENO1 | TONSL |
| CAMK2A | TUBE1 | ITGA5 |
| MGAT4A | ACTR6 | NAGS |
| LMCD1 | SRI | IP6K3 |
| TRIP13 | NUP37 | ZG16B |
| MYO3B | ZNF638 | SCNN1D |
| SPEG | SLC25A40 | TDRD5 |
| LNX1 | SLC25A3 | ACP6 |
| ALDH3A2 | SART3 | PIGR |
| TFRC | UNG | CTLA4 |
| HMMR | PLXNA2 | ICOS |
| P4HA2 | SPAG5 | PCOLCE2 |
| ACADVL | TRAF4 | RNF123 |
| SIDT1 | ICAM3 | MAD2L1 |
| NDE1 | UBE2T | STARD4 |
| TMEM38A | EXOSC5 | F2RL2 |
| CYP2W1 | CST7 | PTTG1 |
| MCM2 | MCCC1 | SLC13A4 |
| PANX2 | GNB1 | OSR2 |
| CLCN4 | SAR1A | SKA3 |
| GSDMB | FDFT1 | BEND7 |
| PTGS2 | OPHN1 | QSOX2 |
| IGF2BP2 | KIF22 | LARGE2 |
| ST6GAL1 | PSEN1 | RAG1 |
| FRY | CPOX | TUB |
| GLI2 | CLDND1 | NEMP1 |
| ATP2A3 | HSP90AA1 | SCG5 |
| MGLL | IMPG2 | PLIN4 |
| NTN4 | HSPB11 | KLK1 |
| ARHGEF10L | SMARCD3 | TK1 |
| SEMA3A | MRPL22 | SPINDOC |
| GTSE1 | NFE2L1 | FADD |
| ACAT1 | LYRM2 | SCN9A |
| ADD2 | PLOD1 | CDK1 |
| FNDC3B | DIS3 | TMEM182 |
| FSCN1 | PIBF1 | ELOVL6 |
| SPAG5 | NUFIP1 | HOXB9 |
| ACACB | PPIE | HOPX |
| PAG1 | STARD7 | CCL11 |
| RAP1GAP | NOA1 | KLHL6 |
| NMRK2 | GSTP1 | CYSLTR1 |
| CTTNBP2 | FAM234B | CKS1B |
| RARB | EIF3I | OLR1 |
| UBE2T | EFR3B | SAA1 |
| PPP1R12B | CD82 | NUDT4 |
| CAPN6 | AK6 | SLC19A1 |
| ACTN2 | SEH1L | ATP2A2 |
| JADE1 | ABCB1 | SLC26A9 |
| LAMP3 | RRN3 | CD164L2 |
| FAP | CTTN | CHST2 |
| NEBL | MTIF2 | PCCA |
| ADCY2 | MGST2 | RMI2 |
| RBFOX1 | RAD54L | MSRA |
| ITM2A | EIF2AK1 | SPINK6 |
| MYH7B | TMED2 | DYNAP |
| BPIFB2 | ZFAND6 | C5orf46 |
| UBE2D4 | ZW10 | TRIML2 |
| OSBPL6 | TRIP6 | ARL14 |
| LXN | FTL | MB21D2 |
| SP140 | NLK | TIGIT |
| TNS1 | UIMC1 | KPNA2 |
| CEACAM1 | TXNDC16 | ZNF662 |
| DUSP13 | SF3B2 | NEB |
| LIPE | ERGIC2 | PMEL |
| PGM1 | AURKA | POU3F1 |
| PTPRH | PIR | TMPRSS11B |
| SESN1 | METTL2A | KIF18B |
| COL5A3 | DDX18 | LYRM7 |
| NDC80 | TPX2 | TNFRSF4 |
| CXCL2 | TGDS | MYBPC1 |
| MEF2C | COQ9 | AKR1C3 |
| CACNA1S | MAPKAPK5 | ADH1B |
| CADPS2 | SLC8B1 | H2AC11 |
| IL12RB2 | TASP1 | HOXC6 |
| SMARCD3 | RPLP0 | NUP62CL |
| FYB1 | CHGB | MB |
| STRADB | IGBP1 | RYR2 |
| EPB41L3 | DDX24 | F5 |
| BCKDHB | NECAP1 | RYR3 |
| PLOD1 | ANKRD24 | STYXL2 |
| FAT1 | SPTLC1 | VWA7 |
| COL16A1 | PAPOLA | ALG3 |
| NCOA1 | NUDC | ERVMER34-1 |
| KIF3C | MUL1 | AQP1 |
| FCN1 | SPG21 | CHCHD10 |
| PILRA | FLT3LG | HOXB7 |
| CTTN | PABPC4 | RHEX |
| ORC1 | CERS4 | MAGIX |
| MGST2 | KIF4A | FCGBP |
| RAD54L | RBM27 | H2BC9 |
| FOLH1 | LAMB4 |  |
| EPDR1 | SLC26A3 |  |
| SNX10 | DLD |  |
| CEACAM6 | FH |  |
| MYBPC2 | PITPNM3 |  |
| NOX4 | ORC6 |  |
| ACHE | TMEM101 |  |
| ADAMTS2 | CMA1 |  |
| TMPRSS11E | CEBPE |  |
| NID2 | OSGEP |  |
| PTHLH | G2E3 |  |
| AURKA | HECTD1 |  |
| SULT2B1 | HNRNPC |  |
| ASAP3 | SUPT16H |  |
| DNMT3B | GEMIN2 |  |
| TPX2 | WDR76 |  |
| DOCK3 | EZR |  |
| C3orf18 | CDC6 |  |
| TMEM40 | CDC23 |  |
| LZTS3 | AAAS |  |
| TESC | MSH2 |  |
| SIRPG | TMEM38B |  |
| OAS1 | PSMD5 |  |
| PXN | NUP188 |  |
| CHGB | NANS |  |
| PEBP1 | TBC1D2 |  |
| NOS1 | TLL2 |  |
| FXYD5 | TPSD1 |  |
| FXYD3 | MRPS18A |  |
| BIRC5 | HSP90AB1 |  |
| LAG3 | CDC7 |  |
| LYZ | ERMP1 |  |
| P3H2 | PSMD8 |  |
| EFNB1 | FBXL19 |  |
| KIF4A | MED15 |  |
| NAT14 | CECR2 |  |
| LAMB4 | BCL2L13 |  |
| LAMB1 | SEC14L2 |  |
| ITGA6 | PES1 |  |
| SMPX | SH3BP1 |  |
| TF | GCAT |  |
| ORC6 | CRYBB1 |  |
| ANGPT2 | ANKRD54 |  |
| MYH7 | DEPDC5 |  |
| SLC7A8 | CENPM |  |
| SLC22A17 | SLC5A4 |  |
| TGM1 | TOMM22 |  |
| WDR76 | RTCB |  |
| CAPN3 | BIK |  |
| TBX15 | MCAT |  |
| PHGDH | ASCC2 |  |
| CLSPN | SAMM50 |  |
| CDC45 | TXN2 |  |
| GABRP | EIF3D |  |
| KRT31 | CSF2RB |  |
| CDC6 | SLC25A17 |  |
| FMO2 | POLR3H |  |
| CRAT | FKBP3 |  |
| BLNK | CTSG |  |
| SORBS1 | PRMT5 |  |
| CRTAC1 | CDKN3 |  |
| BAMBI | VTI1B |  |
| IL11 | SNW1 |  |
| TREM2 | ITPK1 |  |
| CRISP3 | DHRS7 |  |
| ITPR3 | ASB2 |  |
| IL12RB1 | ERH |  |
| ACOT7 | PSMC1 |  |
| ABLIM1 | PPP4R3A |  |
| PALMD | YY1 |  |
| MAGEB2 | APEX1 |  |
| MISP | SRP54 |  |
| GADD45B | PRORP |  |
| PALM | DCAF11 |  |
| MMP11 | TM9SF1 |  |
| OSM | GMPR2 |  |
| SEC14L2 | CD40 |  |
| PLA2G3 | UQCC1 |  |
| LGALS1 | PRPF6 |  |
| SOX10 | PSMA7 |  |
| CENPM | MRGBP |  |
| SEPTIN3 | TCFL5 |  |
| SLC5A1 | TRIB3 |  |
| XBP1 | CSNK2A1 |  |
| MCM5 | SLC52A3 |  |
| RASD2 | CDS2 |  |
| CBX7 | PPP1R16B |  |
| SYNGR1 | TNNC2 |  |
| APOL1 | CELF4 |  |
| CHADL | ADNP2 |  |
| ACO2 | VAPA |  |
| CTSG | LPIN2 |  |
| GZMB | CSTF2 |  |
| PYGL | PSMD10 |  |
| CDKN3 | ATG4A |  |
| PLEK2 | PGRMC1 |  |
| GALNT16 | NXT2 |  |
| ASB2 | STAG2 |  |
| PAPLN | PLS3 |  |
| DHRS2 | EMD |  |
| MMP9 | PGK1 |  |
| PROCR | MAGT1 |  |
| GINS1 | HTATSF1 |  |
| MYBL2 | CD40LG |  |
| PABPC1L | PIN4 |  |
| NTSR1 | CENPI |  |
| MRGBP | DRP2 |  |
| SLC17A9 | NALCN |  |
| EEF1A2 | CDADC1 |  |
| CDC25B | DNAJC3 |  |
| ISM1 | SUPT20H |  |
| TRIB3 | MRPS31 |  |
| MYLK2 | VWA8 |  |
| FERMT1 | CLN5 |  |
| PLCB4 | OLFM4 |  |
| JAG1 | CORO1A |  |
| SNTA1 | PHKB |  |
| E2F1 | LONP2 |  |
| CST4 | CYB5B |  |
| WFDC2 | CCDC113 |  |
| SYNDIG1 | PSMD7 |  |
| TNNC2 | COG4 |  |
| MYOM1 | SLC7A6OS |  |
| LAMA1 | PLA2G15 |  |
| RNF125 | CMC2 |  |
| MXRA5 | AXIN1 |  |
| ATP1B4 | NPRL3 |  |
| CHRDL1 | STUB1 |  |
| ELF4 | UBE2I |  |
| GABRE | UBFD1 |  |
| SYTL4 | DNAJA3 |  |
| CENPI | BFAR |  |
| BEX4 | BCKDK |  |
| CAB39L | SYT17 |  |
| ACP5 | AAGAB |  |
| SGCG | IQCH |  |
| MEDAG | TRIP4 |  |
| OLFM4 | FAH |  |
| MSLN | RPAP1 |  |
| GDPD3 | VPS18 |  |
| PLLP | ZFAND1 |  |
| ZNF423 | SFRP1 |  |
| USB1 | PLAT |  |
| SLC38A7 | EIF3E |  |
| SMPD3 | NDRG1 |  |
| FA2H | ARMC1 |  |
| MLYCD | TRPS1 |  |
| HAGHL | SQLE |  |
| SLC7A5 | HNRNPL |  |
| RHBDL1 | PPP1R37 |  |
| MEFV | CKM |  |
| CRYM | PPP1R13L |  |
| IL21R | ERCC2 |  |
| TMC5 | ASF1B |  |
| SLC6A2 | POP4 |  |
| AQP9 | GPI |  |
| RASL12 | OVOL3 |  |
| IGDCC4 | CD79A |  |
| CD276 | CNFN |  |
| HOMER2 | KDELR1 |  |
| ZNF106 | BCAT2 |  |
| TGM5 | JAK3 |  |
| ZDHHC2 | RAB3A |  |
| CA2 | ISYNA1 |  |
| CALB1 | LSR |  |
| SFRP1 | HPN |  |
| JPH1 | GSK3A |  |
| CCN4 | GTPBP10 |  |
| NDRG1 | PMPCB |  |
| NCALD | BET1 |  |
| SH2D4A | DNAH11 |  |
| NEFM | MPP6 |  |
| MCM4 | ADAP1 |  |
| ASAH1 | CAV2 |  |
| KCNA7 | CAV1 |  |
| CKM | MET |  |
| DMPK | RNF32 |  |
| IL4I1 | HOXA1 |  |
| ASF1B | HOXA3 |  |
| TNNT1 | HIBADH |  |
| EPHX3 | ABHD11 |  |
| CCNE1 | GARS1 |  |
| DYRK1B | CHCHD2 |  |
| TJP3 | PDAP1 |  |
| APLP1 | CYP3A5 |  |
| TGFB1 | EIF3B |  |
| MYH14 | AIMP2 |  |
| ICAM5 | AGFG2 |  |
| CEACAM5 | LSM5 |  |
| CNFN | AP1S1 |  |
| GRIN2D | ANKMY2 |  |
| CLEC11A | CHCHD3 |  |
| PLA2G4C | COA1 |  |
| CAPS | RHEB |  |
| SCN1B | PRKAG2 |  |
| CDK6 | POLD2 |  |
| TFPI2 | BCL7B |  |
| PON3 | YKT6 |  |
| STEAP1B | EIF4H |  |
| PTN | PRUNE2 |  |
| GSDME | CORO2A |  |
| ATP6V0A4 | PTGR1 |  |
| CAV1 | CDC37L1 |  |
| MET | PLGRKT |  |
| WNT2 | KDM4C |  |
| HOXA1 | TESK1 |  |
| TSPAN12 | CA9 |  |
| CPED1 | NPDC1 |  |
| COBL | SHB |  |
| STX1A | UBE2R2 |  |
| EPHB6 | EXOSC3 |  |
| CYP3A5 | HPS1 |  |
| NUDT1 | ERLIN1 |  |
| TFR2 | DDX50 |  |
| IMPDH1 | MAPK8 |  |
| AGFG2 | SPOCK2 |  |
| SERPINE1 | MICU1 |  |
| PLOD3 | TWNK |  |
| AGR2 | SFXN3 |  |
| AEBP1 | NPM3 |  |
| SLC1A1 | CUEDC2 |  |
| FSD1L | BCCIP |  |
| PRUNE2 | STN1 |  |
| OGN | DKK1 |  |
| ASPN | XPNPEP1 |  |
| LHX6 | SMC3 |  |
| DNM1 | TFAM |  |
| AK1 | RPL28 |  |
| ELAVL2 | DNAJC12 |  |
| NCS1 | GIT1 |  |
| CA9 | RPL19 |  |
| TYRP1 | PSMD3 |  |
| MPDZ | RNF43 |  |
| DDX58 | RAD51C |  |
| NPDC1 | TRIM37 |  |
| GATA3 | PNPO |  |
| PHYH | CBX1 |  |
| CXCL12 | RECQL5 |  |
| ACTA2 | INTS2 |  |
| TLX1 | NUP88 |  |
| SFXN3 | C1QBP |  |
| CPEB3 | CCDC47 |  |
| NEURL1 | MED31 |  |
| DKK1 | FTSJ3 |  |
| UBE2S | SMARCD2 |  |
| CYP2C18 | UTP6 |  |
| KRT23 | PSMD11 |  |
| RUNDC3A | RAB5C |  |
| ENO3 | SGCA |  |
| RASD1 | LRRC59 |  |
| ALDH3A1 | ABCC3 |  |
| CCL2 | SMURF2 |  |
| COL1A1 | EFTUD2 |  |
| SGCA | YWHAE |  |
| ALOX12 | TMEM97 |  |
| CACNG1 | FOXN1 |  |
| HLF | UNC119 |  |
| SLC16A6 | ALDOC |  |
| MAP2K6 | RAB34 |  |
| MYH1 | OCIAD1 |  |
| CDR2L | LAMTOR3 |  |
| NMU | AREG |  |
| SOD3 | MANBA |  |
| TRIM2 | ZNF330 |  |
| SLC2A9 | GAR1 |  |
| NEIL3 | CLNK |  |
| KLHL5 | NCAPG |  |
| NCAPG | ELP4 |  |
| PPARGC1A | CCDC86 |  |
| CRYAB | PRPF19 |  |
| CTSC | CD5 |  |
| ZBTB16 | NDUFS8 |  |
| VWA5A | GTF2H1 |  |
| SIAE | SELPLG |  |
| FOLR1 | TSPAN11 |  |
| FOLR3 | ATP5F1B |  |
| GALNT18 | PTGES3 |  |
| KIAA1549L | BCL7A |  |
| SLC15A3 | LTA4H |  |
| EXPH5 | PARP11 |  |
| CYP27B1 | GPN3 |  |
| MYF6 | DUSP16 |  |
| TNS2 | GTF2H3 |  |
| GLI1 | EIF2B1 |  |
| PPM1H | RFC5 |  |
| FOXM1 | KRR1 |  |
| MYL2 | GAPDH |  |
| RAD51AP1 | COPS7A |  |
| MANSC1 | SUDS3 |  |
| ALDH2 | LDHB |  |
| SCNN1A | RAB35 |  |
| OAS3 | SRSF9 |  |
| OAS2 | KLRB1 |  |
| MGP | TDP2 |  |
| RASAL1 | FRK |  |
| ENDOU | SMIM8 |  |
| ADGRD1 | HDDC2 |  |
| TIMELESS | MTRF1L |  |
| CDCA3 | FANCE |  |
| CHPT1 | MRPL18 |  |
| ENO2 | IL17A |  |
| SLCO1B3 | MCM3 |  |
| COL12A1 | GLP1R |  |
| DSE | BAG2 |  |
| GCNT2 | CCNC |  |
| CEP85L | ALDH5A1 |  |
| ADTRP | RPS12 |  |
| HINT3 | GMNN |  |
| NCOA7 | SNX3 |  |
| SASH1 | ARFGEF3 |  |
| MDGA1 | GUCA1B |  |
| RBM24 | COX7A2 |  |
| CAP2 | GMDS |  |
| ALDH5A1 | VEGFA |  |
| GMNN | SLC29A1 |  |
| EYA4 | TENT5A |  |
| SOBP | PRSS16 |  |
| ARFGEF3 | HARS2 |  |
| EPM2A | SEMA5A |  |
| MDFI | BRD8 |  |
| SMOC2 | MRPS30 |  |
| PTK7 | HSPA9 |  |
| GMDS | PFDN1 |  |
| TTK | HBEGF |  |
| CCN6 | IK |  |
| CLIC5 | PCDHB7 |  |
| ENPP5 | CLINT1 |  |
| TBX18 | TTC1 |  |
| CEP72 | CCNG1 |  |
| C7 | BRIX1 |  |
| GHR | GNPDA1 |  |
| KIF20A | SKP1 |  |
| NNT | PPP2CA |  |
| HBEGF | C5orf15 |  |
| LOX | TRAPPC13 |  |
| SPARC | RARS1 |  |
| CCNG1 | ATP6V0E1 |  |
| IRX4 | BNIP1 |  |
| PDE4D | STC2 |  |
| PRLR | CNTN3 |  |
| LIFR | TBCCD1 |  |
| WWC1 | HRG |  |
| PDGFRB | PCCB |  |
| STC2 | CEP70 |  |
| CPEB4 | BCHE |  |
| SMC4 | PDCD10 |  |
| RBP1 | ECT2 |  |
| KAT2B | NPRL2 |  |
| BCHE | FXR1 |  |
| COL7A1 | NCBP2 |  |
| ECT2 | C3orf52 |  |
| CBLB | SLC41A3 |  |
| IQCG | CLCN2 |  |
| PLXNA1 | MOB1A |  |
| CISH | LMAN2L |  |
| MAPKAPK3 | TTL |  |
| ABCC5 | CCL20 |  |
| AADAC | FAHD2A |  |
| ABHD14B | NCL |  |
| ARHGEF26 | ACTR1B |  |
| DNAH1 | ZAP70 |  |
| ZBTB47 | ACTR3 |  |
| TNNC1 | EPB41L5 |  |
| ADAM23 | SF3B6 |  |
| IL1A | GPD2 |  |
| CCL20 | CENPA |  |
| TFCP2L1 | PSMD14 |  |
| CENPA | SNX17 |  |
| ITGB6 | MOGS |  |
| FNDC4 | GRB14 |  |
| REEP6 | AUP1 |  |
| IFIH1 | HTRA2 |  |
| LOXL3 | GALNT3 |  |
| EVA1A | MRPL19 |  |
| FN1 | FANCL |  |
| STAT1 | UNC50 |  |
| PECR | PDCL3 |  |
| IGFBP5 | UXS1 |  |
| EFHD1 | STK16 |  |
| GNLY | NOL10 |  |
| PRKAG3 | PLEKHB2 |  |
| SMYD1 | GORASP2 |  |
| IL1RL1 | DARS1 |  |
| SLC9A2 | PNO1 |  |
| MLPH | ORC4 |  |
| PROC | KISS1R |  |
| ODC1 | PHF13 |  |
| QPCT | PARK7 |  |
| SLC25A12 | ATP5PB |  |
| DLX2 | MAD2L2 |  |
| PLCL1 | KIAA2013 |  |
| KYNU | MFN2 |  |
| ARID3A | SMG7 |  |
| CD207 | GADD45A |  |
| PARD3B | UCHL5 |  |
| ANGPTL1 | BCAS2 |  |
| ELAPOR1 | CTH |  |
| RHOU | TFAP2E |  |
| FBXO2 | CD2 |  |
| LEPR | TMEM9 |  |
| NCF2 | HAO2 |  |
| RPE65 | GNPAT |  |
| AMPD1 | RIMS3 |  |
| TFAP2E | SDHB |  |
| OSCP1 | ACTL8 |  |
| NID1 | CTBS |  |
| BMP8B | KLHL12 |  |
| ACADM | GALE |  |
| PADI2 | CD46 |  |
| MFAP2 | MPL |  |
| RGS4 | FAAH |  |
| GBP1 | NSUN4 |  |
| P3H1 | MROH9 |  |
| SLC2A1 | PRDX6 |  |
| CDC20 | DARS2 |  |
| ARTN | SYF2 |  |
| TSPAN1 | NEK2 |  |
| SLC19A2 | RPS6KA1 |  |
| TNFSF4 | NSL1 |  |
| STMN1 | CENPF |  |
| MTFR1L | TXNDC12 |  |
| MAN1C1 | MESD |  |
| NEK2 | UBE3D |  |
| CENPF | CASQ2 |  |
| MTARC2 | SPP1 |  |
| MUC5B | CCNI |  |
| KIF14 | NDUFB3 |  |
| KLF7 | GTF3C3 |  |
| ATP10B | RAD23B |  |
| ELOVL4 | FKBP15 |  |
| FILIP1 | CTNNAL1 |  |
| SGIP1 | SET |  |
| PLAGL1 | CNTRL |  |
| AKAP7 | NEK6 |  |
| RAB32 | PPP6C |  |
| SLC16A7 | NDUFA8 |  |
| CASQ2 | RBM18 |  |
| SPP1 | HSDL2 |  |
| RARRES1 | INVS |  |
| PPL | GALNT12 |  |
| PCDH17 | ALG2 |  |
| KLF9 | FCF1 |  |
| BSPRY | NEK9 |  |
| NR4A3 | NPC2 |  |
| GALNT12 | ALDH6A1 |  |
| PGF | GPAM |  |
| BBOF1 | PYROXD2 |  |
| PPP4R4 | SMNDC1 |  |
| ALDH6A1 | TCTN3 |  |
| GPR68 | GOT1 |  |
| LGALSL | GNA13 |  |
| OGFRL1 | CAAP1 |  |
| IFIT3 | NUP43 |  |
| IFIT2 | PCMT1 |  |
| CUTC | CYSTM1 |  |
| PPP1R3C | PDZD11 |  |
| HELLS | ENY2 |  |
| GOT1 | EPC1 |  |
| DUSP1 | TAF12 |  |
| TEK | MTRF1 |  |
| MOB3B | DNAJC15 |  |
| CD274 | UFM1 |  |
| MTHFD1L | WBP4 |  |
| CYSTM1 | HSPH1 |  |
| CENPL | ALG5 |  |
| TNFSF18 | EXOSC8 |  |
| SMAD9 | SIL1 |  |
| TGFBI | PAIP2 |  |
| MYOT | PLS1 |  |
| EGR1 | TMPO |  |
| PLS1 | CLU |  |
| CLU | TNFRSF10B | |
| EPHX2 | TARDBP |  |
| TNFRSF8 | TNFRSF8 |  |
| NCAPH | COPS5 |  |
| LRAT | AKAP1 |  |
| TSHZ3 | COIL |  |
| ECHDC2 | SCPEP1 |  |
| ZSCAN18 | SPOP |  |
| PDZRN3 | SLC35B1 |  |
| CSTA | CAT |  |
| POPDC2 | TMEM39B |  |
| KIF18A | TMIGD3 |  |
| MAPK8IP1 | FLT3 |  |
| CRY2 | LAX1 |  |
| PILRB | KIAA1191 |  |
| GJA3 | OPN4 |  |
| ADGRB2 | HERPUD2 |  |
| FABP3 | CBX3 |  |
| TNFSF10 | HNRNPA2B1 | |
| CPXM2 | POLM |  |
| CSMD2 | SMU1 |  |
| UBL3 | SLC25A51 |  |
| PAEP | ACO1 |  |
| MYOG | SFTPA1 |  |
| LDB3 | PLAU |  |
| PTGFR | CISD1 |  |
| LRRC39 | ECD |  |
| WIPF3 | P4HA1 |  |
| INHBA | ZWINT |  |
| FKBP9 | VPS26A |  |
| RECK | DDX54 |  |
| PHF24 | SPRYD7 |  |
| CNTFR | EBPL |  |
| PLAU | CENPK |  |
| P4HA1 | CDK2 |  |
| ZWINT | NFE2 |  |
| CIT | MORF4L2 |  |
| ACADS | TTC21B |  |
| SSPN | EXOSC9 |  |
| ITPR2 | MXD4 |  |
| ATP7B | CKS2 |  |
| CENPK | DNPEP |  |
| ITIH5 | TTPAL |  |
| MMP19 | ATP5F1E |  |
| NR4A1 | GTSF1L |  |
| HOXC13 | ZNF831 |  |
| HOXC11 | CSE1L |  |
| STIL | ZBP1 |  |
| HJURP | VAMP7 |  |
| COL10A1 | PACSIN1 |  |
| ACVR1C | MRS2 |  |
| PLA2G12A | WRNIP1 |  |
| CKS2 | SNRPC |  |
| CHPF | H1-3 |  |
| PI3 | OARD1 |  |
| SLPI | AARS2 |  |
| KCNS1 | MAD2L1BP |  |
| TOX2 | KLHDC3 |  |
| EDN3 | KLHL31 |  |
| PTGIS | CPNE5 |  |
| CDH26 | DEK |  |
| PMEPA1 | EEF1E1 |  |
| BCAS4 | MYRF |  |
| ZBP1 | GOT2 |  |
| MAGEA10 | CLYBL |  |
| PAIP2B | EFNB2 |  |
| ATP8A1 | TM9SF2 |  |
| POF1B | UPF3B |  |
| H2BC11 | NDUFA1 |  |
| SPDEF | MRPS7 |  |
| APOBEC2 | GGA3 |  |
| TREM1 | SLC25A19 |  |
| KLHL31 | MBOAT7 |  |
| RAB17 | INSIG2 |  |
| CLYBL | POLR1B |  |
| HROB | GRIA3 |  |
| MYH2 | RPL23 |  |
| IL1B | EML2 |  |
| SLC25A23 | FOXA2 |  |
| TNFSF9 | PSMF1 |  |
| GRIA3 | DSTN |  |
| CD70 | S1PR4 |  |
| C3 | HNRNPR |  |
| TRIP10 | ARMCX5 |  |
| FOSB | ERGIC3 |  |
| RTN2 | ROMO1 |  |
| FLRT3 | TUBGCP3 |  |
| BFSP1 | PCID2 |  |
| LAMP5 | PDCD2L |  |
| BPIFB1 | UBA2 |  |
| AMOT | CCR7 |  |
| XRCC3 | FLRT1 |  |
| MCF2L | BECN1 |  |
| SLURP1 | TRAP1 |  |
| KRT36 | KTN1 |  |
| BCL2L12 | SIX1 |  |
| TSKS | RHOT1 |  |
| STATH | FAM78A |  |
| HTN1 | TIMM8A |  |
| IFI6 | INTS11 |  |
| SIX1 | RGS13 |  |
| DLGAP5 | FGD3 |  |
| L3HYPDH | COX7C |  |
| AIF1L | TRAF2 |  |
| ATP13A4 | MASP1 |  |
| BEST3 | BEST3 |  |
| AUNIP | RAB3IP |  |
| ADGRE2 | YEATS4 |  |
| KLF2 | CRYGN |  |
| F2RL3 | AUNIP |  |
| SYNGR3 | ECHS1 |  |
| PKMYT1 | SEM1 |  |
| FBXL16 | POR |  |
| CHTF18 | PPAT |  |
| TNFRSF19 | ATF4 |  |
| ZFP36 | LIF |  |
| ASPHD2 | EMC4 |  |
| BAIAP2L2 | POT1 |  |
| APOL2 | LSM8 |  |
| RAC2 | CDHR3 |  |
| RIBC2 | NDUFA5 |  |
| KRT17 | MRPS12 |  |
| FLNC | MTX2 |  |
| CALU | CCDC32 |  |
| LRRC17 | IVD |  |
| MYO1B | KNSTRN |  |
| CHN1 | THAP10 |  |
| HOXD9 | ANAPC13 |  |
| HOXD10 | PSMA1 |  |
| HOXD11 | SERGEF |  |
| HOXD13 | PIMREG |  |
| MYO5C | RPAIN |  |
| CGNL1 | ADCY4 |  |
| KNSTRN | PARP2 |  |
| CSRP3 | SNX6 |  |
| PIMREG | EGLN3 |  |
| KIF1C | DAD1 |  |
| KLK14 | REEP5 |  |
| AJUBA | SEC14L1 |  |
| FOXA1 | SGO1 |  |
| EGLN3 | PRKCSH |  |
| EPB41L4A | TOMM40 |  |
| FGF13 | RTN4IP1 |  |
| SGO1 | MAS1 |  |
| CDH15 | NDUFA10 |  |
| SHC2 | GDF15 |  |
| GAMT | TNNI2 |  |
| CRACR2A | PAK4 |  |
| GDPD2 | MNX1 |  |
| CNN1 | CEP85 |  |
| APOC1 | EXOSC2 |  |
| GADD45G | UBE2M |  |
| ATP8B3 | EIF2S3 |  |
| BST2 | LRRC47 |  |
| SLC27A1 | ZNF426 |  |
| COLGALT1 | PNCK |  |
| CACNG6 | DKC1 |  |
| FCHO1 | DUSP9 |  |
| KLHDC7B | C12orf65 |  |
| PXDN | NOL11 |  |
| PGPEP1 | UBA1 |  |
| HRC | PPIL4 |  |
| HELZ2 | AAR2 |  |
| TNNT3 | ATP6V1E1 |  |
| TNNI2 | EMC8 |  |
| COL5A1 | COX7B |  |
| CYP2E1 | PPT1 |  |
| PNPLA7 | ABCB7 |  |
| RBBP8NL | GFPT2 |  |
| SHFL | TUBG1 |  |
| PNCK | PSME3 |  |
| DUSP9 | PSMC3IP |  |
| ZNF331 | VPS25 |  |
| ULBP2 | NDUFA2 |  |
| SYNE1 | NDFIP1 |  |
| ULBP3 | UBE2D2 |  |
| EPS8L1 | PPFIA1 |  |
| BPIFA2 | NINJ1 |  |
| ACSS2 | ZCCHC9 |  |
| C1QL1 | STARD3 |  |
| GINS2 | RARA |  |
| IDO1 | PRKAB2 |  |
| HAUS8 | PDHA1 |  |
| SH3BP5 | MCCC2 |  |
| PSMC3IP | RHPN2 |  |
| AOC3 | LRRC9 |  |
| ANO1 | ACTR10 |  |
| PPFIA1 | LRRC41 |  |
| KREMEN2 | PPARG |  |
| CKMT2 | HSD17B7 |  |
| KRT34 | TIMM10B |  |
| TOP2A | PTCD3 |  |
| PPP1R1B | IMMT |  |
| KHDRBS3 | MRPL35 |  |
| RAI2 | RAN |  |
| ZNF132 | MYBBP1A |  |
| FAAP24 | COQ3 |  |
| PODNL1 | SEC61G |  |
| MATN3 | GRSF1 |  |
| SPATA6 | UTP3 |  |
| SLC6A11 | ITGB4 |  |
| PPARG | WBP2 |  |
| PER2 | CLEC10A |  |
| SEC61G | RIDA |  |
| JCHAIN | PCBD2 |  |
| ITGB4 | ERAL1 |  |
| CLEC10A | NIP7 |  |
| SLC52A1 | TERF2 |  |
| XAF1 | DAP3 |  |
| PCBD2 | CRP |  |
| RAB25 | FCRL2 |  |
| TESMIN | SERINC3 |  |
| ZBED3 | ANGPTL3 |  |
| KANK4 | POMP |  |
| CHRM3 | SLC41A1 |  |
| MYH8 | CCNA1 |  |
| MYH10 | GPALPP1 |  |
| CHIT1 | KL |  |
| LGR6 | RFC3 |  |
| DCLK1 | BEX2 |  |
| CCNA1 | FAM104A |  |
| EPSTI1 | HSPBP1 |  |
| POSTN | MORC2 |  |
| TPT1 | GIMAP6 |  |
| EPHB2 | AGAP3 |  |
| MACROD1 | LARS1 |  |
| MYH11 | CCDC59 |  |
| MYO18B | RRAS2 |  |
| C1QTNF6 | ERG28 |  |
| NTS | EIF2S1 |  |
| SPINK5 | MRPS36 |  |
| LYVE1 | BHLHE40 |  |
| MICAL2 | DPH6 |  |
| DUSP26 | EMC7 |  |
| DGLUCY | LAMTOR5 |  |
| TTC9 | AP4B1 |  |
| LOXL2 | PPHLN1 |  |
| PEBP4 | ARF3 |  |
| ADAMDEC1 | IAH1 |  |
| CCNB1 | SAA2 |  |
| CHL1 | RPS15A |  |
| MEIS2 | RBM17 |  |
| PSRC1 | CCNH |  |
| SORT1 | RBMX2 |  |
| WNT2B | STK26 |  |
| PTGFRN | YARS1 |  |
| VTCN1 | GNL2 |  |
| NGF | TIMM10 |  |
| RSAD2 | TMEM258 |  |
| LPIN1 | DZIP1 |  |
| CMPK2 | UBAC2 |  |
| SAA2 | TPP2 |  |
| IL6ST | CARS2 |  |
| FST | OSTF1 |  |
| NAV1 | UBQLN1 |  |
| IL2RA | AGTPBP1 |  |
| ECHDC3 | ISCA1 |  |
| CABLES1 | TMEM243 |  |
| EMP1 | HILPDA |  |
| RERG | SNX14 |  |
| PRH2 | NAT10 |  |
| SPOCD1 | TESPA1 |  |
| CDCA8 | CDK4 |  |
| CYP2J2 | TROAP |  |
| DSC1 | ZC3H10 |  |
| DTNA | HNRNPA1 |  |
| DAGLA | OS9 |  |
| TCN1 | CD164 |  |
| COL4A2 | AFG1L |  |
| CLDN10 | KCNMB4 |  |
| POGLUT2 | NTPCR |  |
| NREP | MFSD9 |  |
| ANXA1 | MRPS9 |  |
| FAM189A2 | USP44 |  |
| ADAM19 | PWP1 |  |
| SDS | BORA |  |
| MSI1 | RCBTB1 |  |
| TBX3 | MED4 |  |
| OASL | NUDT15 |  |
| CD36 | SETDB2 |  |
| KCP | GPNMB |  |
| ANKRD6 | NUP42 |  |
| NT5E | ZDHHC4 |  |
| LCA5 | BZW2 |  |
| LMO2 | TBRG4 |  |
| EHF | DDX56 |  |
| ELF5 | TTC5 |  |
| ITGA7 | NKX2-8 |  |
| PPP1R1A | NMT1 |  |
| TROAP | TACO1 |  |
| B4GALNT1 | DCAF7 |  |
| COQ10A | NDUFB5 |  |
| KRT7 | MRPL47 |  |
| SMPD2 | BLK |  |
| TEC | SAP130 |  |
| PRADC1 | IMP4 |  |
| SEMA4F | LRRC8A |  |
| AGT | ECPAS |  |
| GLUL | ST6GALNAC4 | |
| NIBAN1 | STXBP1 |  |
| SP110 | CDK5RAP2 |  |
| SERPINE2 | PRPF4 |  |
| WNT10A | TEX10 |  |
| CYP27A1 | MRPL50 |  |
| CKAP2 | NCBP1 |  |
| THSD1 | ANP32B |  |
| BORA | PDCL |  |
| LMO7 | POLR1E |  |
| SCEL | PLAA |  |
| SPRY2 | APTX |  |
| EDNRB | RNF38 |  |
| SCRN1 | SIT1 |  |
| IGF2BP3 | ALDH1B1 |  |
| GPNMB | ARHGEF39 |  |
| IL6 | TMEM14B |  |
| CCM2 | IRF4 |  |
| MYO1G | TUBB2A |  |
| TTYH3 | HMGA1 |  |
| ADAMTS7 | TAF8 |  |
| ALPK3 | ARRB1 |  |
| RTP4 | TTPA |  |
| GALNT5 | UNC13C |  |
| SKIL | SLTM |  |
| IL1RN | KNL1 |  |
| IL36A | RTF1 |  |
| BIN1 | RPLP1 |  |
| GYPC | RMDN3 |  |
| KLF4 | ITPKA |  |
| RALGPS1 | CYP19A1 |  |
| TMOD1 | RSL24D1 |  |
| KIF12 | BCAR3 |  |
| CTSV | SELENOI |  |
| DSCC1 | HADHB |  |
| IL33 | KHK |  |
| IL11RA | THUMPD2 |  |
| TMEM8B | PREB |  |
| ARHGEF39 | LRPPRC |  |
| GMPR | ATAD1 |  |
| FOXF2 | BTBD16 |  |
| TCF19 | KIF11 |  |
| NRM | ARL3 |  |
| MYO7A | CEP55 |  |
| IL18BP | RBP4 |  |
| SYTL2 | FAM149B1 |  |
| PI15 | ASCC1 |  |
| SLCO5A1 | RPS24 |  |
| SULF1 | METTL5 |  |
| DDX60 | SSB |  |
| TRPC6 | IDH1 |  |
| MMP7 | OLA1 |  |
| DCUN1D5 | WDR12 |  |
| POU2F3 | ABI2 |  |
| ARHGAP20 | COX17 |  |
| MMP13 | AP1AR |  |
| KIF23 | HNRNPD |  |
| ITGA11 | MMRN1 |  |
| KNL1 | TRPC3 |  |
| ITPKA | PPA2 |  |
| STRA6 | CENPE |  |
| GCOM1 | EGF |  |
| IFI44L | PAPSS1 |  |
| GIPC2 | GUCD1 |  |
| IFI44 | ERP27 |  |
| CGREF1 | GABARAPL1 | |
| HADHB | CPNE8 |  |
| CENPO | YARS2 |  |
| TRIM54 | ZCRB1 |  |
| CH25H | CD27 |  |
| KIF11 | GAS2L3 |  |
| TACC2 | SLC15A4 |  |
| DUSP5 | TDG |  |
| CEP55 | MMAB |  |
| ADAMTS14 | BRCA2 |  |
| DNA2 | CERS5 |  |
| MYPN | ITGB7 |  |
| AOX1 | TMBIM6 |  |
| ITGAV | ESD |  |
| SECISBP2L | DENR |  |
| CILP | RBM26 |  |
| SEMA7A | ZIC5 |  |
| FAM13A | RAB20 |  |
| HERC5 | GRTP1 |  |
| ZGRF1 | CUL4A |  |
| GPAT3 | FRMD6 |  |
| BMPR1B | ARMH4 |  |
| MMRN1 | NAA30 |  |
| PDE5A | WDR89 |  |
| NAAA | EFCAB11 |  |
| CXCL9 | JDP2 |  |
| FRAS1 | MFAP1 |  |
| SHROOM3 | LYSMD2 |  |
| CENPE | HDC |  |
| HADH | BNIP2 |  |
| EGF | GTF2A2 |  |
| SLC39A8 | SRP14 |  |
| FBN2 | CDAN1 |  |
| SHISAL1 | ANP32A |  |
| PIK3C2G | TSPAN3 |  |
| AMIGO2 | BBS4 |  |
| COL2A1 | RHCG |  |
| LUM | SEC11A |  |
| SLC46A3 | RUSF1 |  |
| SUOX | UQCRC2 |  |
| RDH16 | MARVELD3 |  |
| BRCA2 | NUDT7 |  |
| GALNT6 | RPS2 |  |
| DIAPH3 | NDUFB10 |  |
| ZIC5 | UTP4 |  |
| CDH24 | NOB1 |  |
| FITM1 | UNC45B |  |
| RDH12 | PCTP |  |
| STON2 | TOM1L1 |  |
| FBLN5 | SCRN2 |  |
| WARS1 | AFG3L2 |  |
| DUOXA2 | ARHGDIA |  |
| SLC27A2 | EIF4A3 |  |
| FGF7 | ANAPC11 |  |
| GCNT3 | TBCD |  |
| DISP2 | FN3KRP |  |
| TPM1 | CEP131 |  |
| PIF1 | MIEN1 |  |
| RHCG | SAMD1 |  |
| FANCI | PFKL |  |
| TICRR | HUNK |  |
| ST8SIA2 | SOD1 |  |
| SH3GL3 | CACNG8 |  |
| ITGAX | EFHD2 |  |
| MYLK3 | PEX14 |  |
| NLRC5 | RPL11 |  |
| CDH11 | GPN2 |  |
| RPL3L | BCL10 |  |
| GALNS | TINAGL1 |  |
| UNC45B | TIPRL |  |
| ARSG | CREG1 |  |
| ABCA8 | TMCO1 |  |
| PRELID3A | UFC1 |  |
| SLC13A5 | NUF2 |  |
| SLC16A3 | SDHC |  |
| ZNF750 | MRPL24 |  |
| TNFRSF11A | XPR1 |  |
| P3H4 | SF3B4 |  |
| FKBP10 | TARS2 |  |
| MISP3 | ANP32E |  |
| NFIC | MRPL9 |  |
| FEM1A | SYT14 |  |
| IFITM3 | EIF2D |  |
| PGGHG | INTS7 |  |
| COL6A1 | TAF1A |  |
| COL6A2 | S100A8 |  |
| TRPM2 | HAX1 |  |
| DOP1B | C1orf43 |  |
| EMP3 | ILF2 |  |
| SLC47A1 | SCCPDH |  |
| RCN3 | MAP3K21 |  |
| SLC2A5 | ACP1 |  |
| PADI3 | SRP9 |  |
| FHAD1 | NVL |  |
| PADI1 | ARF1 |  |
| MYOM3 | CNIH4 |  |
| DMRTA2 | PYCR2 |  |
| PLK4 | SOX13 |  |
| TINAGL1 | PDIA6 |  |
| KIF2C | CALM2 |  |
| CYP4B1 | CHAC2 |  |
| SYPL2 | CIAO1 |  |
| SLC44A3 | MRPS5 |  |
| GPR161 | TEX261 |  |
| ALDH9A1 | RALB |  |
| DPT | TMEM177 |  |
| FCGR2A | LIPT1 |  |
| NUF2 | AFF3 |  |
| CASQ1 | POLR2D |  |
| ABL2 | GALNT13 |  |
| XPR1 | SCRN3 |  |
| RGS16 | TMEFF2 |  |
| RORC | HSPD1 |  |
| ECM1 | SLC25A38 |  |
| CGN | NXPE3 |  |
| MINDY1 | EIF2A |  |
| ANXA9 | FIP1L1 |  |
| SELENBP1 | EPHA5 |  |
| SEMA6C | ENOPH1 |  |
| HORMAD1 | TBCK |  |
| DTL | CISD2 |  |
| SUSD4 | CCNA2 |  |
| FLG2 | METTL14 |  |
| CRNN | SETD7 |  |
| S100A8 | RPS3A |  |
| TPM3 | SKP2 |  |
| CREB3L4 | GIN1 |  |
| FLG | COMMD10 |  |
| ACTA1 | ATG12 |  |
| GALNT2 | CXCL14 |  |
| ETNK2 | PCYOX1L |  |
| PPFIA4 | BOD1 |  |
| PLEKHA6 | ZMAT2 |  |
| OSR1 | RPL7L1 |  |
| ATP6V1C2 | TPBG |  |
| MEIS1 | RNF217 |  |
| NPHP1 | ARHGAP18 |  |
| SLC20A1 | MTFR2 |  |
| DLX1 | AIG1 |  |
| GULP1 | TMEM181 |  |
| FAM171B | C7orf50 |  |
| SPAG16 | CCZ1B |  |
| ACKR3 | C7orf26 |  |
| RBMS3 | EGFR |  |
| CSRNP1 | MDH2 |  |
| ITGA9 | NIPSNAP2 |  |
| CAND2 | PSPH |  |
| IL17RD | ZAN |  |
| LRIG1 | TLK2 |  |
| COL8A1 | NOM1 |  |
| PHLDB2 | GPR174 |  |
| BOC | EBP |  |
| ALDH1L1 | SNX12 |  |
| TMEM44 | IL2RG |  |
| UCN2 | DNAJC5B |  |
| TM4SF19 | LACTB2 |  |
| MUC4 | TERF1 |  |
| OCIAD2 | MTDH |  |
| PLAC8 | EBAG9 |  |
| DDIT4L | UTP23 |  |
| ANK2 | TATDN1 |  |
| TIFA | HAUS6 |  |
| CCNA2 | C9orf72 |  |
| NKD2 | ZCCHC7 |  |
| MYO10 | SIGMAR1 |  |
| GZMA | MFSD14B |  |
| PIK3R1 | INIP |  |
| HAPLN1 | POLE3 |  |
| SSBP2 | SURF4 |  |
| IQGAP2 | REXO4 |  |
| FBXL17 | NTMT1 |  |
| CXCL14 | LRSAM1 |  |
| SPINK7 | PROSER2 |  |
| N4BP3 | A1CF |  |
| RMND5B | GLUD1 |  |
| TENM2 | FRA10AC1 |  |
| KCNMB1 | INA |  |
| MYLK4 | TAF5 |  |
| PLA2G7 | PPRC1 |  |
| PPP1R18 | ADM |  |
| PRIM2 | SLC5A12 |  |
| MLIP | IMMP1L |  |
| SCUBE3 | HSD17B12 |  |
| TPBG | EIF3M |  |
| IRAK1BP1 | RPS3 |  |
| MMS22L | NCAM1 |  |
| MTFR2 | AASDHPPT |  |
| PNLDC1 | ADAM33 |  |
| GNA12 | MS4A2 |  |
| SDK1 | TMEM25 |  |
| EGFR | CNBD2 |  |
| CDCA5 | FAU |  |
| IGFBP3 | PPP4C |  |
| NCAPG2 | ALDOA |  |
| NLGN4X | HIRIP3 |  |
| TMEM47 | TMEM219 |  |
| SYTL5 | CNTN5 |  |
| MSN | SAP18 |  |
| CHST7 | ADGRL3 |  |
| MFHAS1 | LYPD1 |  |
| MAGEA4 | LYPD6B |  |
| ZNF185 | GPM6A |  |
| RGS20 | CNDP1 |  |
| GINS4 | CCT5 |  |
| TRIM55 | RILPL2 |  |
| SYBU | DCP1B |  |
| LRP12 | OXSM |  |
| FAM83A | IPMK |  |
| SLC39A4 | TEX30 |  |
| NFIB | MBIP |  |
| PLIN2 | EXT2 |  |
| CDKN2A | KIN |  |
| WDR31 | PIGF |  |
| SLC25A25 | ACSL1 |  |
| LCN2 | SLC25A4 |  |
| HMCN2 | CCDC122 |  |
| CACNA1B | BAG3 |  |
| FAM171A1 | MZT2B |  |
| FAM13C | OBI1 |  |
| ANKRD1 | ATP5F1A |  |
| HTR7 | SPC25 |  |
| PLEKHS1 | POC5 |  |
| MKI67 | FAM151B |  |
| ADAM12 | PAN3 |  |
| SYT8 | PFKM |  |
| SLC43A1 | GPD1L |  |
| KLHL35 | SLC30A6 |  |
| SERPINH1 | PELO |  |
| CAPN5 | TCTEX1D1 |  |
| P4HA3 | PANK1 |  |
| ADAM33 | GGPS1 |  |
| FADS1 | MED21 |  |
| CHEK1 | PLOD2 |  |
| FEZ1 | CWC27 |  |
| JPH2 | CENPH |  |
| DSN1 | SCOC |  |
| LTO1 | CETN3 |  |
| TM7SF2 | HNRNPU |  |
| HMGA2 | RANBP2 |  |
| MMP3 | PTPRR |  |
| MPP7 | SCN3A |  |
| FAM124A | CFDP1 |  |
| HNMT | KCNJ16 |  |
| PDCD4 | DGKE |  |
| ADRA2A | GDPD1 |  |
| VEGFC | NUS1 |  |
| PRSS23 | GRAP |  |
| ITPR1 | SLC5A10 |  |
| SLC7A11 | CABYR |  |
| GPR158 | ANKRD29 |  |
| THRB | GPR15 |  |
| MAGI1 | CEP112 |  |
| AKAP6 | OBSCN |  |
| ME3 | TRIM11 |  |
| ADAMTS12 | BUB3 |  |
| ACAD8 | ATP5MC3 |  |
| NR3C2 | TMSB4Y |  |
| AKR1C2 | CHODL |  |
| ADAM8 | MRPL39 |  |
| TMEM45B | ATP5PF |  |
| ACSL1 | SLFN13 |  |
| SLC25A4 | EME1 |  |
| BICD1 | AZIN1 |  |
| ZNF385D | ATP6V1C1 |  |
| TDO2 | MMS19 |  |
| CCL28 | ZCCHC10 |  |
| GFRA1 | C16orf87 |  |
| BEND6 | HEATR3 |  |
| TLCD4 | NIFK |  |
| HSPB8 | OXA1L |  |
| ATP5F1A | LARP1 |  |
| SPC25 | CNOT8 |  |
| SPOCK1 | VSIG4 |  |
| PFKM | RRAGA |  |
| IGSF10 | RMND1 |  |
| SPARCL1 | VBP1 |  |
| GPD1L | ADK |  |
| GJA1 | BATF |  |
| ANKRD22 | WHAMM |  |
| PANK1 | RWDD2B |  |
| PLOD2 | CCT8 |  |
| NR4A2 | SCAF4 |  |
| ADGRF1 | CDK20 |  |
| LPCAT1 | PCGF6 |  |
| PLEKHG4B | ATP5MPL |  |
| ADPRHL1 | RPL30 |  |
| PTPRD | FAM122B |  |
| TMPRSS11D | HK1 |  |
| LGI4 | TYSND1 |  |
| DDAH1 | PHF6 |  |
| CACNA2D1 | NPTN |  |
| CABYR | SAMD8 |  |
| CHST9 | AIFM1 |  |
| THY1 | MS4A1 |  |
| C16orf74 | FBRS |  |
| RETREG1 | FRRS1 |  |
| ABI3BP | MALSU1 |  |
| ANGPT1 | EIF4A2 |  |
| ABCA6 | SST |  |
| C4orf19 | ODR4 |  |
| UCHL1 | NECAP2 |  |
| PGM5 | TMED6 |  |
| OBSCN | CCNB2 |  |
| LONRF1 | PWWP3B |  |
| PPP1R3A | TMEM164 |  |
| GBP5 | PSMG3 |  |
| DIPK1A | C12orf43 |  |
| PDLIM3 | RER1 |  |
| SORBS2 | WIPI2 |  |
| L3MBTL4 | BABAM2 |  |
| JAM2 | PTPDC1 |  |
| ADAMTS1 | PRXL2C |  |
| WNT7A | CNNM4 |  |
| PLCL2 | TENT5B |  |
| SKA1 | CDC25C |  |
| PIEZO2 | CNOT11 |  |
| EME1 | CPA2 |  |
| VOPP1 | TSR2 |  |
| CYP2U1 | ZC3H18 |  |
| AK9 | NIT1 |  |
| PTPRN2 | EDA |  |
| MARVELD1 | MPZ |  |
| GOLGA7B | RAPGEF6 |  |
| GRAMD2B | MIS18A |  |
| SLC16A1 | PAXBP1 |  |
| PIK3AP1 | MRPL10 |  |
| TTN | DONSON |  |
| SPAG17 | CSRP1 |  |
| DEPTOR | ATP5MC1 |  |
| SLC26A2 | CHAF1B |  |
| WIF1 | PSMB4 |  |
| MMP16 | TGM7 |  |
| KCNMA1 | TMEM69 |  |
| ADAMTSL3 | TPPP3 |  |
| CXCL13 | CALM3 |  |
| MAP3K7CL | DFFA |  |
| CLDN17 | CCDC58 |  |
| CLDN8 | UBASH3A |  |
| FUT6 | NDUFV3 |  |
| PCDH1 | PKNOX1 |  |
| HKDC1 | RRP1B |  |
| CD109 | G6PD |  |
| UBE2L6 | AGPAT3 |  |
| RAB11FIP1 | CFAP410 |  |
| GLYATL2 | FAM207A |  |
| TBC1D31 | LSS |  |
| ATAD2 | C21orf58 |  |
| COX6A2 | PCNT |  |
| B3GNT7 | ZNF761 |  |
| MPV17L | SPTBN4 |  |
| BUB1B | JAML |  |
| RBPMS | UBE2Q1 |  |
| KLHL40 | FDPS |  |
| NRG1 | FGFR4 |  |
| LRP8 | ZNF394 |  |
| MMP14 | TONSL |  |
| IL34 | SQSTM1 |  |
| KIT | PSMC2 |  |
| CACNA2D3 | FDXR |  |
| CCNB2 | SAP30BP |  |
| APPL1 | ITGA5 |  |
| PWWP3B | ZNF385A |  |
| MX1 | NAGS |  |
| SLC34A2 | LSM12 |  |
| ACAN | EMC10 |  |
| TRIM63 | LARP4 |  |
| CFAP251 | KRT82 |  |
| GRHL3 | IP6K3 |  |
| TENT5B | TREML1 |  |
| GPR153 | POLR3K |  |
| GPRASP2 | TBC1D24 |  |
| H2BC5 | ZG16B |  |
| CDC25C | CLPB |  |
| H4C8 | UBXN1 |  |
| CD1C | LRP5 |  |
| CD1E | LEXM |  |
| SLAMF8 | AK4 |  |
| PLA2G2F | AKR7A3 |  |
| PINK1 | RBBP4 |  |
| MPZ | TMCO4 |  |
| STC1 | SCNN1D |  |
| TNNI1 | OMA1 |  |
| CLIC6 | FUBP1 |  |
| IGF2BP1 | ATF3 |  |
| SIM2 | TDRD5 |  |
| BTG2 | BPNT1 |  |
| TCHH | ACP6 |  |
| SPRR2G | PIGR |  |
| SPON2 | DPY30 |  |
| PIP | MAIP1 |  |
| GNE | WDCP |  |
| FNDC5 | IWS1 |  |
| CPAMD8 | TSACC |  |
| KALRN | TMEM79 |  |
| CILP2 | RETNLB |  |
| TFF3 | CTLA4 |  |
| TMPRSS3 | ICOS |  |
| SLC37A1 | GTPBP8 |  |
| CSTB | GMPS |  |
| LRRC3 | TIPARP |  |
| VAV2 | RPL9 |  |
| S100B | PCOLCE2 |  |
| SLC2A6 | U2SURP |  |
| COX6B2 | CPA3 |  |
| PLPP7 | KIAA1143 |  |
| MYL3 | WDR43 |  |
| AZGP1 | CLEC3B |  |
| FGFR4 | RTP3 |  |
| HK3 | POLR2H |  |
| LY6K | RPN1 |  |
| LY6E | SENP2 |  |
| TONSL | MELTF |  |
| RECQL4 | AIMP1 |  |
| SCGB3A1 | H2AZ1 |  |
| COX7A1 | PGRMC2 |  |
| TMEM143 | RNF123 |  |
| ITGA5 | ABHD18 |  |
| MPP3 | DUSP7 |  |
| NAGS | PRSS12 |  |
| DBF4B | HMGB2 |  |
| FMNL3 | SAP30 |  |
| AQP5 | MAD2L1 |  |
| RACGAP1 | ANXA5 |  |
| KRT84 | CEP44 |  |
| SPC24 | TMEM144 |  |
| IP6K3 | ANAPC10 |  |
| TNFSF13 | ABCE1 |  |
| TEDC2 | LSM6 |  |
| CCNF | MOCS2 |  |
| BICDL2 | NDUFAF2 |  |
| ZG16B | STARD4 |  |
| TPCN2 | PGGT1B |  |
| ZYG11B | F2RL2 |  |
| PLPP3 | AGGF1 |  |
| PRKAA2 | WDR41 |  |
| RBP7 | SPINK1 |  |
| FBLIM1 | CASP3 |  |
| SLC25A34 | UTP15 |  |
| PDPN | GFM2 |  |
| SYNC | UQCRQ |  |
| UBXN10 | GJB7 |  |
| SCNN1D | SFXN1 |  |
| NFIA | KIAA0895 |  |
| NEXN | RPS14 |  |
| HENMT1 | PTTG1 |  |
| GFI1 | CAMLG |  |
| AGL | KIF6 |  |
| ZNF281 | SHH |  |
| OLFML2B | SLC13A4 |  |
| TDRD5 | BRI3 |  |
| SNED1 | HNF4G |  |
| C1orf115 | MED30 |  |
| ACP6 | TNFRSF11B | |
| KIF26B | DNAAF5 |  |
| PKDCC | SUN1 |  |
| HAAO | INTS1 |  |
| IL24 | OSR2 |  |
| PIGR | SNAPC3 |  |
| CAPN13 | UBAP1 |  |
| FRZB | NFIL3 |  |
| FBXO41 | GKAP1 |  |
| ACTG2 | C9orf64 |  |
| SLC16A14 | HNRNPK |  |
| EN1 | STRBP |  |
| SPATA18 | GAPVD1 |  |
| NOSTRIN | NDUFB6 |  |
| XIRP2 | STOML2 |  |
| NEURL3 | SLITRK5 |  |
| BNIPL | FBXO33 |  |
| TMOD4 | SPTSSA |  |
| SPRR3 | SUGT1 |  |
| SPRR2D | SKA3 |  |
| S100A9 | MICU2 |  |
| ANTXR2 | KBTBD6 |  |
| DAPL1 | BEND7 |  |
| COL6A3 | PRPF18 |  |
| LMOD3 | SLC18A2 |  |
| SLC15A2 | QSOX2 |  |
| EIF4E3 | FAM204A |  |
| LMOD1 | PRDX3 |  |
| ELF3 | GHITM |  |
| CXCR1 | HPRT1 |  |
| CIP2A | ZMYND19 |  |
| SGO2 | STOX1 |  |
| AIM2 | BMS1 |  |
| EIF5A2 | ISCA2 |  |
| PPM1L | LARGE2 |  |
| CTLA4 | TTC7B |  |
| ICOS | NELL1 |  |
| ADAMTS9 | HACD1 |  |
| PPM1K | NOLC1 |  |
| PTX3 | COPS2 |  |
| DNASE1L3 | CCT2 |  |
| RBM47 | PCBD1 |  |
| APBB2 | ARIH1 |  |
| PRRT3 | COX11 |  |
| PCOLCE2 | ANAPC16 |  |
| FYCO1 | TPP1 |  |
| LRRC2 | RAG1 |  |
| FBXO40 | TUB |  |
| DTX3L | PRKCB |  |
| YEATS2 | COG1 |  |
| DNALI1 | SLFN5 |  |
| CAMK2N2 | PPIB |  |
| HEYL | CIAO2A |  |
| RFC4 | PEX11A |  |
| RPL39L | CLPX |  |
| ABLIM2 | NEMP1 |  |
| EMCN | NAB2 |  |
| SPRY1 | STAT6 |  |
| RNF123 | MRPL16 |  |
| PITX2 | SCG5 |  |
| MAD2L1 | TSC22D4 |  |
| HPGD | SMAD3 |  |
| ASB5 | RCCD1 |  |
| EDIL3 | MBD6 |  |
| STARD4 | PDIA3 |  |
| F2RL2 | GNGT2 |  |
| GPX8 | PHB |  |
| CMYA5 | DOLPP1 |  |
| CSF2 | COQ7 |  |
| CGAS | FBXO22 |  |
| FABP7 | GNG8 |  |
| TXLNB | KLHL26 |  |
| DCBLD1 | DHRS13 |  |
| TMEM200A | LAIR2 |  |
| IL31RA | YIF1B |  |
| RAET1E | PLIN4 |  |
| PI16 | GLOD4 |  |
| STK17A | KLK1 |  |
| MYOZ3 | KRT80 |  |
| PTTG1 | NDUFV1 |  |
| KCNK5 | MRPL58 |  |
| SLC29A4 | ATP5PD |  |
| ZNF704 | SRP68 |  |
| COL1A2 | TK1 |  |
| FNDC1 | GHDC |  |
| SLC13A4 | ECI1 |  |
| SBSPON | KCTD5 |  |
| PHKG1 | POLR2G |  |
| EN2 | SPINDOC |  |
| DEFB1 | FADD |  |
| CA3 | COPS6 |  |
| OSR2 | KCNJ4 |  |
| BAALC | RNF187 |  |
| FZD6 | DDIT4 |  |
| CTHRC1 | LMBRD1 |  |
| LETM2 | DNAJC7 |  |
| MAMDC2 | THAP11 |  |
| ALDH1A1 | MPLKIP |  |
| SVEP1 | STIP1 |  |
| TRPV6 | ING2 |  |
| CLDN3 | SNRNP48 |  |
| WNK2 | PPIP5K1 |  |
| AQP3 | TSPAN5 |  |
| MELK | DDX19A |  |
| CFL2 | USP39 |  |
| SLC16A9 | INPP5D |  |
| CRYL1 | LETM1 |  |
| SKA3 | MFF |  |
| DDIAS | LGALS9 |  |
| PKNOX2 | E2F6 |  |
| DEPP1 | MAP2K1 |  |
| FAAH2 | NSMCE1 |  |
| BEND7 | RSPO1 |  |
| QSOX2 | TBC1D10B |  |
| SPACA9 | LMAN2 |  |
| NDRG2 | SLC50A1 |  |
| VWA2 | EFNA1 |  |
| ANKRD2 | MRPL1 |  |
| E2F7 | SCN9A |  |
| LARGE2 | HINT1 |  |
| RAPSN | DFFB |  |
| TC2N | CHRNA5 |  |
| IFI27 | HNRNPF |  |
| CLMN | CSGALNACT2 | |
| HTRA1 | WNT10B |  |
| TCP11L2 | TOR1AIP2 |  |
| GPR176 | FRMPD4 |  |
| ADAMTS15 | SF3B5 |  |
| GPT2 | YWHAG |  |
| IKBIP | KCNAB3 |  |
| ASPG | SERPINA9 |  |
| SCN3B | SIMC1 |  |
| C2 | TMEM192 |  |
| APBB1 | STX8 |  |
| SYNPO2L | CDK1 |  |
| CYB5A | B3GNT2 |  |
| USP54 | TMED10 |  |
| RAG1 | TMEM182 |  |
| TUB | KRT8 |  |
| CENPN | ADORA2B |  |
| MFAP4 | SLC23A1 |  |
| MCM7 | PA2G4 |  |
| A2ML1 | ELOVL6 |  |
| SEC11C | ARL6IP1 |  |
| RRAD | EMB |  |
| MMP10 | NUDCD2 |  |
| PLEKHA7 | HSPA4 |  |
| BMERB1 | ARMC10 |  |
| PCLAF | RNF34 |  |
| LDHD | ATF7 |  |
| PLIN1 | HOXB9 |  |
| SCNN1G | USP32 |  |
| C18orf54 | TSEN34 |  |
| PLK1 | MSANTD4 |  |
| NEMP1 | TRMT61B |  |
| ELFN2 | C1GALT1C1 | |
| MTMR10 | NAIF1 |  |
| C15orf48 | FAM241B |  |
| SCG5 | ZDHHC16 |  |
| GREM1 | PGAM1 |  |
| EVA1C | ZNF581 |  |
| NKX3-1 | NAT1 |  |
| MEI1 | KRT20 |  |
| B4GALNT2 | HOPX |  |
| TMEM92 | RSL1D1 |  |
| CERCAM | PPID |  |
| PRR15L | TBCA |  |
| GPRC5B | NEUROD2 |  |
| MYO5B | SLC25A33 |  |
| ACAA2 | RGS19 |  |
| FN3K | HDAC3 |  |
| LPO | SLFNL1 |  |
| JSRP1 | RHNO1 |  |
| CDT1 | NINJ2 |  |
| CORO6 | RPS21 |  |
| RHEBL1 | RAB33B |  |
| GPD1 | ORMDL3 |  |
| AXL | SMN1 |  |
| TMC4 | CYCS |  |
| NLRP7 | CCL11 |  |
| PSCA | TEFM |  |
| PLIN4 | MBOAT1 |  |
| GPT | CXCR6 |  |
| RILP | TPSAB1 |  |
| TRPV3 | IL16 |  |
| GGT6 | DNAJB7 |  |
| KLK1 | SNTG2 |  |
| KLK5 | KLHL6 |  |
| KLK11 | CHCHD1 |  |
| KLK13 | CORO1B |  |
| KRT1 | COL6A5 |  |
| ACER1 | RPL38 |  |
| RCOR2 | OVOL1 |  |
| IGFBP6 | SSH3 |  |
| HID1 | PDP2 |  |
| EVPL | DHCR7 |  |
| TMC8 | CCDC96 |  |
| TK1 | FAM222B |  |
| SLC3A2 | NOC3L |  |
| SPINDOC | MTX1 |  |
| FADD | CYSLTR1 |  |
| BATF2 | PARP15 |  |
| MAP4K2 | CKS1B |  |
| SCARA3 | ABCD2 |  |
| PBK | MFSD4B |  |
| SCARA5 | MZT2A |  |
| PDHB | OLR1 |  |
| FAM107A | NAA20 |  |
| CX3CR1 | SAA1 |  |
| PPDPFL | NUDT4 |  |
| XIRP1 | SLC19A1 |  |
| DEGS2 | RCE1 |  |
| TAP1 | UQCRH |  |
| KLHL30 | TOMM20 |  |
| SCNN1B | EIF1 |  |
| RAB31 | NRROS |  |
| TNXB | FBXO45 |  |
| BMP1 | CBY2 |  |
| PHYHIP | C16orf91 |  |
| FEN1 | TRMT10C |  |
| CAVIN2 | DDX23 |  |
| HJV | ATP2A2 |  |
| SERINC2 | C12orf76 |  |
| MYL1 | LINGO2 |  |
| COL3A1 | SLC26A9 |  |
| IL7R | MYO1H |  |
| WFDC12 | TMEM81 |  |
| NPNT | MRPL11 |  |
| FAM178B | GOLT1A |  |
| SHOX2 | UGT8 |  |
| ATOH8 | BTC |  |
| ROR2 | CNIH2 |  |
| AR | CD164L2 |  |
| PARM1 | FUT1 |  |
| FAM110B | KLC2 |  |
| AFAP1L2 | CTBP2 |  |
| PCSK9 | CHST2 |  |
| RAB3B | MRPS22 |  |
| CXCL10 | INHBC |  |
| CXCL11 | PCCA |  |
| GPRIN1 | BANF1 |  |
| HSPB3 | PCSK1 |  |
| NPR1 | TBC1D10C |  |
| CXCL8 | SART1 |  |
| SCN9A | TSGA10IP |  |
| COL22A1 | RMI2 |  |
| SPRR1A | B3GNTL1 |  |
| CRCT1 | RUVBL1 |  |
| MUC15 | MSRA |  |
| CLIC3 | TMEM270 |  |
| BNC1 | TSPEAR |  |
| CKAP2L | ARL4D |  |
| BUB1 | UBE2O |  |
| RAC3 | BNIP3 |  |
| UGP2 | LRRTM4 |  |
| PCDH7 | ACBD7 |  |
| TPST1 | SYNE3 |  |
| TAS1R3 | B3GNT5 |  |
| MYO7B | LRRC37A |  |
| RNF150 | GCNT4 |  |
| FABP6 | NFATC2IP |  |
| FAXDC2 | DPP7 |  |
| SLN | SLC38A9 |  |
| CDK1 | FAM210A |  |
| FOS | DEFB4B |  |
| SETMAR | KCNA3 |  |
| CST2 | PAWR |  |
| GPRC5C | TMEM94 |  |
| TMEM182 | KCNJ10 |  |
| KRT78 | AP3S1 |  |
| KRT75 | GRB2 |  |
| KRT4 | UBE2N |  |
| ELOVL6 | RPS27 |  |
| TMC7 | SPINK6 |  |
| SMAGP | ZNF366 |  |
| CDH2 | ZC3H12D |  |
| SIX2 | SHISA3 |  |
| STAT2 | CALML5 |  |
| SGCD | ZFAND2A |  |
| GTSF1 | NEUROG2 |  |
| ACYP2 | CD28 |  |
| CAVIN4 | DYNAP |  |
| HOXB9 | SUZ12 |  |
| CDCA4 | RPP25 |  |
| HTRA3 | GP5 |  |
| LMOD2 | C5orf46 |  |
| GPR27 | RFLNA |  |
| OSCAR | TAF7 |  |
| PAQR8 | TUFM |  |
| CAVIN3 | RMI1 |  |
| HAS2 | MRFAP1 |  |
| PDGFD | KLHDC7A |  |
| PLAC1 | TRIML2 |  |
| S1PR1 | RCC2 |  |
| PYGO1 | TCAIM |  |
| PKIA | CLK3 |  |
| FUT3 | PACS2 |  |
| ATP6V0E2 | ARL14 |  |
| MUC7 | LRRC3B |  |
| SMR3B | RNF227 |  |
| NETO2 | PUF60 |  |
| LRG1 | DCTPP1 |  |
| SHCBP1 | CCDC43 |  |
| CHST11 | FUT7 |  |
| ESCO2 | MB21D2 |  |
| TPPP | MAP3K15 |  |
| APLN | SSR4 |  |
| KRT13 | PITPNB |  |
| KRT9 | METTL23 |  |
| PDE7B | NPM1 |  |
| HOPX | PJA1 |  |
| LRRC8C | H2AW |  |
| COL24A1 | FRAT2 |  |
| ETFDH | HEPHL1 |  |
| LPAR3 | RAP2B |  |
| PIK3CD | MRPS23 |  |
| P2RY6 | TIGIT |  |
| ANO5 | RNF41 |  |
| GATM | COA4 |  |
| KNDC1 | PRKAG1 |  |
| COL8A2 | MRPS11 |  |
| RRM2 | SNRPE |  |
| PRNP | MRPL41 |  |
| FRMD5 | ASB18 |  |
| CYP4F11 | SHMT2 |  |
| CYP4F22 | GABRG3 |  |
| MAL | FIGN |  |
| LRRC15 | C8orf33 |  |
| CCL11 | KPNA2 |  |
| FRMD3 | GLRX5 |  |
| SNTB1 | HS3ST4 |  |
| MTBP | SKA2 |  |
| ID4 | C16orf72 |  |
| CLEC7A | RPL35A |  |
| NEGR1 | HMGN4 |  |
| CLEC12A | ZNF662 |  |
| SUCLG2 | PYCR1 |  |
| RCAN2 | NEB |  |
| PRSS27 | CEP57L1 |  |
| MYOZ2 | RUVBL2 |  |
| SYNPO2 | PTTG1IP |  |
| MAB21L4 | DDX41 |  |
| CARNS1 | PLGLB1 |  |
| MUCL1 | DAZAP2 |  |
| KLHL6 | SELENOF |  |
| RND1 | CCDC125 |  |
| CCL19 | TMEM89 |  |
| LRRC20 | RIPK4 |  |
| FADS6 | GPR132 |  |
| CYP7B1 | COA5 |  |
| CES3 | PSMG1 |  |
| NBEA | SETD3 |  |
| ARPP21 | H3C13 |  |
| ADCY5 | TRMT12 |  |
| PARP14 | IFNL2 |  |
| CYSLTR1 | CMTM4 |  |
| CKS1B | TBL3 |  |
| MAB21L3 | CCR4 |  |
| SYT12 | LIN9 |  |
| OLR1 | SH2D1A |  |
| SAA1 | PTP4A2 |  |
| CSPG4 | ACTG1 |  |
| SULT1B1 | NIPSNAP1 |  |
| NUDT4 | SCFD2 |  |
| LRFN4 | CMSS1 |  |
| SLC19A1 | H1-5 |  |
| HSPB7 | NCMAP |  |
| HAP1 | KCNH7 |  |
| CBX2 | APOO |  |
| MARCHF3 | TMED9 |  |
| TCAP | SUMO3 |  |
| CD34 | IMMP2L |  |
| CTSF | JAG2 |  |
| MSRB3 | WT1 |  |
| TLR6 | AP3M1 |  |
| SELP | SEMA4B |  |
| SNX31 | C5orf47 |  |
| ADCY6 | FAF1 |  |
| GLIS1 | TCEAL9 |  |
| PODN | IL3RA |  |
| EXO1 | HS6ST3 |  |
| ABRA | HGS |  |
| ATP2A2 | MRPL30 |  |
| CNTNAP2 | BRCC3 |  |
| SLC26A9 | TLCD2 |  |
| MFSD4A | INKA1 |  |
| KY | UBE2L3 |  |
| SLC29A2 | PMEL |  |
| FZD4 | POU3F1 |  |
| ADGRE1 | DRG1 |  |
| CD164L2 | C11orf87 |  |
| SLC22A1 | MORF4L1 |  |
| CHST2 | PCYT2 |  |
| UBE2C | BCAP31 |  |
| DES | GNB1L |  |
| MARCKSL1 | TMPRSS11B | |
| ABO | TAS2R60 |  |
| PCCA | IRS2 |  |
| PHYHD1 | LCE3A |  |
| CCNE2 | C2orf76 |  |
| NRIP3 | POLR1D |  |
| LPL | KIF18B |  |
| CCDC14 | BPIFB4 |  |
| UCP3 | SAPCD2 |  |
| SUGCT | LCE5A |  |
| RMI2 | BTLA |  |
| MTLN | TOR3A |  |
| MSRA | PPP1CC |  |
| CREG2 | PRELID2 |  |
| HOXD8 | SLC36A2 |  |
| A2M | TMPRSS12 |  |
| DOK7 | INSIG1 |  |
| LRRN1 | LYRM7 |  |
| CRYBG2 | TNFRSF4 |  |
| TCP11L1 | KRT16 |  |
| ATAD5 | ESPN |  |
| RTTN | NAP1L1 |  |
| B3GNT4 | LCE2A |  |
| HSD11B2 | BCAM |  |
| RIMS2 | RSBN1L |  |
| PLAAT3 | EPOR |  |
| DIRAS1 | USP7 |  |
| GNG7 | SECISBP2 |  |
| KBTBD11 | C9orf153 |  |
| FOXL1 | ASCL4 |  |
| FOXC2 | TTC24 |  |
| BASP1 | S100A3 |  |
| SOX11 | RILPL1 |  |
| MAMSTR | EYS |  |
| FUT2 | MAPK12 |  |
| MUC20 | TMEM215 |  |
| SHMT1 | LAMTOR4 |  |
| TRIM72 | PRKAR1B |  |
| C10orf71 | AGAP4 |  |
| LRRN4CL | COMMD6 |  |
| SAMD9L | GTF2F2 |  |
| HASPIN | ZP3 |  |
| PNPLA2 | NBR1 |  |
| AGTRAP | FAM72B |  |
| CRACR2B | SUMO2 |  |
| NAALADL2 | PARVB |  |
| SOX12 | SMIM15 |  |
| MYOZ1 | TMEM201 |  |
| PCDHB9 | NHLRC3 |  |
| ASB8 | CNR2 |  |
| CALHM5 | HACD4 |  |
| GRAMD1C | NOC2L |  |
| PDE4DIP | NDUFA4 |  |
| ZNF114 | JPT1 |  |
| SPINK6 | TSPYL1 |  |
| RNF212 | SLC35E2B |  |
| GLDC | MYBPC1 |  |
| P4HTM | AKR1C3 |  |
| SLC25A20 | S100A4 |  |
| DYNAP | MPHOSPH8 | |
| STX19 | LCOR |  |
| ERFE | SUPT3H |  |
| CPNE7 | IARS1 |  |
| C5orf46 | LONP1 |  |
| TMEM52 | XRCC6 |  |
| MSC | PPP1R26 |  |
| APOLD1 | SULT1A1 |  |
| SMIM10L2A | ARL9 |  |
| AURKB | PRPF40A |  |
| TRIML2 | FAM72A |  |
| TMEM125 | HDAC2 |  |
| CDH4 | ADH1B |  |
| EGR3 | TCF4 |  |
| FJX1 | SDHAF3 |  |
| ALOX15B | ZKSCAN5 |  |
| FCER1A | TOMM7 |  |
| ARL14 | ZNF33B |  |
| PCED1B | AMZ2 |  |
| APOBEC3B | H2AC11 |  |
| PIPOX | STRN3 |  |
| TH | FAM3C |  |
| MYLPF | METTL9 |  |
| FGD6 | PCBP2 |  |
| FZD2 | ZNF257 |  |
| MTURN | H3C12 |  |
| C11orf71 | PSMD12 |  |
| BHLHA15 | TPSB2 |  |
| TSPYL5 | MRPL21 |  |
| H2BC4 | DIO3 |  |
| MB21D2 | HNRNPAB |  |
| SLC47A2 | PDGFA |  |
| PRF1 | GYPE |  |
| S1PR5 | RPF2 |  |
| ZFP3 | GZMM |  |
| ARSJ | TOPORS |  |
| HOXC10 | KLHL14 |  |
| CXCR2 | HOXC6 |  |
| RPH3AL | MCMBP |  |
| SLC25A42 | ZNF780A |  |
| MAPK15 | ATAD3A |  |
| ADIPOQ | ADH5 |  |
| F2R | CLEC9A |  |
| ZNF707 | DLGAP2 |  |
| DDX60L | MRPL42 |  |
| FANCB | MARCHF5 |  |
| FDCSP | NUP62CL |  |
| TNFSF15 | MB |  |
| PLAG1 | TFDP1 |  |
| AMIGO1 | CLEC4C |  |
| TIGIT | DDX42 |  |
| SLC2A4 | RPL23A |  |
| CLDN7 | TXNRD1 |  |
| CHST15 | GPN1 |  |
| IDH2 | ZNF536 |  |
| FAM89A | RYR2 |  |
| UNC5C | LPA |  |
| RGMA | SLC9A6 |  |
| LDOC1 | F5 |  |
| EXT1 | RPL10A |  |
| HHIPL1 | GRIN3A |  |
| SYNM | SFT2D1 |  |
| NLRP10 | RYR3 |  |
| B4GALNT4 | ZNF277 |  |
| AP1S2 | STYXL2 |  |
| FBXL6 | SELENOT |  |
| NXPH4 | OSTC |  |
| KPNA2 | ASB12 |  |
| BGN | TOP1 |  |
| FES | RPL39 |  |
| CAV3 | DCLRE1A |  |
| RNASE10 | CCDC167 |  |
| SATB1 | INF2 |  |
| TRAK1 | CAPN8 |  |
| TSPAN10 | TATDN3 |  |
| NTM | DDO |  |
| PPP1R27 | GDI1 |  |
| C1orf116 | TCEAL5 |  |
| OTOP3 | SYS1 |  |
| GJC1 | ASAH2B |  |
| ZNF662 | TIMM23B |  |
| CADM1 | DAXX |  |
| SPNS2 | BRD2 |  |
| NEB | PBX2 |  |
| SMDT1 | RNF5 |  |
| CHST6 | SDHD |  |
| CCBE1 | NEU1 |  |
| GBP6 | VWA7 |  |
| FHL3 | DHX16 |  |
| MX2 | MRPS18B |  |
| TENT5C | ABCF1 |  |
| TNFAIP8L3 | DDR1 |  |
| BMP8A | ZNF616 |  |
| ASCL2 | RACK1 |  |
| MACC1 | MZT1 |  |
| FOXL2 | FAM71F2 |  |
| B3GALT5 | E2F4 |  |
| GPR39 | IPO7 |  |
| FAM3B | HMGN1 |  |
| KIRREL1 | CRLF2 |  |
| IQGAP3 | CRYZL1 |  |
| ARSI | ARRDC5 |  |
| TMPRSS2 | CLEC6A |  |
| CLDN5 | RNPS1 |  |
| SPRR4 | DNAJC19 |  |
| C1QTNF12 | JPT2 |  |
| KCNJ12 | GOLGA8O |  |
| ACOT1 | COL6A6 |  |
| ALDH1A3 | RAB12 |  |
| POU6F1 | TSN |  |
| SRPK3 | KLHL23 |  |
| SLIT3 | ANKRD39 |  |
| EFNA5 | CHUK |  |
| MAP7D2 | COG8 |  |
| KNTC1 | ARHGAP19 |  |
| FOXO4 | SYNJ2BP |  |
| PTP4A3 | RBMXL1 |  |
| GAST | DNAJC9 |  |
| C6orf58 | VDAC1 |  |
| PDE4B | TMX2 |  |
| NELL2 | HEXA |  |
| KRBA2 | ZNF134 |  |
| CDCA2 | AP1G2 |  |
| ZBTB7C | MYCBP |  |
| USP18 | ALG3 |  |
| SORCS2 | BBIP1 |  |
| ROBO2 | PPME1 |  |
| F8 | C10orf105 |  |
| CA13 | EVPLL |  |
| MROH2A | HOMEZ |  |
| KRT76 | TMEM242 |  |
| FLRT2 | CEBPZOS |  |
| ADSS1 | UMAD1 |  |
| INPP5J | PPT2 |  |
| MAGEA11 | ADM5 |  |
| NOTUM | FTCDNL1 |  |
| GALNT17 | AC013470.2 | |
| SOCS1 | ERVMER34-1 | |
| TEDC1 | WDR46 |  |
| METTL7A | PARG |  |
| FAM174B | AKR1B15 |  |
| STAC3 | LTB |  |
| ROR1 | TEX46 |  |
| IRF7 | POLR2J2 |  |
| SV2B | OST4 |  |
| PRKG1 | RPS18 |  |
| DLK1 | MCTS1 |  |
| LSAMP | RNF224 |  |
| OLFML2A | UQCRHL |  |
| PBX1 | IQCM |  |
| KRT79 | KIAA0040 |  |
| PMEL | CDKN2AIPNL | |
| POU3F1 | ZNF737 |  |
| PRAME | LILRA4 |  |
| MYBL1 | TNFRSF13B | |
| SRL | AQP1 |  |
| IFIT1 | RDH14 |  |
| SLC52A2 | NSUN6 |  |
| DNAH14 | CRCP |  |
| TMPRSS11B | ATP5MF |  |
| IFITM1 | PISD |  |
| LEMD1 | C22orf39 |  |
| CD300LF | GNG10 |  |
| KIF18B | CCDC169 |  |
| CYP4F12 | MRPL33 |  |
| THBS2 | MRPS6 |  |
| ANKRD37 | NFS1 |  |
| CYP4X1 | CFHR1 |  |
| KRT10 | PGAM5 |  |
| TRDN | MARS2 |  |
| KRT3 | APELA |  |
| KLK12 | PRR5-ARHGAP8 | |
| CYP27C1 | STIMATE-MUSTN1 | |
| LYRM7 | HS3ST5 |  |
| VSIG10L | CHCHD10 |  |
| LILRB4 | PCDHGA6 |  |
| TNFRSF4 | ETV3L |  |
| TRABD2A | PCDHGA3 |  |
| MAPT | AP002495.1 | |
| ERCC6L | TIFAB |  |
| TNFRSF18 | AL590560.2 |  |
| KANK3 | GATC |  |
| TMPRSS11A | CNPY2 |  |
| MITF | CTXND1 |  |
| AKR1C1 | HOXB7 |  |
| MT1X | KCNJ18 |  |
| FPR3 | CCNQ |  |
| C11orf96 | ZNF234 |  |
| KCNJ11 | AC119396.1 | |
| COL4A1 | FAM72C |  |
| PRR27 | RHEX |  |
| TLR5 | DYNLL2 |  |
| ISG15 | ANXA8 |  |
| PERM1 | RPL17 |  |
| SPRY4 | GDF10 |  |
| AMTN | FXYD1 |  |
| C2orf88 | AC090360.1 | |
| KBTBD12 | MAGIX |  |
| THSD4 | TAF15 |  |
| FANCA | NUDT3 |  |
| DNAH17 | POM121C |  |
| DMBT1 | ATP6V1FNB | |
| COL14A1 | AL049839.2 |  |
| DNER | ZNHIT3 |  |
| TPRG1 | H3C8 |  |
| WNT7B | AL136531.2 |  |
| COL4A5 | RCC1L |  |
| AGRN | H2AC12 |  |
| SMTNL2 | ZNF2 |  |
| ZNF793 | FCGBP |  |
| PLA2G2A | HNF1B |  |
| C15orf62 | AATF |  |
| HES4 | H2BC9 |  |
| SBK1 | TUBGCP5 |  |
| IER5L | H2AC16 |  |
| NCCRP1 | PCGF2 |  |
| HBA2 | H3C7 |  |
| DUSP28 | SRD5A2 |  |
| CCDC9B | CISD3 |  |
| IGFL3 | RDM1 |  |
| SPRED3 | SSTR3 |  |
| PRELP | H2BC10 |  |
| LRRK2 | MRM1 |  |
| CFAP126 | MRPL45 |  |
| SBSN | SLURP2 |  |
| RNF222 | AC093827.5 | |
| RELN | AC022415.2 | |
| FAM111B | AC055839.2 | |
| APOD | H3C2 |  |
| PLAC9 |  |  |
| CLDN4 |  |  |
| S100A14 |  |  |
| NEMP2 |  |  |
| ALG1L |  |  |
| CXCL17 |  |  |
| SH2D5 |  |  |
| ZFP92 |  |  |
| GJB4 |  |  |
| SYCP2 |  |  |
| MYBPC1 |  |  |
| PAX5 |  |  |
| AKR1C3 |  |  |
| ACADSB |  |  |
| TMEM63A |  |  |
| RYR1 |  |  |
| ZNF493 |  |  |
| ATP2A1 |  |  |
| STK31 |  |  |
| ADH7 |  |  |
| NUDT11 |  |  |
| MYL6B |  |  |
| SIAH1 |  |  |
| SPTSSB |  |  |
| SULF2 |  |  |
| AJAP1 |  |  |
| XRCC2 |  |  |
| MMP1 |  |  |
| ADH1B |  |  |
| COL27A1 |  |  |
| MAML3 |  |  |
| H2AC11 |  |  |
| SPRR2B |  |  |
| ADA |  |  |
| ZFP28 |  |  |
| CRACDL |  |  |
| SCN8A |  |  |
| LAMB3 |  |  |
| ZNF429 |  |  |
| ZNF470 |  |  |
| ANXA6 |  |  |
| ZNF420 |  |  |
| LCE1C |  |  |
| MAGEA6 |  |  |
| CYSRT1 |  |  |
| C6orf141 |  |  |
| FITM2 |  |  |
| SVIL |  |  |
| LYPD2 |  |  |
| OGDHL |  |  |
| COL13A1 |  |  |
| SLC2A10 |  |  |
| SLC28A3 |  |  |
| COL4A6 |  |  |
| MYH6 |  |  |
| SERPINB13 | |  |
| PDCD1LG2 |  |  |
| NMB |  |  |
| HOXC6 |  |  |
| CFD |  |  |
| OCLN |  |  |
| ADAMTSL2 |  |  |
| PRB3 |  |  |
| NRAP |  |  |
| H2BC12 |  |  |
| ZNF347 |  |  |
| AADACL2 |  |  |
| ENTPD7 |  |  |
| NUP62CL |  |  |
| TMPRSS11F | |  |
| CHSY3 |  |  |
| TOR4A |  |  |
| MB |  |  |
| TMEM229B |  |  |
| HMGN5 |  |  |
| SVIP |  |  |
| BPIFA1 |  |  |
| CARD11 |  |  |
| MYL4 |  |  |
| HOXC4 |  |  |
| ZNF568 |  |  |
| TPM2 |  |  |
| SH3BGRL2 |  |  |
| ANKRD35 |  |  |
| ATL1 |  |  |
| ZNF43 |  |  |
| PLN |  |  |
| WDHD1 |  |  |
| MMP17 |  |  |
| CCDC69 |  |  |
| RYR2 |  |  |
| FAM3D |  |  |
| STK39 |  |  |
| MAGEA1 |  |  |
| ABCA4 |  |  |
| MT-ND6 |  |  |
| MT-CYB |  |  |
| PPP1R14C |  |  |
| F5 |  |  |
| EGFL6 |  |  |
| MT-ND2 |  |  |
| RCSD1 |  |  |
| MT-ND5 |  |  |
| ALPK2 |  |  |
| MT-CO1 |  |  |
| PAX9 |  |  |
| GK |  |  |
| ARHGAP11A | |  |
| RYR3 |  |  |
| MT-ND3 |  |  |
| STYXL2 |  |  |
| TOX |  |  |
| C1orf68 |  |  |
| MT-ND4 |  |  |
| MT-ND1 |  |  |
| SHISA4 |  |  |
| CIPC |  |  |
| MT-ATP6 |  |  |
| L1CAM |  |  |
| RASGEF1A |  |  |
| DZIP3 |  |  |
| CSAG1 |  |  |
| GPRASP1 |  |  |
| MT-CO3 |  |  |
| DMD |  |  |
| SAMD5 |  |  |
| FCGR3A |  |  |
| CENPW |  |  |
| LORICRIN |  |  |
| PRR9 |  |  |
| SPRR2E |  |  |
| PLPP4 |  |  |
| GGTA1 |  |  |
| TCEA3 |  |  |
| COL5A2 |  |  |
| TAP2 |  |  |
| SMIM5 |  |  |
| LAYN |  |  |
| SLC44A4 |  |  |
| VWA7 |  |  |
| LY6G6C |  |  |
| MICB |  |  |
| HLA-C |  |  |
| PSORS1C2 |  |  |
| PSORS1C1 |  |  |
| C6orf15 |  |  |
| MUC21 |  |  |
| HLA-G |  |  |
| HLA-F |  |  |
| IGFL2 |  |  |
| ZNF425 |  |  |
| VIT |  |  |
| PDE7A |  |  |
| TMEM170B |  |  |
| INSYN1 |  |  |
| MT1M |  |  |
| SAMD9 |  |  |
| EXOC3L4 |  |  |
| HTN3 |  |  |
| NYNRIN |  |  |
| SERPINB11 | |  |
| HLA-A |  |  |
| GPX3 |  |  |
| DIO2 |  |  |
| RNF208 |  |  |
| MT-ND4L |  |  |
| ACKR1 |  |  |
| TRIM59 |  |  |
| MAGEA12 |  |  |
| DDAH2 |  |  |
| IRF9 |  |  |
| ITGA1 |  |  |
| TMEM213 |  |  |
| ALG3 |  |  |
| SH3D21 |  |  |
| CAPN14 |  |  |
| SMTNL1 |  |  |
| MUC5AC |  |  |
| UBE2QL1 |  |  |
| RPTN |  |  |
| TENM3 |  |  |
| MAGEA3 |  |  |
| ZNF880 |  |  |
| FADS3 |  |  |
| ZNF844 |  |  |
| C4B |  |  |
| ZNF469 |  |  |
| SLC26A6 |  |  |
| ELFN1 |  |  |
| ERVMER34-1 | |  |
| MT-ATP8 |  |  |
| HLA-B |  |  |
| C12orf75 |  |  |
| ZNF853 |  |  |
| RNF223 |  |  |
| KIFC1 |  |  |
| OR2I1P |  |  |
| KLHL41 |  |  |
| STRIT1 |  |  |
| PSMB9 |  |  |
| LCE1F |  |  |
| AQP1 |  |  |
| PCDHGC5 |  |  |
| INMT |  |  |
| SPRR2A |  |  |
| RTL5 |  |  |
| C4orf48 |  |  |
| UPK3B |  |  |
| SPRR2F |  |  |
| LILRA6 |  |  |
| SCARF2 |  |  |
| ASPRV1 |  |  |
| C4A |  |  |
| HBB |  |  |
| ADH1C |  |  |
| PCP4L1 |  |  |
| C4orf54 |  |  |
| TMEM150C |  |  |
| TMEM158 |  |  |
| TRMT9B |  |  |
| CHCHD10 |  |  |
| FOXD1 |  |  |
| HOXA10 |  |  |
| TMEM200B |  |  |
| C1orf210 |  |  |
| TRNP1 |  |  |
| PCDHGB2 |  |  |
| CLDN23 |  |  |
| SIGLEC12 |  |  |
| TRIL |  |  |
| SMIM3 |  |  |
| ZNF253 |  |  |
| HOXB7 |  |  |
| TMEM178B |  |  |
| BOP1 |  |  |
| MMP12 |  |  |
| MSMB |  |  |
| RHEX |  |  |
| TXNIP |  |  |
| EDDM13 |  |  |
| MAGIX |  |  |
| MMP28 |  |  |
| CCL5 |  |  |
| CD24 |  |  |
| DOC2B |  |  |
| AGBL1 |  |  |
| LHX1 |  |  |
| H2BC8 |  |  |
| NATD1 |  |  |
| FCGBP |  |  |
| H2BC9 |  |  |
| UHRF1 |  |  |
| H4C9 |  |  |
| H2AC8 |  |  |
| NEFL |  |  |
| AC007906.2 | |  |
| H3C10 |  |  |
| BLACAT1 |  |  |
| IQCJ-SCHIP1 | |  |
| SPRR5 |  |  |
| PRR33 |  |  |
| EEF1AKMT4 | |  |
| NPBWR1 |  |  |
