# Supplementary material for: HMMR as a robust prognostic biomarker correlates with immune infiltration and cell cycle pathways in oral squamous cell carcinoma: a multi-cohort bioinformatics analysis based on TCGA and GEO databases
Source: Front Genet. 2026 Jun 5;17:1764943. doi: 10.3389/fgene.2026.1764943 (PMC13278685; doi:10.3389/fgene.2026.1764943)
Supplement: Supplementary file 8 [file Table2.docx]

| **#node1** | **node2** | **coexpression** | **Experimentally**  **Determined**  **interaction** | **Database**  **annotated** | **Automated**  **textmining** | **combined_score** |
| --- | --- | --- | --- | --- | --- | --- |
| ARHGEF39 | SGO1 | 0.403 | 0 | 0 | 0 | 0.403 |
| ARHGEF39 | KIF18B | 0.4 | 0 | 0 | 0 | 0.4 |
| ASF1B | CDC6 | 0.411 | 0 | 0 | 0.427 | 0.648 |
| ASF1B | CENPM | 0.409 | 0 | 0 | 0.499 | 0.692 |
| ASF1B | AURKA | 0.51 | 0 | 0 | 0.513 | 0.751 |
| ASF1B | ORC6 | 0.267 | 0 | 0 | 0.387 | 0.532 |
| ASF1B | PIMREG | 0.372 | 0 | 0 | 0.114 | 0.42 |
| ASF1B | NCAPG | 0.385 | 0 | 0 | 0.193 | 0.483 |
| ASF1B | TROAP | 0.42 | 0 | 0 | 0.137 | 0.478 |
| ASF1B | KIF11 | 0.507 | 0 | 0 | 0.368 | 0.675 |
| ASF1B | SKA3 | 0.381 | 0 | 0 | 0.071 | 0.4 |
| ASF1B | CDC25C | 0.368 | 0 | 0 | 0.091 | 0.401 |
| ASF1B | DEPDC1B | 0.303 | 0 | 0 | 0.185 | 0.409 |
| ASF1B | NEK2 | 0.351 | 0 | 0 | 0.167 | 0.436 |
| ASF1B | SPC25 | 0.374 | 0 | 0 | 0.148 | 0.444 |
| ASF1B | ZWINT | 0.369 | 0 | 0 | 0.22 | 0.487 |
| ASF1B | NUF2 | 0.388 | 0 | 0 | 0.2 | 0.489 |
| ASF1B | UBE2T | 0.363 | 0.095 | 0 | 0.189 | 0.492 |
| ASF1B | MAD2L1 | 0.428 | 0 | 0 | 0.18 | 0.511 |
| ASF1B | KIF18B | 0.381 | 0 | 0 | 0.249 | 0.515 |
| ASF1B | SGO1 | 0.418 | 0 | 0 | 0.207 | 0.518 |
| ASF1B | HMMR | 0.343 | 0.089 | 0 | 0.279 | 0.531 |
| ASF1B | CENPE | 0.282 | 0 | 0 | 0.402 | 0.552 |
| ASF1B | CCNB2 | 0.477 | 0.094 | 0 | 0.199 | 0.587 |
| ASF1B | TPX2 | 0.458 | 0 | 0 | 0.284 | 0.596 |
| ASF1B | KIF4A | 0.496 | 0 | 0 | 0.27 | 0.616 |
| ASF1B | CEP55 | 0.37 | 0 | 0 | 0.434 | 0.628 |
| ASF1B | PTTG1 | 0.493 | 0 | 0 | 0.31 | 0.636 |
| ASF1B | CDK1 | 0.493 | 0.095 | 0 | 0.282 | 0.641 |
| ASF1B | TK1 | 0.436 | 0 | 0 | 0.401 | 0.648 |
| ASF1B | RAD54L | 0.364 | 0.097 | 0 | 0.45 | 0.657 |
| ASF1B | SPAG5 | 0.638 | 0.113 | 0 | 0.081 | 0.679 |
| ASF1B | RAD51 | 0.415 | 0.129 | 0 | 0.43 | 0.684 |
| ASF1B | CENPF | 0.357 | 0 | 0 | 0.592 | 0.726 |
| ASF1B | CCNA2 | 0.605 | 0 | 0 | 0.473 | 0.783 |
| ASF1B | CENPA | 0.393 | 0.377 | 0 | 0.588 | 0.831 |
| ASF1B | TONSL | 0.153 | 0.795 | 0 | 0.626 | 0.929 |
| AUNIP | AURKA | 0.267 | 0.617 | 0 | 0.203 | 0.757 |
| AUNIP | SKA3 | 0.356 | 0 | 0 | 0.22 | 0.477 |
| AUNIP | RAD54L | 0.345 | 0 | 0 | 0.134 | 0.409 |
| AUNIP | ECT2 | 0.174 | 0 | 0 | 0.34 | 0.432 |
| AUNIP | RAD51 | 0.368 | 0 | 0 | 0.144 | 0.436 |
| AURKA | CDC6 | 0.477 | 0.075 | 0 | 0.546 | 0.761 |
| AURKA | CENPM | 0.342 | 0 | 0 | 0.269 | 0.499 |
| AURKA | CENPK | 0.279 | 0.092 | 0 | 0.259 | 0.473 |
| AURKA | DEPDC1B | 0.498 | 0 | 0 | 0.058 | 0.507 |
| AURKA | RAD54L | 0.377 | 0.051 | 0 | 0.252 | 0.519 |
| AURKA | PIMREG | 0.411 | 0 | 0 | 0.223 | 0.523 |
| AURKA | KNSTRN | 0.429 | 0 | 0 | 0.229 | 0.541 |
| AURKA | SPC25 | 0.474 | 0 | 0 | 0.165 | 0.542 |
| AURKA | SKA3 | 0.452 | 0 | 0 | 0.199 | 0.542 |
| AURKA | TROAP | 0.497 | 0 | 0 | 0.165 | 0.563 |
| AURKA | BRCA2 | 0.178 | 0.071 | 0 | 0.495 | 0.581 |
| AURKA | TK1 | 0.43 | 0 | 0 | 0.325 | 0.598 |
| AURKA | KIF18B | 0.393 | 0.094 | 0 | 0.342 | 0.607 |
| AURKA | KPNA2 | 0.469 | 0 | 0 | 0.306 | 0.616 |
| AURKA | CCNA1 | 0.295 | 0 | 0 | 0.492 | 0.627 |
| AURKA | GMNN | 0.314 | 0.292 | 0 | 0.321 | 0.641 |
| AURKA | CKS1B | 0.452 | 0 | 0 | 0.378 | 0.645 |
| AURKA | UBE2T | 0.461 | 0.067 | 0 | 0.358 | 0.649 |
| AURKA | CENPI | 0.337 | 0.07 | 0 | 0.505 | 0.668 |
| AURKA | RAD51 | 0.451 | 0.071 | 0 | 0.407 | 0.671 |
| AURKA | NCAPD2 | 0.447 | 0.112 | 0 | 0.397 | 0.678 |
| AURKA | KNL1 | 0.306 | 0.063 | 0 | 0.568 | 0.695 |
| AURKA | SGO1 | 0.634 | 0 | 0 | 0.345 | 0.75 |
| AURKA | ASF1B | 0.51 | 0 | 0 | 0.513 | 0.751 |
| AURKA | AUNIP | 0.267 | 0.617 | 0 | 0.203 | 0.757 |
| AURKA | DEPDC1 | 0.701 | 0 | 0 | 0.3 | 0.782 |
| AURKA | ZWINT | 0.431 | 0 | 0 | 0.642 | 0.787 |
| AURKA | SPAG5 | 0.581 | 0.294 | 0 | 0.376 | 0.8 |
| AURKA | CKS2 | 0.476 | 0 | 0 | 0.642 | 0.804 |
| AURKA | NEK2 | 0.641 | 0 | 0 | 0.501 | 0.829 |
| AURKA | ECT2 | 0.495 | 0.071 | 0 | 0.67 | 0.831 |
| AURKA | CDC25C | 0.645 | 0 | 0 | 0.544 | 0.831 |
| AURKA | CENPE | 0.643 | 0.128 | 0 | 0.613 | 0.869 |
| AURKA | CDKN3 | 0.793 | 0.135 | 0 | 0.334 | 0.87 |
| AURKA | KIF4A | 0.692 | 0 | 0 | 0.642 | 0.885 |
| AURKA | NUF2 | 0.674 | 0.292 | 0 | 0.574 | 0.893 |
| AURKA | CENPF | 0.679 | 0 | 0 | 0.708 | 0.902 |
| AURKA | MAD2L1 | 0.647 | 0.1 | 0 | 0.796 | 0.929 |
| AURKA | CEP55 | 0.802 | 0.161 | 0 | 0.65 | 0.936 |
| AURKA | CCNA2 | 0.853 | 0 | 0 | 0.669 | 0.949 |
| AURKA | CDK1 | 0.777 | 0.525 | 0 | 0.537 | 0.951 |
| AURKA | NCAPG | 0.816 | 0.103 | 0 | 0.735 | 0.952 |
| AURKA | CENPA | 0.785 | 0.149 | 0 | 0.797 | 0.959 |
| AURKA | PTTG1 | 0.68 | 0.329 | 0.5 | 0.73 | 0.967 |
| AURKA | HMMR | 0.794 | 0.292 | 0 | 0.811 | 0.97 |
| AURKA | KIF11 | 0.812 | 0.272 | 0 | 0.816 | 0.972 |
| AURKA | BORA | 0.427 | 0.328 | 0.5 | 0.89 | 0.976 |
| AURKA | CCNB2 | 0.86 | 0.637 | 0 | 0.799 | 0.988 |
| AURKA | TPX2 | 0.846 | 0.988 | 0.9 | 0.995 | 0.999 |
| BORA | AURKA | 0.427 | 0.328 | 0.5 | 0.89 | 0.976 |
| BORA | NCAPG | 0.463 | 0 | 0 | 0.083 | 0.487 |
| BORA | KIF11 | 0.468 | 0 | 0 | 0.175 | 0.543 |
| BORA | SGO1 | 0.312 | 0 | 0 | 0.252 | 0.464 |
| BORA | DEPDC1B | 0.301 | 0 | 0 | 0.362 | 0.535 |
| BORA | CENPE | 0.385 | 0 | 0 | 0.217 | 0.498 |
| BORA | NUF2 | 0.394 | 0 | 0 | 0.101 | 0.432 |
| BORA | SPC25 | 0.364 | 0 | 0 | 0.216 | 0.48 |
| BORA | CCNB2 | 0.31 | 0 | 0.4 | 0.176 | 0.629 |
| BORA | MAD2L1 | 0.415 | 0 | 0 | 0.179 | 0.499 |
| BORA | TPX2 | 0.313 | 0 | 0 | 0.624 | 0.73 |
| BORA | SKA3 | 0.411 | 0 | 0 | 0 | 0.411 |
| BORA | CDC25C | 0.241 | 0 | 0 | 0.288 | 0.437 |
| BORA | SPAG5 | 0.254 | 0 | 0 | 0.24 | 0.409 |
| BORA | CENPA | 0.277 | 0 | 0 | 0.479 | 0.607 |
| BORA | NEK2 | 0.37 | 0 | 0.5 | 0.178 | 0.718 |
| BORA | CEP55 | 0.341 | 0 | 0 | 0.286 | 0.509 |
| BORA | KIF4A | 0.412 | 0 | 0 | 0.155 | 0.482 |
| BORA | ECT2 | 0.314 | 0 | 0 | 0.632 | 0.737 |
| BORA | CDK1 | 0.373 | 0 | 0.5 | 0.74 | 0.911 |
| BORA | DEPDC1 | 0.29 | 0 | 0 | 0.226 | 0.427 |
| BORA | CCNA2 | 0.405 | 0 | 0 | 0.203 | 0.505 |
| BRCA2 | CDC6 | 0.263 | 0 | 0 | 0.385 | 0.527 |
| BRCA2 | AURKA | 0.178 | 0.071 | 0 | 0.495 | 0.581 |
| BRCA2 | CCNA1 | 0.067 | 0.292 | 0 | 0.41 | 0.576 |
| BRCA2 | KIF11 | 0.365 | 0 | 0 | 0.189 | 0.463 |
| BRCA2 | CENPE | 0.334 | 0 | 0 | 0.382 | 0.571 |
| BRCA2 | MAD2L1 | 0.201 | 0 | 0 | 0.286 | 0.406 |
| BRCA2 | TPX2 | 0.217 | 0 | 0 | 0.431 | 0.535 |
| BRCA2 | RMI2 | 0.092 | 0 | 0.5 | 0.426 | 0.717 |
| BRCA2 | CDC25C | 0.207 | 0 | 0 | 0.37 | 0.479 |
| BRCA2 | KNL1 | 0.337 | 0.506 | 0 | 0.272 | 0.741 |
| BRCA2 | CENPF | 0.251 | 0 | 0 | 0.311 | 0.462 |
| BRCA2 | NEK2 | 0.196 | 0 | 0 | 0.338 | 0.445 |
| BRCA2 | CEP55 | 0.253 | 0 | 0 | 0.506 | 0.615 |
| BRCA2 | RAD54L | 0.219 | 0.073 | 0 | 0.723 | 0.782 |
| BRCA2 | KIF4A | 0.238 | 0.292 | 0 | 0.532 | 0.725 |
| BRCA2 | PSMC3IP | 0.128 | 0 | 0.4 | 0.243 | 0.569 |
| BRCA2 | UBE2T | 0.149 | 0 | 0 | 0.597 | 0.643 |
| BRCA2 | CDK1 | 0.268 | 0.046 | 0 | 0.532 | 0.645 |
| BRCA2 | EME1 | 0.144 | 0 | 0.5 | 0.549 | 0.79 |
| BRCA2 | CCNA2 | 0.259 | 0.292 | 0 | 0.784 | 0.877 |
| BRCA2 | HMMR | 0.256 | 0 | 0 | 0.909 | 0.929 |
| BRCA2 | RAD51 | 0.28 | 0.996 | 0.9 | 0.999 | 0.999 |
| CCNA1 | CDC6 | 0.401 | 0 | 0.9 | 0.907 | 0.993 |
| CCNA1 | AURKA | 0.295 | 0 | 0 | 0.492 | 0.627 |
| CCNA1 | ORC6 | 0.086 | 0 | 0.4 | 0.327 | 0.599 |
| CCNA1 | GMNN | 0.111 | 0 | 0 | 0.547 | 0.58 |
| CCNA1 | ECT2 | 0.294 | 0 | 0 | 0.191 | 0.405 |
| CCNA1 | CENPA | 0.139 | 0 | 0 | 0.349 | 0.415 |
| CCNA1 | NUF2 | 0.321 | 0 | 0 | 0.193 | 0.429 |
| CCNA1 | CENPF | 0.295 | 0 | 0 | 0.313 | 0.495 |
| CCNA1 | CENPE | 0.296 | 0.091 | 0 | 0.287 | 0.504 |
| CCNA1 | SGO1 | 0.401 | 0 | 0 | 0.215 | 0.51 |
| CCNA1 | RAD51 | 0.236 | 0 | 0 | 0.421 | 0.539 |
| CCNA1 | BRCA2 | 0.067 | 0.292 | 0 | 0.41 | 0.576 |
| CCNA1 | SPAG5 | 0.545 | 0 | 0 | 0.128 | 0.587 |
| CCNA1 | NEK2 | 0.298 | 0 | 0 | 0.534 | 0.659 |
| CCNA1 | PTTG1 | 0.251 | 0 | 0 | 0.61 | 0.695 |
| CCNA1 | KIF11 | 0.595 | 0 | 0 | 0.295 | 0.703 |
| CCNA1 | CDKN3 | 0.135 | 0 | 0 | 0.704 | 0.733 |
| CCNA1 | CCNB2 | 0.341 | 0.215 | 0 | 0.511 | 0.739 |
| CCNA1 | MAD2L1 | 0.358 | 0 | 0.5 | 0.282 | 0.749 |
| CCNA1 | CKS2 | 0.211 | 0.226 | 0 | 0.767 | 0.845 |
| CCNA1 | CDC25C | 0.189 | 0 | 0.5 | 0.724 | 0.878 |
| CCNA1 | CKS1B | 0.199 | 0.226 | 0.5 | 0.85 | 0.947 |
| CCNA1 | CCNA2 | 0.044 | 0.62 | 0.9 | 0.438 | 0.977 |
| CCNA1 | CDK1 | 0.297 | 0.803 | 0.9 | 0.998 | 0.999 |
| CCNA2 | CDC6 | 0.672 | 0.96 | 0.9 | 0.941 | 0.999 |
| CCNA2 | CENPM | 0.65 | 0 | 0 | 0.394 | 0.779 |
| CCNA2 | AURKA | 0.853 | 0 | 0 | 0.669 | 0.949 |
| CCNA2 | ORC6 | 0.251 | 0 | 0.4 | 0.429 | 0.721 |
| CCNA2 | GMNN | 0.427 | 0.68 | 0 | 0.41 | 0.882 |
| CCNA2 | KNSTRN | 0.587 | 0 | 0 | 0.112 | 0.618 |
| CCNA2 | PIMREG | 0.472 | 0 | 0 | 0.197 | 0.558 |
| CCNA2 | NCAPG | 0.854 | 0 | 0 | 0.668 | 0.949 |
| CCNA2 | CCNA1 | 0.044 | 0.62 | 0.9 | 0.438 | 0.977 |
| CCNA2 | TROAP | 0.601 | 0 | 0 | 0.185 | 0.661 |
| CCNA2 | KIF11 | 0.913 | 0 | 0 | 0.762 | 0.978 |
| CCNA2 | ASF1B | 0.605 | 0 | 0 | 0.473 | 0.783 |
| CCNA2 | SGO1 | 0.698 | 0 | 0 | 0.317 | 0.785 |
| CCNA2 | DEPDC1B | 0.67 | 0 | 0 | 0.085 | 0.685 |
| CCNA2 | CENPE | 0.837 | 0.091 | 0 | 0.488 | 0.917 |
| CCNA2 | NUF2 | 0.91 | 0 | 0 | 0.37 | 0.94 |
| CCNA2 | SPC25 | 0.805 | 0 | 0 | 0.068 | 0.811 |
| CCNA2 | CCNB2 | 0.856 | 0.215 | 0 | 0.696 | 0.965 |
| CCNA2 | MAD2L1 | 0.849 | 0 | 0.5 | 0.853 | 0.988 |
| CCNA2 | TPX2 | 0.86 | 0 | 0 | 0.691 | 0.954 |
| CCNA2 | RMI2 | 0.326 | 0 | 0 | 0.17 | 0.417 |
| CCNA2 | CKS1B | 0.489 | 0.976 | 0.5 | 0.905 | 0.999 |
| CCNA2 | SKA3 | 0.528 | 0 | 0 | 0.559 | 0.783 |
| CCNA2 | CDC25C | 0.688 | 0 | 0.5 | 0.741 | 0.956 |
| CCNA2 | SPAG5 | 0.828 | 0 | 0 | 0.451 | 0.901 |
| CCNA2 | NCAPD2 | 0.485 | 0 | 0 | 0.29 | 0.619 |
| CCNA2 | CDKN3 | 0.816 | 0 | 0 | 0.725 | 0.947 |
| CCNA2 | KNL1 | 0.406 | 0 | 0 | 0.227 | 0.522 |
| CCNA2 | CENPA | 0.982 | 0 | 0 | 0.482 | 0.99 |
| CCNA2 | CENPF | 0.832 | 0 | 0 | 0.68 | 0.944 |
| CCNA2 | NEK2 | 0.829 | 0 | 0 | 0.681 | 0.943 |
| CCNA2 | CEP55 | 0.822 | 0 | 0 | 0.586 | 0.923 |
| CCNA2 | RAD54L | 0.461 | 0 | 0 | 0.269 | 0.589 |
| CCNA2 | CENPI | 0.328 | 0 | 0 | 0.197 | 0.438 |
| CCNA2 | ZWINT | 0.774 | 0 | 0 | 0.58 | 0.901 |
| CCNA2 | KIF4A | 0.828 | 0 | 0 | 0.417 | 0.895 |
| CCNA2 | CKS2 | 0.518 | 0.85 | 0 | 0.872 | 0.99 |
| CCNA2 | BRCA2 | 0.259 | 0.292 | 0 | 0.784 | 0.877 |
| CCNA2 | ECT2 | 0.639 | 0 | 0 | 0.33 | 0.748 |
| CCNA2 | EME1 | 0.255 | 0.054 | 0 | 0.22 | 0.403 |
| CCNA2 | HMMR | 0.802 | 0 | 0 | 0.619 | 0.921 |
| CCNA2 | PTTG1 | 0.808 | 0 | 0 | 0.672 | 0.934 |
| CCNA2 | CDK1 | 0.993 | 0.939 | 0.9 | 0.998 | 0.999 |
| CCNA2 | CENPK | 0.529 | 0 | 0 | 0.17 | 0.592 |
| CCNA2 | DEPDC1 | 0.797 | 0 | 0 | 0.502 | 0.894 |
| CCNA2 | KPNA2 | 0.448 | 0.093 | 0 | 0.241 | 0.587 |
| CCNA2 | KIF18B | 0.461 | 0.091 | 0 | 0.273 | 0.613 |
| CCNA2 | TK1 | 0.482 | 0 | 0 | 0.593 | 0.78 |
| CCNA2 | BORA | 0.405 | 0 | 0 | 0.203 | 0.505 |
| CCNA2 | UBE2T | 0.474 | 0.101 | 0 | 0.294 | 0.637 |
| CCNA2 | RAD51 | 0.616 | 0 | 0 | 0.479 | 0.791 |
| CCNB2 | CDC6 | 0.465 | 0.423 | 0 | 0.885 | 0.961 |
| CCNB2 | CENPM | 0.575 | 0 | 0.4 | 0.595 | 0.887 |
| CCNB2 | AURKA | 0.86 | 0.637 | 0 | 0.799 | 0.988 |
| CCNB2 | ORC6 | 0.254 | 0 | 0 | 0.397 | 0.531 |
| CCNB2 | GMNN | 0.366 | 0 | 0 | 0.349 | 0.569 |
| CCNB2 | KNSTRN | 0.382 | 0 | 0 | 0.126 | 0.437 |
| CCNB2 | PIMREG | 0.465 | 0 | 0 | 0.559 | 0.754 |
| CCNB2 | NCAPG | 0.823 | 0 | 0.4 | 0.742 | 0.97 |
| CCNB2 | CCNA1 | 0.341 | 0.215 | 0 | 0.511 | 0.739 |
| CCNB2 | TROAP | 0.564 | 0 | 0 | 0.282 | 0.674 |
| CCNB2 | KIF11 | 0.841 | 0.136 | 0 | 0.629 | 0.944 |
| CCNB2 | PRR11 | 0.313 | 0 | 0 | 0.187 | 0.418 |
| CCNB2 | ASF1B | 0.477 | 0.094 | 0 | 0.199 | 0.587 |
| CCNB2 | SGO1 | 0.526 | 0.045 | 0.4 | 0.464 | 0.835 |
| CCNB2 | DEPDC1B | 0.55 | 0 | 0 | 0.168 | 0.61 |
| CCNB2 | CENPE | 0.595 | 0.051 | 0.4 | 0.78 | 0.942 |
| CCNB2 | NUF2 | 0.744 | 0 | 0.4 | 0.594 | 0.932 |
| CCNB2 | SPC25 | 0.493 | 0 | 0.4 | 0.207 | 0.738 |
| CCNB2 | MTFR2 | 0.248 | 0.1 | 0 | 0.191 | 0.405 |
| CCNB2 | KIF18B | 0.309 | 0.051 | 0 | 0.313 | 0.51 |
| CCNB2 | RAD54L | 0.4 | 0.094 | 0 | 0.345 | 0.613 |
| CCNB2 | BORA | 0.31 | 0 | 0.4 | 0.176 | 0.629 |
| CCNB2 | SKA3 | 0.568 | 0 | 0 | 0.225 | 0.651 |
| CCNB2 | UBE2T | 0.487 | 0.072 | 0 | 0.346 | 0.662 |
| CCNB2 | CENPK | 0.358 | 0 | 0.4 | 0.252 | 0.686 |
| CCNB2 | DEPDC1 | 0.615 | 0 | 0 | 0.314 | 0.724 |
| CCNB2 | NCAPD2 | 0.439 | 0 | 0.4 | 0.287 | 0.739 |
| CCNB2 | SPAG5 | 0.586 | 0.146 | 0 | 0.376 | 0.76 |
| CCNB2 | KPNA2 | 0.67 | 0.128 | 0 | 0.343 | 0.794 |
| CCNB2 | CENPI | 0.325 | 0 | 0.4 | 0.536 | 0.795 |
| CCNB2 | RAD51 | 0.522 | 0.189 | 0 | 0.552 | 0.811 |
| CCNB2 | ECT2 | 0.582 | 0.071 | 0 | 0.583 | 0.824 |
| CCNB2 | KNL1 | 0.398 | 0.117 | 0.4 | 0.527 | 0.829 |
| CCNB2 | KIF4A | 0.724 | 0 | 0 | 0.529 | 0.864 |
| CCNB2 | TK1 | 0.728 | 0 | 0 | 0.578 | 0.88 |
| CCNB2 | HMMR | 0.796 | 0.091 | 0 | 0.446 | 0.888 |
| CCNB2 | CDKN3 | 0.815 | 0.175 | 0 | 0.358 | 0.893 |
| CCNB2 | NEK2 | 0.67 | 0 | 0 | 0.717 | 0.902 |
| CCNB2 | CEP55 | 0.811 | 0 | 0 | 0.529 | 0.907 |
| CCNB2 | ZWINT | 0.539 | 0 | 0.4 | 0.735 | 0.92 |
| CCNB2 | CKS1B | 0.569 | 0.807 | 0 | 0.478 | 0.952 |
| CCNB2 | TPX2 | 0.835 | 0.045 | 0 | 0.725 | 0.953 |
| CCNB2 | MAD2L1 | 0.615 | 0.127 | 0.4 | 0.835 | 0.962 |
| CCNB2 | CCNA2 | 0.856 | 0.215 | 0 | 0.696 | 0.965 |
| CCNB2 | PTTG1 | 0.863 | 0 | 0 | 0.79 | 0.97 |
| CCNB2 | CENPF | 0.828 | 0 | 0.4 | 0.767 | 0.974 |
| CCNB2 | CENPA | 0.832 | 0.047 | 0.4 | 0.772 | 0.975 |
| CCNB2 | CKS2 | 0.56 | 0.797 | 0 | 0.759 | 0.976 |
| CCNB2 | CDC25C | 0.786 | 0.245 | 0.9 | 0.659 | 0.993 |
| CCNB2 | CDK1 | 0.899 | 0.882 | 0.9 | 0.996 | 0.999 |
| CDC25C | CDC6 | 0.386 | 0.099 | 0 | 0.698 | 0.818 |
| CDC25C | CENPM | 0.38 | 0 | 0 | 0.246 | 0.513 |
| CDC25C | AURKA | 0.645 | 0 | 0 | 0.544 | 0.831 |
| CDC25C | PIMREG | 0.473 | 0 | 0 | 0.258 | 0.593 |
| CDC25C | NCAPG | 0.48 | 0 | 0 | 0.194 | 0.563 |
| CDC25C | CCNA1 | 0.189 | 0 | 0.5 | 0.724 | 0.878 |
| CDC25C | TROAP | 0.553 | 0 | 0 | 0.265 | 0.657 |
| CDC25C | KIF11 | 0.51 | 0 | 0 | 0.335 | 0.66 |
| CDC25C | ASF1B | 0.368 | 0 | 0 | 0.091 | 0.401 |
| CDC25C | SGO1 | 0.462 | 0 | 0 | 0.191 | 0.546 |
| CDC25C | DEPDC1B | 0.45 | 0 | 0 | 0.097 | 0.482 |
| CDC25C | CENPE | 0.515 | 0.085 | 0 | 0.353 | 0.688 |
| CDC25C | NUF2 | 0.556 | 0 | 0 | 0.266 | 0.66 |
| CDC25C | SPC25 | 0.463 | 0 | 0 | 0.069 | 0.478 |
| CDC25C | CCNB2 | 0.786 | 0.245 | 0.9 | 0.659 | 0.993 |
| CDC25C | MAD2L1 | 0.396 | 0.075 | 0 | 0.406 | 0.639 |
| CDC25C | TPX2 | 0.541 | 0 | 0 | 0.256 | 0.644 |
| CDC25C | CKS1B | 0.294 | 0.133 | 0 | 0.278 | 0.519 |
| CDC25C | SKA3 | 0.513 | 0 | 0 | 0.189 | 0.588 |
| CDC25C | MTFR2 | 0.337 | 0.045 | 0 | 0.128 | 0.4 |
| CDC25C | KNL1 | 0.346 | 0 | 0 | 0.138 | 0.413 |
| CDC25C | NCAPD2 | 0.292 | 0 | 0 | 0.211 | 0.418 |
| CDC25C | EME1 | 0.273 | 0 | 0 | 0.242 | 0.426 |
| CDC25C | UBE2T | 0.343 | 0.048 | 0 | 0.164 | 0.432 |
| CDC25C | BORA | 0.241 | 0 | 0 | 0.288 | 0.437 |
| CDC25C | CENPI | 0.38 | 0 | 0 | 0.133 | 0.44 |
| CDC25C | TK1 | 0.361 | 0 | 0 | 0.164 | 0.443 |
| CDC25C | BRCA2 | 0.207 | 0 | 0 | 0.37 | 0.479 |
| CDC25C | ZWINT | 0.382 | 0 | 0 | 0.248 | 0.515 |
| CDC25C | ECT2 | 0.329 | 0.106 | 0 | 0.276 | 0.528 |
| CDC25C | KIF18B | 0.238 | 0.085 | 0 | 0.398 | 0.544 |
| CDC25C | RAD54L | 0.425 | 0.067 | 0 | 0.27 | 0.574 |
| CDC25C | CEP55 | 0.471 | 0 | 0 | 0.248 | 0.585 |
| CDC25C | CKS2 | 0.312 | 0.133 | 0 | 0.366 | 0.588 |
| CDC25C | RAD51 | 0.361 | 0 | 0 | 0.384 | 0.59 |
| CDC25C | SPAG5 | 0.464 | 0 | 0 | 0.287 | 0.602 |
| CDC25C | CENPF | 0.46 | 0 | 0 | 0.323 | 0.618 |
| CDC25C | PTTG1 | 0.48 | 0 | 0 | 0.477 | 0.716 |
| CDC25C | HMMR | 0.654 | 0 | 0 | 0.223 | 0.72 |
| CDC25C | CDKN3 | 0.53 | 0.146 | 0 | 0.381 | 0.73 |
| CDC25C | KIF4A | 0.648 | 0 | 0 | 0.45 | 0.798 |
| CDC25C | DEPDC1 | 0.77 | 0 | 0 | 0.201 | 0.808 |
| CDC25C | NEK2 | 0.652 | 0 | 0 | 0.486 | 0.814 |
| CDC25C | CENPA | 0.771 | 0 | 0 | 0.352 | 0.845 |
| CDC25C | CCNA2 | 0.688 | 0 | 0.5 | 0.741 | 0.956 |
| CDC25C | CDK1 | 0.775 | 0.714 | 0.9 | 0.975 | 0.999 |
| CDC6 | EME1 | 0.273 | 0 | 0 | 0.208 | 0.4 |
| CDC6 | CENPK | 0.362 | 0 | 0 | 0.109 | 0.408 |
| CDC6 | CENPI | 0.315 | 0 | 0 | 0.173 | 0.41 |
| CDC6 | PIMREG | 0.269 | 0 | 0 | 0.231 | 0.414 |
| CDC6 | KIF18B | 0.292 | 0 | 0 | 0.228 | 0.43 |
| CDC6 | DEPDC1B | 0.369 | 0 | 0 | 0.159 | 0.447 |
| CDC6 | SPC25 | 0.471 | 0 | 0 | 0 | 0.471 |
| CDC6 | CENPM | 0.378 | 0 | 0 | 0.197 | 0.479 |
| CDC6 | ECT2 | 0.349 | 0 | 0 | 0.287 | 0.516 |
| CDC6 | SGO1 | 0.38 | 0 | 0 | 0.252 | 0.516 |
| CDC6 | BRCA2 | 0.263 | 0 | 0 | 0.385 | 0.527 |
| CDC6 | KPNA2 | 0.367 | 0.14 | 0 | 0.209 | 0.532 |
| CDC6 | DEPDC1 | 0.452 | 0 | 0 | 0.189 | 0.537 |
| CDC6 | SKA3 | 0.465 | 0 | 0 | 0.174 | 0.539 |
| CDC6 | CENPE | 0.308 | 0 | 0 | 0.379 | 0.552 |
| CDC6 | UBE2T | 0.346 | 0.077 | 0 | 0.327 | 0.558 |
| CDC6 | SPAG5 | 0.48 | 0 | 0 | 0.193 | 0.562 |
| CDC6 | KIF4A | 0.427 | 0 | 0 | 0.268 | 0.563 |
| CDC6 | HMMR | 0.43 | 0 | 0 | 0.299 | 0.584 |
| CDC6 | RAD54L | 0.43 | 0.091 | 0 | 0.292 | 0.601 |
| CDC6 | CEP55 | 0.476 | 0 | 0 | 0.323 | 0.63 |
| CDC6 | CKS1B | 0.279 | 0.153 | 0 | 0.45 | 0.635 |
| CDC6 | CENPA | 0.346 | 0.131 | 0 | 0.418 | 0.64 |
| CDC6 | CENPF | 0.377 | 0 | 0 | 0.447 | 0.64 |
| CDC6 | CKS2 | 0.296 | 0.153 | 0 | 0.451 | 0.644 |
| CDC6 | ASF1B | 0.411 | 0 | 0 | 0.427 | 0.648 |
| CDC6 | NEK2 | 0.446 | 0 | 0 | 0.392 | 0.648 |
| CDC6 | NCAPG | 0.477 | 0 | 0 | 0.39 | 0.667 |
| CDC6 | PTTG1 | 0.331 | 0 | 0 | 0.528 | 0.671 |
| CDC6 | CDKN3 | 0.355 | 0.278 | 0 | 0.354 | 0.673 |
| CDC6 | TPX2 | 0.539 | 0 | 0 | 0.338 | 0.681 |
| CDC6 | NCAPD2 | 0.426 | 0 | 0 | 0.47 | 0.683 |
| CDC6 | TK1 | 0.542 | 0 | 0 | 0.348 | 0.689 |
| CDC6 | ZWINT | 0.63 | 0 | 0 | 0.248 | 0.71 |
| CDC6 | NUF2 | 0.566 | 0 | 0 | 0.374 | 0.717 |
| CDC6 | MAD2L1 | 0.501 | 0 | 0 | 0.528 | 0.754 |
| CDC6 | AURKA | 0.477 | 0.075 | 0 | 0.546 | 0.761 |
| CDC6 | CDC25C | 0.386 | 0.099 | 0 | 0.698 | 0.818 |
| CDC6 | RAD51 | 0.655 | 0 | 0 | 0.508 | 0.823 |
| CDC6 | KIF11 | 0.78 | 0 | 0 | 0.434 | 0.87 |
| CDC6 | GMNN | 0.382 | 0 | 0.5 | 0.717 | 0.905 |
| CDC6 | CCNB2 | 0.465 | 0.423 | 0 | 0.885 | 0.961 |
| CDC6 | CDK1 | 0.55 | 0.732 | 0 | 0.717 | 0.963 |
| CDC6 | CCNA1 | 0.401 | 0 | 0.9 | 0.907 | 0.993 |
| CDC6 | CCNA2 | 0.672 | 0.96 | 0.9 | 0.941 | 0.999 |
| CDC6 | ORC6 | 0.261 | 0.927 | 0.9 | 0.989 | 0.999 |
| CDK1 | CDC6 | 0.55 | 0.732 | 0 | 0.717 | 0.963 |
| CDK1 | CENPM | 0.675 | 0 | 0.4 | 0.299 | 0.851 |
| CDK1 | AURKA | 0.777 | 0.525 | 0 | 0.537 | 0.951 |
| CDK1 | ORC6 | 0.284 | 0 | 0.5 | 0.34 | 0.743 |
| CDK1 | GMNN | 0.52 | 0.623 | 0 | 0.325 | 0.867 |
| CDK1 | KNSTRN | 0.543 | 0 | 0 | 0.247 | 0.641 |
| CDK1 | PIMREG | 0.485 | 0 | 0 | 0.53 | 0.748 |
| CDK1 | NCAPG | 0.866 | 0.292 | 0.4 | 0.708 | 0.981 |
| CDK1 | CCNA1 | 0.297 | 0.803 | 0.9 | 0.998 | 0.999 |
| CDK1 | TROAP | 0.49 | 0 | 0 | 0.148 | 0.547 |
| CDK1 | KIF11 | 0.858 | 0.364 | 0 | 0.869 | 0.987 |
| CDK1 | ASF1B | 0.493 | 0.095 | 0 | 0.282 | 0.641 |
| CDK1 | SGO1 | 0.668 | 0.128 | 0.4 | 0.501 | 0.902 |
| CDK1 | DEPDC1B | 0.705 | 0.051 | 0 | 0.217 | 0.762 |
| CDK1 | CENPE | 0.835 | 0.097 | 0.4 | 0.741 | 0.973 |
| CDK1 | NUF2 | 0.867 | 0 | 0.4 | 0.51 | 0.957 |
| CDK1 | SPC25 | 0.832 | 0 | 0.4 | 0.046 | 0.895 |
| CDK1 | CCNB2 | 0.899 | 0.882 | 0.9 | 0.996 | 0.999 |
| CDK1 | MAD2L1 | 0.857 | 0.113 | 0.5 | 0.896 | 0.992 |
| CDK1 | TPX2 | 0.825 | 0.045 | 0 | 0.736 | 0.952 |
| CDK1 | RMI2 | 0.324 | 0 | 0 | 0.235 | 0.461 |
| CDK1 | CKS1B | 0.536 | 0.994 | 0 | 0.978 | 0.999 |
| CDK1 | SKA3 | 0.681 | 0 | 0 | 0.402 | 0.801 |
| CDK1 | CDC25C | 0.775 | 0.714 | 0.9 | 0.975 | 0.999 |
| CDK1 | SPAG5 | 0.761 | 0.354 | 0 | 0.566 | 0.927 |
| CDK1 | NCAPD2 | 0.298 | 0.292 | 0.4 | 0.375 | 0.789 |
| CDK1 | CDKN3 | 0.828 | 0.706 | 0 | 0.688 | 0.982 |
| CDK1 | KNL1 | 0.479 | 0.068 | 0.4 | 0.512 | 0.838 |
| CDK1 | CENPA | 0.961 | 0.071 | 0.4 | 0.592 | 0.989 |
| CDK1 | CENPF | 0.825 | 0.292 | 0.4 | 0.745 | 0.978 |
| CDK1 | NEK2 | 0.832 | 0 | 0 | 0.493 | 0.919 |
| CDK1 | CEP55 | 0.829 | 0 | 0 | 0.759 | 0.957 |
| CDK1 | RAD54L | 0.438 | 0.107 | 0 | 0.596 | 0.78 |
| CDK1 | CENPI | 0.353 | 0.071 | 0.4 | 0.58 | 0.828 |
| CDK1 | ZWINT | 0.79 | 0 | 0.4 | 0.638 | 0.95 |
| CDK1 | KIF4A | 0.82 | 0 | 0 | 0.527 | 0.911 |
| CDK1 | CKS2 | 0.689 | 0.992 | 0 | 0.967 | 0.999 |
| CDK1 | BRCA2 | 0.268 | 0.046 | 0 | 0.532 | 0.645 |
| CDK1 | ECT2 | 0.774 | 0.071 | 0 | 0.838 | 0.963 |
| CDK1 | EME1 | 0.269 | 0.045 | 0 | 0.543 | 0.653 |
| CDK1 | HMMR | 0.829 | 0.094 | 0 | 0.59 | 0.931 |
| CDK1 | PTTG1 | 0.856 | 0 | 0 | 0.902 | 0.985 |
| CDK1 | MTFR2 | 0.348 | 0.09 | 0 | 0.1 | 0.42 |
| CDK1 | KIF18B | 0.321 | 0.096 | 0 | 0.306 | 0.537 |
| CDK1 | CENPK | 0.565 | 0 | 0.4 | 0.256 | 0.789 |
| CDK1 | RAD51 | 0.6 | 0.071 | 0 | 0.596 | 0.837 |
| CDK1 | TK1 | 0.576 | 0 | 0 | 0.638 | 0.84 |
| CDK1 | UBE2T | 0.783 | 0.091 | 0 | 0.275 | 0.844 |
| CDK1 | KPNA2 | 0.634 | 0.097 | 0 | 0.577 | 0.848 |
| CDK1 | DEPDC1 | 0.79 | 0.051 | 0 | 0.305 | 0.849 |
| CDK1 | BORA | 0.373 | 0 | 0.5 | 0.74 | 0.911 |
| CDK1 | CCNA2 | 0.993 | 0.939 | 0.9 | 0.998 | 0.999 |
| CDKN3 | CDC6 | 0.355 | 0.278 | 0 | 0.354 | 0.673 |
| CDKN3 | CENPM | 0.647 | 0 | 0 | 0.069 | 0.657 |
| CDKN3 | AURKA | 0.793 | 0.135 | 0 | 0.334 | 0.87 |
| CDKN3 | KNSTRN | 0.452 | 0 | 0 | 0 | 0.452 |
| CDKN3 | PIMREG | 0.464 | 0 | 0 | 0.086 | 0.489 |
| CDKN3 | NCAPG | 0.792 | 0 | 0 | 0.15 | 0.816 |
| CDKN3 | CCNA1 | 0.135 | 0 | 0 | 0.704 | 0.733 |
| CDKN3 | TROAP | 0.47 | 0 | 0 | 0 | 0.47 |
| CDKN3 | KIF11 | 0.794 | 0.096 | 0 | 0.25 | 0.848 |
| CDKN3 | DEPDC1B | 0.479 | 0 | 0 | 0.053 | 0.485 |
| CDKN3 | CENPE | 0.784 | 0.072 | 0 | 0.122 | 0.808 |
| CDKN3 | NUF2 | 0.811 | 0.064 | 0 | 0.264 | 0.858 |
| CDKN3 | SPC25 | 0.78 | 0 | 0 | 0.2 | 0.817 |
| CDKN3 | CCNB2 | 0.815 | 0.175 | 0 | 0.358 | 0.893 |
| CDKN3 | MAD2L1 | 0.78 | 0.226 | 0 | 0.297 | 0.87 |
| CDKN3 | TPX2 | 0.787 | 0.044 | 0 | 0.178 | 0.818 |
| CDKN3 | CKS1B | 0.538 | 0.046 | 0 | 0.246 | 0.638 |
| CDKN3 | SKA3 | 0.462 | 0 | 0 | 0.046 | 0.465 |
| CDKN3 | CDC25C | 0.53 | 0.146 | 0 | 0.381 | 0.73 |
| CDKN3 | SPAG5 | 0.524 | 0.089 | 0 | 0.117 | 0.584 |
| CDKN3 | ECT2 | 0.395 | 0.071 | 0 | 0.118 | 0.462 |
| CDKN3 | RAD51 | 0.346 | 0 | 0 | 0.215 | 0.465 |
| CDKN3 | TK1 | 0.486 | 0 | 0 | 0.205 | 0.574 |
| CDKN3 | ZWINT | 0.639 | 0 | 0 | 0.138 | 0.676 |
| CDKN3 | NEK2 | 0.67 | 0 | 0 | 0.191 | 0.722 |
| CDKN3 | CKS2 | 0.665 | 0.046 | 0 | 0.21 | 0.725 |
| CDKN3 | CENPF | 0.666 | 0 | 0 | 0.21 | 0.725 |
| CDKN3 | DEPDC1 | 0.725 | 0 | 0 | 0.056 | 0.73 |
| CDKN3 | UBE2T | 0.78 | 0.047 | 0 | 0.105 | 0.796 |
| CDKN3 | KIF4A | 0.787 | 0 | 0 | 0.111 | 0.803 |
| CDKN3 | CEP55 | 0.798 | 0 | 0 | 0.154 | 0.822 |
| CDKN3 | CENPA | 0.791 | 0.091 | 0 | 0.145 | 0.824 |
| CDKN3 | HMMR | 0.801 | 0.089 | 0 | 0.154 | 0.833 |
| CDKN3 | PTTG1 | 0.8 | 0 | 0 | 0.23 | 0.84 |
| CDKN3 | CCNA2 | 0.816 | 0 | 0 | 0.725 | 0.947 |
| CDKN3 | CDK1 | 0.828 | 0.706 | 0 | 0.688 | 0.982 |
| CENPA | CDC6 | 0.346 | 0.131 | 0 | 0.418 | 0.64 |
| CENPA | CENPM | 0.633 | 0.897 | 0.5 | 0.956 | 0.999 |
| CENPA | AURKA | 0.785 | 0.149 | 0 | 0.797 | 0.959 |
| CENPA | ORC6 | 0.185 | 0 | 0 | 0.345 | 0.444 |
| CENPA | KNSTRN | 0.444 | 0 | 0 | 0.287 | 0.587 |
| CENPA | PIMREG | 0.783 | 0 | 0 | 0.395 | 0.863 |
| CENPA | NCAPG | 0.743 | 0.092 | 0 | 0.487 | 0.87 |
| CENPA | CCNA1 | 0.139 | 0 | 0 | 0.349 | 0.415 |
| CENPA | TROAP | 0.586 | 0 | 0 | 0.203 | 0.656 |
| CENPA | KIF11 | 0.832 | 0.078 | 0 | 0.658 | 0.942 |
| CENPA | ASF1B | 0.393 | 0.377 | 0 | 0.588 | 0.831 |
| CENPA | SGO1 | 0.574 | 0.042 | 0 | 0.73 | 0.88 |
| CENPA | DEPDC1B | 0.563 | 0 | 0 | 0.116 | 0.597 |
| CENPA | CENPE | 0.792 | 0.052 | 0 | 0.967 | 0.993 |
| CENPA | NUF2 | 0.618 | 0.058 | 0 | 0.836 | 0.936 |
| CENPA | SPC25 | 0.779 | 0 | 0 | 0.148 | 0.804 |
| CENPA | CCNB2 | 0.832 | 0.047 | 0.4 | 0.772 | 0.975 |
| CENPA | MAD2L1 | 0.521 | 0.085 | 0.5 | 0.457 | 0.865 |
| CENPA | TPX2 | 0.791 | 0 | 0 | 0.46 | 0.882 |
| CENPA | CKS1B | 0.385 | 0 | 0 | 0.284 | 0.541 |
| CENPA | SKA3 | 0.47 | 0 | 0 | 0.38 | 0.657 |
| CENPA | CDC25C | 0.771 | 0 | 0 | 0.352 | 0.845 |
| CENPA | SPAG5 | 0.746 | 0.094 | 0 | 0.3 | 0.825 |
| CENPA | NCAPD2 | 0.291 | 0 | 0 | 0.385 | 0.546 |
| CENPA | CDKN3 | 0.791 | 0.091 | 0 | 0.145 | 0.824 |
| CENPA | KNL1 | 0.352 | 0.084 | 0.5 | 0.712 | 0.903 |
| CENPA | KPNA2 | 0.278 | 0.087 | 0 | 0.183 | 0.415 |
| CENPA | UBE2T | 0.362 | 0.116 | 0 | 0.159 | 0.484 |
| CENPA | MTFR2 | 0.337 | 0.073 | 0 | 0.231 | 0.486 |
| CENPA | TK1 | 0.407 | 0 | 0 | 0.247 | 0.534 |
| CENPA | RAD54L | 0.361 | 0.15 | 0 | 0.239 | 0.551 |
| CENPA | CKS2 | 0.419 | 0 | 0 | 0.348 | 0.605 |
| CENPA | BORA | 0.277 | 0 | 0 | 0.479 | 0.607 |
| CENPA | KIF18B | 0.331 | 0.135 | 0 | 0.4 | 0.622 |
| CENPA | ECT2 | 0.44 | 0 | 0 | 0.376 | 0.635 |
| CENPA | RAD51 | 0.36 | 0.096 | 0 | 0.609 | 0.754 |
| CENPA | PTTG1 | 0.59 | 0 | 0 | 0.44 | 0.761 |
| CENPA | NEK2 | 0.691 | 0 | 0 | 0.535 | 0.85 |
| CENPA | DEPDC1 | 0.785 | 0 | 0 | 0.37 | 0.858 |
| CENPA | HMMR | 0.799 | 0.078 | 0 | 0.359 | 0.87 |
| CENPA | KIF4A | 0.799 | 0.125 | 0 | 0.51 | 0.906 |
| CENPA | ZWINT | 0.5 | 0 | 0 | 0.825 | 0.908 |
| CENPA | CEP55 | 0.803 | 0 | 0 | 0.613 | 0.92 |
| CENPA | CENPF | 0.656 | 0 | 0 | 0.903 | 0.965 |
| CENPA | CDK1 | 0.961 | 0.071 | 0.4 | 0.592 | 0.989 |
| CENPA | CCNA2 | 0.982 | 0 | 0 | 0.482 | 0.99 |
| CENPA | CENPK | 0.288 | 0.851 | 0.5 | 0.96 | 0.997 |
| CENPA | CENPI | 0.238 | 0.881 | 0.5 | 0.987 | 0.999 |
| CENPE | CDC6 | 0.308 | 0 | 0 | 0.379 | 0.552 |
| CENPE | CENPM | 0.305 | 0 | 0 | 0.621 | 0.725 |
| CENPE | AURKA | 0.643 | 0.128 | 0 | 0.613 | 0.869 |
| CENPE | GMNN | 0.234 | 0.114 | 0 | 0.298 | 0.482 |
| CENPE | KNSTRN | 0.453 | 0 | 0 | 0.807 | 0.89 |
| CENPE | PIMREG | 0.385 | 0 | 0 | 0.347 | 0.582 |
| CENPE | NCAPG | 0.859 | 0 | 0 | 0.459 | 0.92 |
| CENPE | CCNA1 | 0.296 | 0.091 | 0 | 0.287 | 0.504 |
| CENPE | TROAP | 0.522 | 0 | 0 | 0.146 | 0.575 |
| CENPE | KIF11 | 0.958 | 0.305 | 0.4 | 0.419 | 0.988 |
| CENPE | ASF1B | 0.282 | 0 | 0 | 0.402 | 0.552 |
| CENPE | SGO1 | 0.566 | 0.091 | 0 | 0.61 | 0.833 |
| CENPE | DEPDC1B | 0.575 | 0 | 0 | 0.261 | 0.672 |
| CENPE | UBE2T | 0.344 | 0.045 | 0 | 0.123 | 0.403 |
| CENPE | TK1 | 0.365 | 0 | 0 | 0.151 | 0.438 |
| CENPE | RAD54L | 0.35 | 0.066 | 0 | 0.186 | 0.463 |
| CENPE | BORA | 0.385 | 0 | 0 | 0.217 | 0.498 |
| CENPE | BRCA2 | 0.334 | 0 | 0 | 0.382 | 0.571 |
| CENPE | CKS2 | 0.405 | 0 | 0 | 0.324 | 0.581 |
| CENPE | NCAPD2 | 0.315 | 0 | 0 | 0.416 | 0.583 |
| CENPE | RAD51 | 0.376 | 0 | 0 | 0.467 | 0.653 |
| CENPE | SPC25 | 0.599 | 0 | 0 | 0.191 | 0.662 |
| CENPE | KIF18B | 0.386 | 0 | 0.4 | 0.221 | 0.688 |
| CENPE | CDC25C | 0.515 | 0.085 | 0 | 0.353 | 0.688 |
| CENPE | CENPK | 0.374 | 0 | 0 | 0.545 | 0.703 |
| CENPE | PTTG1 | 0.505 | 0 | 0 | 0.606 | 0.796 |
| CENPE | CDKN3 | 0.784 | 0.072 | 0 | 0.122 | 0.808 |
| CENPE | SPAG5 | 0.664 | 0.091 | 0 | 0.431 | 0.811 |
| CENPE | DEPDC1 | 0.793 | 0 | 0 | 0.201 | 0.828 |
| CENPE | NEK2 | 0.586 | 0 | 0 | 0.616 | 0.834 |
| CENPE | ECT2 | 0.62 | 0 | 0 | 0.605 | 0.844 |
| CENPE | SKA3 | 0.556 | 0 | 0 | 0.679 | 0.851 |
| CENPE | ZWINT | 0.488 | 0.087 | 0 | 0.76 | 0.878 |
| CENPE | CENPI | 0.381 | 0.114 | 0 | 0.803 | 0.882 |
| CENPE | TPX2 | 0.804 | 0 | 0 | 0.48 | 0.894 |
| CENPE | KNL1 | 0.466 | 0 | 0 | 0.82 | 0.9 |
| CENPE | CCNA2 | 0.837 | 0.091 | 0 | 0.488 | 0.917 |
| CENPE | HMMR | 0.807 | 0.057 | 0 | 0.625 | 0.926 |
| CENPE | CEP55 | 0.838 | 0 | 0 | 0.621 | 0.936 |
| CENPE | MAD2L1 | 0.45 | 0.222 | 0.5 | 0.762 | 0.942 |
| CENPE | CCNB2 | 0.595 | 0.051 | 0.4 | 0.78 | 0.942 |
| CENPE | KIF4A | 0.839 | 0 | 0.4 | 0.589 | 0.957 |
| CENPE | CDK1 | 0.835 | 0.097 | 0.4 | 0.741 | 0.973 |
| CENPE | CENPA | 0.792 | 0.052 | 0 | 0.967 | 0.993 |
| CENPE | NUF2 | 0.839 | 0.484 | 0 | 0.958 | 0.996 |
| CENPE | CENPF | 0.926 | 0.23 | 0 | 0.986 | 0.999 |
| CENPF | CDC6 | 0.377 | 0 | 0 | 0.447 | 0.64 |
| CENPF | CENPM | 0.353 | 0 | 0 | 0.687 | 0.789 |
| CENPF | AURKA | 0.679 | 0 | 0 | 0.708 | 0.902 |
| CENPF | ORC6 | 0.234 | 0 | 0 | 0.413 | 0.531 |
| CENPF | GMNN | 0.254 | 0 | 0 | 0.348 | 0.492 |
| CENPF | KNSTRN | 0.411 | 0 | 0 | 0.385 | 0.623 |
| CENPF | PIMREG | 0.436 | 0 | 0 | 0.445 | 0.673 |
| CENPF | NCAPG | 0.827 | 0 | 0 | 0.582 | 0.924 |
| CENPF | CCNA1 | 0.295 | 0 | 0 | 0.313 | 0.495 |
| CENPF | TROAP | 0.669 | 0 | 0 | 0.315 | 0.764 |
| CENPF | KIF11 | 0.903 | 0 | 0 | 0.728 | 0.972 |
| CENPF | ASF1B | 0.357 | 0 | 0 | 0.592 | 0.726 |
| CENPF | SGO1 | 0.58 | 0 | 0 | 0.419 | 0.746 |
| CENPF | DEPDC1B | 0.519 | 0 | 0 | 0.069 | 0.533 |
| CENPF | CENPE | 0.926 | 0.23 | 0 | 0.986 | 0.999 |
| CENPF | NUF2 | 0.875 | 0 | 0 | 0.632 | 0.952 |
| CENPF | SPC25 | 0.668 | 0 | 0 | 0.232 | 0.734 |
| CENPF | CCNB2 | 0.828 | 0 | 0.4 | 0.767 | 0.974 |
| CENPF | MAD2L1 | 0.657 | 0 | 0.5 | 0.544 | 0.915 |
| CENPF | TPX2 | 0.829 | 0 | 0 | 0.746 | 0.954 |
| CENPF | CKS1B | 0.351 | 0 | 0 | 0.394 | 0.591 |
| CENPF | SKA3 | 0.461 | 0 | 0.9 | 0.458 | 0.968 |
| CENPF | CDC25C | 0.46 | 0 | 0 | 0.323 | 0.618 |
| CENPF | SPAG5 | 0.838 | 0.045 | 0 | 0.436 | 0.905 |
| CENPF | NCAPD2 | 0.384 | 0 | 0 | 0.375 | 0.599 |
| CENPF | CDKN3 | 0.666 | 0 | 0 | 0.21 | 0.725 |
| CENPF | KNL1 | 0.498 | 0 | 0 | 0.588 | 0.784 |
| CENPF | CENPA | 0.656 | 0 | 0 | 0.903 | 0.965 |
| CENPF | BRCA2 | 0.251 | 0 | 0 | 0.311 | 0.462 |
| CENPF | KPNA2 | 0.362 | 0 | 0 | 0.222 | 0.483 |
| CENPF | RAD51 | 0.372 | 0 | 0 | 0.329 | 0.561 |
| CENPF | RAD54L | 0.382 | 0 | 0 | 0.382 | 0.602 |
| CENPF | CENPK | 0.326 | 0 | 0 | 0.494 | 0.645 |
| CENPF | CKS2 | 0.367 | 0 | 0 | 0.504 | 0.673 |
| CENPF | KIF18B | 0.524 | 0 | 0 | 0.392 | 0.698 |
| CENPF | ECT2 | 0.614 | 0 | 0 | 0.429 | 0.77 |
| CENPF | TK1 | 0.434 | 0 | 0 | 0.63 | 0.782 |
| CENPF | PTTG1 | 0.574 | 0 | 0 | 0.611 | 0.827 |
| CENPF | DEPDC1 | 0.754 | 0 | 0 | 0.392 | 0.844 |
| CENPF | UBE2T | 0.788 | 0 | 0 | 0.333 | 0.853 |
| CENPF | KIF4A | 0.73 | 0 | 0 | 0.571 | 0.879 |
| CENPF | CENPI | 0.373 | 0 | 0 | 0.863 | 0.91 |
| CENPF | HMMR | 0.787 | 0.045 | 0 | 0.669 | 0.927 |
| CENPF | CCNA2 | 0.832 | 0 | 0 | 0.68 | 0.944 |
| CENPF | ZWINT | 0.638 | 0 | 0 | 0.877 | 0.953 |
| CENPF | CEP55 | 0.847 | 0 | 0 | 0.745 | 0.959 |
| CENPF | NEK2 | 0.882 | 0 | 0 | 0.759 | 0.97 |
| CENPF | CDK1 | 0.825 | 0.292 | 0.4 | 0.745 | 0.978 |
| CENPI | CDC6 | 0.315 | 0 | 0 | 0.173 | 0.41 |
| CENPI | CENPM | 0.317 | 0.966 | 0.8 | 0.995 | 0.999 |
| CENPI | AURKA | 0.337 | 0.07 | 0 | 0.505 | 0.668 |
| CENPI | KNSTRN | 0.279 | 0 | 0 | 0.307 | 0.479 |
| CENPI | NCAPG | 0.38 | 0.113 | 0 | 0.471 | 0.684 |
| CENPI | KIF11 | 0.523 | 0.438 | 0 | 0.344 | 0.809 |
| CENPI | SGO1 | 0.615 | 0.099 | 0 | 0.617 | 0.856 |
| CENPI | DEPDC1B | 0.299 | 0 | 0 | 0.354 | 0.528 |
| CENPI | CENPE | 0.381 | 0.114 | 0 | 0.803 | 0.882 |
| CENPI | NUF2 | 0.454 | 0.078 | 0 | 0.787 | 0.883 |
| CENPI | SPC25 | 0.372 | 0 | 0 | 0.135 | 0.433 |
| CENPI | CCNB2 | 0.325 | 0 | 0.4 | 0.536 | 0.795 |
| CENPI | MAD2L1 | 0.44 | 0.306 | 0.5 | 0.549 | 0.9 |
| CENPI | TPX2 | 0.406 | 0.045 | 0 | 0.246 | 0.535 |
| CENPI | SKA3 | 0.499 | 0 | 0 | 0.248 | 0.607 |
| CENPI | CDC25C | 0.38 | 0 | 0 | 0.133 | 0.44 |
| CENPI | SPAG5 | 0.275 | 0.128 | 0 | 0.289 | 0.512 |
| CENPI | KNL1 | 0.433 | 0 | 0.5 | 0.671 | 0.898 |
| CENPI | CENPA | 0.238 | 0.881 | 0.5 | 0.987 | 0.999 |
| CENPI | CENPF | 0.373 | 0 | 0 | 0.863 | 0.91 |
| CENPI | NEK2 | 0.426 | 0 | 0 | 0.329 | 0.599 |
| CENPI | CEP55 | 0.451 | 0 | 0 | 0.384 | 0.647 |
| CENPI | RAD54L | 0.268 | 0.131 | 0 | 0.219 | 0.46 |
| CENPI | CCNA2 | 0.328 | 0 | 0 | 0.197 | 0.438 |
| CENPI | HMMR | 0.314 | 0.128 | 0 | 0.147 | 0.446 |
| CENPI | ECT2 | 0.343 | 0 | 0 | 0.21 | 0.459 |
| CENPI | DEPDC1 | 0.393 | 0 | 0 | 0.16 | 0.469 |
| CENPI | TONSL | 0.241 | 0.321 | 0 | 0.08 | 0.485 |
| CENPI | RAD51 | 0.399 | 0 | 0 | 0.179 | 0.486 |
| CENPI | KIF18B | 0.316 | 0.114 | 0 | 0.399 | 0.604 |
| CENPI | KIF4A | 0.542 | 0 | 0 | 0.278 | 0.655 |
| CENPI | ZWINT | 0.197 | 0 | 0 | 0.654 | 0.71 |
| CENPI | CDK1 | 0.353 | 0.071 | 0.4 | 0.58 | 0.828 |
| CENPI | CENPK | 0.215 | 0.969 | 0.8 | 0.999 | 0.999 |
| CENPK | CDC6 | 0.362 | 0 | 0 | 0.109 | 0.408 |
| CENPK | CENPM | 0.257 | 0.968 | 0.8 | 0.998 | 0.999 |
| CENPK | AURKA | 0.279 | 0.092 | 0 | 0.259 | 0.473 |
| CENPK | GMNN | 0.289 | 0 | 0 | 0.283 | 0.468 |
| CENPK | KNSTRN | 0.231 | 0 | 0 | 0.339 | 0.47 |
| CENPK | NCAPG | 0.455 | 0 | 0 | 0.381 | 0.649 |
| CENPK | KIF11 | 0.503 | 0 | 0 | 0.335 | 0.656 |
| CENPK | SGO1 | 0.346 | 0.143 | 0 | 0.245 | 0.54 |
| CENPK | DEPDC1B | 0.378 | 0 | 0 | 0.122 | 0.431 |
| CENPK | CENPE | 0.374 | 0 | 0 | 0.545 | 0.703 |
| CENPK | NUF2 | 0.594 | 0 | 0 | 0.511 | 0.793 |
| CENPK | SPC25 | 0.593 | 0 | 0 | 0.175 | 0.649 |
| CENPK | CCNB2 | 0.358 | 0 | 0.4 | 0.252 | 0.686 |
| CENPK | MAD2L1 | 0.618 | 0 | 0.5 | 0.268 | 0.848 |
| CENPK | TPX2 | 0.367 | 0 | 0 | 0.284 | 0.528 |
| CENPK | SKA3 | 0.374 | 0.137 | 0 | 0.29 | 0.583 |
| CENPK | NCAPD2 | 0.321 | 0.091 | 0 | 0.157 | 0.434 |
| CENPK | KNL1 | 0.326 | 0 | 0.5 | 0.602 | 0.854 |
| CENPK | CENPA | 0.288 | 0.851 | 0.5 | 0.96 | 0.997 |
| CENPK | CENPF | 0.326 | 0 | 0 | 0.494 | 0.645 |
| CENPK | NEK2 | 0.323 | 0 | 0 | 0.248 | 0.469 |
| CENPK | CEP55 | 0.385 | 0 | 0 | 0.292 | 0.547 |
| CENPK | RAD54L | 0.284 | 0.066 | 0 | 0.196 | 0.415 |
| CENPK | CENPI | 0.215 | 0.969 | 0.8 | 0.999 | 0.999 |
| CENPK | ZWINT | 0.305 | 0.171 | 0 | 0.436 | 0.646 |
| CENPK | KIF4A | 0.336 | 0 | 0 | 0.249 | 0.48 |
| CENPK | ECT2 | 0.421 | 0 | 0 | 0.321 | 0.591 |
| CENPK | HMMR | 0.383 | 0 | 0 | 0.184 | 0.475 |
| CENPK | PTTG1 | 0.365 | 0.124 | 0 | 0.251 | 0.547 |
| CENPK | CDK1 | 0.565 | 0 | 0.4 | 0.256 | 0.789 |
| CENPK | TK1 | 0.304 | 0 | 0 | 0.188 | 0.41 |
| CENPK | UBE2T | 0.371 | 0 | 0 | 0.113 | 0.419 |
| CENPK | RAD51 | 0.448 | 0 | 0 | 0 | 0.448 |
| CENPK | DEPDC1 | 0.307 | 0 | 0 | 0.254 | 0.461 |
| CENPK | CCNA2 | 0.529 | 0 | 0 | 0.17 | 0.592 |
| CENPM | CDC6 | 0.378 | 0 | 0 | 0.197 | 0.479 |
| CENPM | CKS1B | 0.303 | 0 | 0 | 0.178 | 0.403 |
| CENPM | KIF18B | 0.264 | 0 | 0 | 0.255 | 0.428 |
| CENPM | DEPDC1B | 0.295 | 0 | 0 | 0.245 | 0.446 |
| CENPM | TROAP | 0.369 | 0 | 0 | 0.16 | 0.448 |
| CENPM | CKS2 | 0.302 | 0 | 0 | 0.245 | 0.45 |
| CENPM | UBE2T | 0.409 | 0 | 0 | 0.108 | 0.451 |
| CENPM | SKA3 | 0.304 | 0.097 | 0 | 0.197 | 0.451 |
| CENPM | SGO1 | 0.304 | 0.131 | 0 | 0.22 | 0.487 |
| CENPM | SPAG5 | 0.358 | 0 | 0 | 0.248 | 0.497 |
| CENPM | AURKA | 0.342 | 0 | 0 | 0.269 | 0.499 |
| CENPM | ORC6 | 0.162 | 0 | 0 | 0.435 | 0.506 |
| CENPM | CDC25C | 0.38 | 0 | 0 | 0.246 | 0.513 |
| CENPM | PIMREG | 0.409 | 0 | 0 | 0.238 | 0.53 |
| CENPM | RAD51 | 0.424 | 0 | 0 | 0.249 | 0.549 |
| CENPM | NEK2 | 0.468 | 0 | 0 | 0.201 | 0.557 |
| CENPM | SPC25 | 0.544 | 0 | 0 | 0.159 | 0.6 |
| CENPM | HMMR | 0.475 | 0 | 0 | 0.302 | 0.618 |
| CENPM | NCAPG | 0.55 | 0 | 0 | 0.219 | 0.634 |
| CENPM | TPX2 | 0.478 | 0 | 0 | 0.333 | 0.637 |
| CENPM | CDKN3 | 0.647 | 0 | 0 | 0.069 | 0.657 |
| CENPM | PTTG1 | 0.498 | 0 | 0 | 0.37 | 0.67 |
| CENPM | RAD54L | 0.458 | 0 | 0 | 0.452 | 0.69 |
| CENPM | ASF1B | 0.409 | 0 | 0 | 0.499 | 0.692 |
| CENPM | NUF2 | 0.39 | 0 | 0 | 0.526 | 0.698 |
| CENPM | CENPE | 0.305 | 0 | 0 | 0.621 | 0.725 |
| CENPM | TK1 | 0.547 | 0 | 0 | 0.451 | 0.74 |
| CENPM | KIF4A | 0.547 | 0 | 0 | 0.451 | 0.741 |
| CENPM | CEP55 | 0.576 | 0 | 0 | 0.485 | 0.772 |
| CENPM | CCNA2 | 0.65 | 0 | 0 | 0.394 | 0.779 |
| CENPM | CENPF | 0.353 | 0 | 0 | 0.687 | 0.789 |
| CENPM | KIF11 | 0.512 | 0 | 0 | 0.606 | 0.799 |
| CENPM | KNL1 | 0.259 | 0 | 0.5 | 0.528 | 0.809 |
| CENPM | ZWINT | 0.655 | 0.169 | 0 | 0.402 | 0.814 |
| CENPM | MAD2L1 | 0.541 | 0 | 0.5 | 0.27 | 0.818 |
| CENPM | CDK1 | 0.675 | 0 | 0.4 | 0.299 | 0.851 |
| CENPM | CCNB2 | 0.575 | 0 | 0.4 | 0.595 | 0.887 |
| CENPM | CENPA | 0.633 | 0.897 | 0.5 | 0.956 | 0.999 |
| CENPM | CENPK | 0.257 | 0.968 | 0.8 | 0.998 | 0.999 |
| CENPM | CENPI | 0.317 | 0.966 | 0.8 | 0.995 | 0.999 |
| CEP55 | CDC6 | 0.476 | 0 | 0 | 0.323 | 0.63 |
| CEP55 | CENPM | 0.576 | 0 | 0 | 0.485 | 0.772 |
| CEP55 | AURKA | 0.802 | 0.161 | 0 | 0.65 | 0.936 |
| CEP55 | ORC6 | 0.203 | 0 | 0 | 0.394 | 0.496 |
| CEP55 | KNSTRN | 0.465 | 0 | 0 | 0.477 | 0.708 |
| CEP55 | PIMREG | 0.511 | 0 | 0 | 0.383 | 0.685 |
| CEP55 | NCAPG | 0.825 | 0 | 0 | 0.447 | 0.899 |
| CEP55 | TROAP | 0.487 | 0 | 0 | 0.274 | 0.612 |
| CEP55 | KIF11 | 0.994 | 0 | 0 | 0.519 | 0.997 |
| CEP55 | PRR11 | 0.24 | 0 | 0 | 0.251 | 0.406 |
| CEP55 | ASF1B | 0.37 | 0 | 0 | 0.434 | 0.628 |
| CEP55 | SGO1 | 0.623 | 0 | 0 | 0.355 | 0.746 |
| CEP55 | DEPDC1B | 0.789 | 0 | 0 | 0.249 | 0.834 |
| CEP55 | CENPE | 0.838 | 0 | 0 | 0.621 | 0.936 |
| CEP55 | NUF2 | 0.835 | 0 | 0 | 0.693 | 0.947 |
| CEP55 | SPC25 | 0.795 | 0 | 0 | 0.117 | 0.811 |
| CEP55 | CCNB2 | 0.811 | 0 | 0 | 0.529 | 0.907 |
| CEP55 | MAD2L1 | 0.567 | 0 | 0 | 0.402 | 0.73 |
| CEP55 | TPX2 | 0.804 | 0 | 0 | 0.727 | 0.944 |
| CEP55 | CKS1B | 0.323 | 0 | 0 | 0.161 | 0.408 |
| CEP55 | SKA3 | 0.543 | 0 | 0 | 0.22 | 0.628 |
| CEP55 | CDC25C | 0.471 | 0 | 0 | 0.248 | 0.585 |
| CEP55 | SPAG5 | 0.743 | 0 | 0 | 0.537 | 0.876 |
| CEP55 | NCAPD2 | 0.348 | 0 | 0 | 0.249 | 0.49 |
| CEP55 | CDKN3 | 0.798 | 0 | 0 | 0.154 | 0.822 |
| CEP55 | KNL1 | 0.413 | 0 | 0 | 0.364 | 0.611 |
| CEP55 | CENPA | 0.803 | 0 | 0 | 0.613 | 0.92 |
| CEP55 | CENPF | 0.847 | 0 | 0 | 0.745 | 0.959 |
| CEP55 | NEK2 | 0.816 | 0 | 0 | 0.541 | 0.912 |
| CEP55 | MTFR2 | 0.419 | 0 | 0 | 0.099 | 0.454 |
| CEP55 | BORA | 0.341 | 0 | 0 | 0.286 | 0.509 |
| CEP55 | CENPK | 0.385 | 0 | 0 | 0.292 | 0.547 |
| CEP55 | BRCA2 | 0.253 | 0 | 0 | 0.506 | 0.615 |
| CEP55 | RAD54L | 0.4 | 0 | 0 | 0.391 | 0.619 |
| CEP55 | RAD51 | 0.518 | 0 | 0 | 0.296 | 0.646 |
| CEP55 | CENPI | 0.451 | 0 | 0 | 0.384 | 0.647 |
| CEP55 | KPNA2 | 0.348 | 0 | 0 | 0.494 | 0.656 |
| CEP55 | CKS2 | 0.432 | 0 | 0 | 0.48 | 0.692 |
| CEP55 | UBE2T | 0.465 | 0 | 0 | 0.585 | 0.768 |
| CEP55 | KIF18B | 0.463 | 0 | 0 | 0.586 | 0.768 |
| CEP55 | TK1 | 0.498 | 0 | 0 | 0.622 | 0.802 |
| CEP55 | ZWINT | 0.781 | 0 | 0 | 0.418 | 0.867 |
| CEP55 | DEPDC1 | 0.799 | 0 | 0 | 0.417 | 0.877 |
| CEP55 | PTTG1 | 0.653 | 0 | 0 | 0.702 | 0.892 |
| CEP55 | ECT2 | 0.654 | 0.292 | 0 | 0.673 | 0.913 |
| CEP55 | CCNA2 | 0.822 | 0 | 0 | 0.586 | 0.923 |
| CEP55 | HMMR | 0.873 | 0 | 0 | 0.443 | 0.926 |
| CEP55 | KIF4A | 0.814 | 0 | 0 | 0.748 | 0.951 |
| CEP55 | CDK1 | 0.829 | 0 | 0 | 0.759 | 0.957 |
| CKS1B | CDC6 | 0.279 | 0.153 | 0 | 0.45 | 0.635 |
| CKS1B | CENPM | 0.303 | 0 | 0 | 0.178 | 0.403 |
| CKS1B | AURKA | 0.452 | 0 | 0 | 0.378 | 0.645 |
| CKS1B | GMNN | 0.436 | 0.541 | 0 | 0.168 | 0.766 |
| CKS1B | PIMREG | 0.274 | 0 | 0 | 0.276 | 0.452 |
| CKS1B | NCAPG | 0.305 | 0 | 0 | 0.22 | 0.434 |
| CKS1B | CCNA1 | 0.199 | 0.226 | 0.5 | 0.85 | 0.947 |
| CKS1B | KIF11 | 0.449 | 0 | 0 | 0.243 | 0.565 |
| CKS1B | NUF2 | 0.512 | 0 | 0 | 0.262 | 0.625 |
| CKS1B | CCNB2 | 0.569 | 0.807 | 0 | 0.478 | 0.952 |
| CKS1B | MAD2L1 | 0.647 | 0 | 0 | 0.245 | 0.722 |
| CKS1B | TPX2 | 0.397 | 0 | 0 | 0.31 | 0.566 |
| CKS1B | CEP55 | 0.323 | 0 | 0 | 0.161 | 0.408 |
| CKS1B | RAD51 | 0.298 | 0.067 | 0 | 0.18 | 0.416 |
| CKS1B | ZWINT | 0.342 | 0 | 0 | 0.18 | 0.437 |
| CKS1B | TK1 | 0.383 | 0 | 0 | 0.185 | 0.476 |
| CKS1B | HMMR | 0.363 | 0 | 0 | 0.249 | 0.501 |
| CKS1B | NCAPD2 | 0.229 | 0 | 0 | 0.39 | 0.51 |
| CKS1B | KPNA2 | 0.376 | 0.096 | 0 | 0.199 | 0.51 |
| CKS1B | CDC25C | 0.294 | 0.133 | 0 | 0.278 | 0.519 |
| CKS1B | CENPA | 0.385 | 0 | 0 | 0.284 | 0.541 |
| CKS1B | CENPF | 0.351 | 0 | 0 | 0.394 | 0.591 |
| CKS1B | NEK2 | 0.323 | 0 | 0 | 0.435 | 0.602 |
| CKS1B | CDKN3 | 0.538 | 0.046 | 0 | 0.246 | 0.638 |
| CKS1B | UBE2T | 0.688 | 0.045 | 0 | 0.127 | 0.717 |
| CKS1B | PTTG1 | 0.792 | 0 | 0 | 0.327 | 0.854 |
| CKS1B | CKS2 | 0.556 | 0.783 | 0.9 | 0.395 | 0.993 |
| CKS1B | CDK1 | 0.536 | 0.994 | 0 | 0.978 | 0.999 |
| CKS1B | CCNA2 | 0.489 | 0.976 | 0.5 | 0.905 | 0.999 |
| CKS2 | CDC6 | 0.296 | 0.153 | 0 | 0.451 | 0.644 |
| CKS2 | CENPM | 0.302 | 0 | 0 | 0.245 | 0.45 |
| CKS2 | AURKA | 0.476 | 0 | 0 | 0.642 | 0.804 |
| CKS2 | GMNN | 0.36 | 0.549 | 0 | 0.323 | 0.787 |
| CKS2 | PIMREG | 0.252 | 0 | 0 | 0.291 | 0.447 |
| CKS2 | NCAPG | 0.497 | 0 | 0 | 0.351 | 0.66 |
| CKS2 | CCNA1 | 0.211 | 0.226 | 0 | 0.767 | 0.845 |
| CKS2 | KIF11 | 0.516 | 0 | 0 | 0.355 | 0.675 |
| CKS2 | CENPE | 0.405 | 0 | 0 | 0.324 | 0.581 |
| CKS2 | NUF2 | 0.554 | 0 | 0 | 0.358 | 0.701 |
| CKS2 | SPC25 | 0.404 | 0 | 0 | 0 | 0.404 |
| CKS2 | CCNB2 | 0.56 | 0.797 | 0 | 0.759 | 0.976 |
| CKS2 | MAD2L1 | 0.655 | 0 | 0 | 0.499 | 0.82 |
| CKS2 | TPX2 | 0.455 | 0 | 0 | 0.29 | 0.596 |
| CKS2 | CKS1B | 0.556 | 0.783 | 0.9 | 0.395 | 0.993 |
| CKS2 | SKA3 | 0.34 | 0 | 0 | 0.199 | 0.449 |
| CKS2 | CDC25C | 0.312 | 0.133 | 0 | 0.366 | 0.588 |
| CKS2 | SPAG5 | 0.379 | 0 | 0 | 0.261 | 0.522 |
| CKS2 | NCAPD2 | 0.208 | 0 | 0 | 0.345 | 0.459 |
| CKS2 | CDKN3 | 0.665 | 0.046 | 0 | 0.21 | 0.725 |
| CKS2 | CENPA | 0.419 | 0 | 0 | 0.348 | 0.605 |
| CKS2 | CENPF | 0.367 | 0 | 0 | 0.504 | 0.673 |
| CKS2 | NEK2 | 0.321 | 0 | 0 | 0.392 | 0.569 |
| CKS2 | CEP55 | 0.432 | 0 | 0 | 0.48 | 0.692 |
| CKS2 | ZWINT | 0.396 | 0 | 0 | 0.394 | 0.618 |
| CKS2 | KIF4A | 0.374 | 0 | 0 | 0.224 | 0.494 |
| CKS2 | RAD51 | 0.33 | 0.067 | 0 | 0.179 | 0.442 |
| CKS2 | UBE2T | 0.537 | 0.045 | 0 | 0.188 | 0.61 |
| CKS2 | ECT2 | 0.436 | 0 | 0 | 0.353 | 0.619 |
| CKS2 | TK1 | 0.354 | 0 | 0 | 0.486 | 0.653 |
| CKS2 | HMMR | 0.525 | 0 | 0 | 0.425 | 0.716 |
| CKS2 | KPNA2 | 0.515 | 0.096 | 0 | 0.558 | 0.789 |
| CKS2 | PTTG1 | 0.801 | 0 | 0 | 0.459 | 0.888 |
| CKS2 | CCNA2 | 0.518 | 0.85 | 0 | 0.872 | 0.99 |
| CKS2 | CDK1 | 0.689 | 0.992 | 0 | 0.967 | 0.999 |
| DEPDC1 | CDC6 | 0.452 | 0 | 0 | 0.189 | 0.537 |
| DEPDC1 | AURKA | 0.701 | 0 | 0 | 0.3 | 0.782 |
| DEPDC1 | KNSTRN | 0.299 | 0 | 0 | 0.193 | 0.411 |
| DEPDC1 | PIMREG | 0.41 | 0 | 0 | 0.37 | 0.612 |
| DEPDC1 | NCAPG | 0.784 | 0 | 0 | 0.22 | 0.824 |
| DEPDC1 | TROAP | 0.433 | 0 | 0 | 0.145 | 0.494 |
| DEPDC1 | KIF11 | 0.792 | 0 | 0 | 0.258 | 0.839 |
| DEPDC1 | SGO1 | 0.427 | 0 | 0 | 0.16 | 0.498 |
| DEPDC1 | DEPDC1B | 0.782 | 0 | 0 | 0.086 | 0.798 |
| DEPDC1 | CENPE | 0.793 | 0 | 0 | 0.201 | 0.828 |
| DEPDC1 | NUF2 | 0.802 | 0 | 0 | 0.506 | 0.898 |
| DEPDC1 | SPC25 | 0.756 | 0 | 0 | 0 | 0.756 |
| DEPDC1 | CCNB2 | 0.615 | 0 | 0 | 0.314 | 0.724 |
| DEPDC1 | MAD2L1 | 0.494 | 0 | 0 | 0.21 | 0.583 |
| DEPDC1 | TPX2 | 0.782 | 0 | 0 | 0.573 | 0.903 |
| DEPDC1 | SKA3 | 0.512 | 0 | 0 | 0.439 | 0.714 |
| DEPDC1 | CDC25C | 0.77 | 0 | 0 | 0.201 | 0.808 |
| DEPDC1 | SPAG5 | 0.507 | 0.049 | 0 | 0.223 | 0.604 |
| DEPDC1 | CDKN3 | 0.725 | 0 | 0 | 0.056 | 0.73 |
| DEPDC1 | KNL1 | 0.515 | 0 | 0 | 0.154 | 0.572 |
| DEPDC1 | CENPA | 0.785 | 0 | 0 | 0.37 | 0.858 |
| DEPDC1 | CENPF | 0.754 | 0 | 0 | 0.392 | 0.844 |
| DEPDC1 | NEK2 | 0.789 | 0 | 0 | 0.303 | 0.847 |
| DEPDC1 | CEP55 | 0.799 | 0 | 0 | 0.417 | 0.877 |
| DEPDC1 | RAD54L | 0.343 | 0.059 | 0 | 0.152 | 0.43 |
| DEPDC1 | CENPI | 0.393 | 0 | 0 | 0.16 | 0.469 |
| DEPDC1 | ZWINT | 0.425 | 0 | 0 | 0.202 | 0.521 |
| DEPDC1 | KIF4A | 0.702 | 0 | 0 | 0.348 | 0.797 |
| DEPDC1 | ECT2 | 0.533 | 0 | 0 | 0.279 | 0.649 |
| DEPDC1 | HMMR | 0.781 | 0.049 | 0 | 0.592 | 0.907 |
| DEPDC1 | PTTG1 | 0.412 | 0 | 0 | 0.29 | 0.565 |
| DEPDC1 | CDK1 | 0.79 | 0.051 | 0 | 0.305 | 0.849 |
| DEPDC1 | CENPK | 0.307 | 0 | 0 | 0.254 | 0.461 |
| DEPDC1 | MTFR2 | 0.337 | 0 | 0 | 0.145 | 0.408 |
| DEPDC1 | RAD51 | 0.348 | 0 | 0 | 0.141 | 0.417 |
| DEPDC1 | BORA | 0.29 | 0 | 0 | 0.226 | 0.427 |
| DEPDC1 | TK1 | 0.357 | 0 | 0 | 0.182 | 0.452 |
| DEPDC1 | UBE2T | 0.373 | 0 | 0 | 0.182 | 0.466 |
| DEPDC1 | KIF18B | 0.348 | 0 | 0 | 0.399 | 0.592 |
| DEPDC1 | CCNA2 | 0.797 | 0 | 0 | 0.502 | 0.894 |
| DEPDC1B | CDC6 | 0.369 | 0 | 0 | 0.159 | 0.447 |
| DEPDC1B | CENPM | 0.295 | 0 | 0 | 0.245 | 0.446 |
| DEPDC1B | AURKA | 0.498 | 0 | 0 | 0.058 | 0.507 |
| DEPDC1B | NCAPG | 0.657 | 0 | 0 | 0.27 | 0.739 |
| DEPDC1B | TROAP | 0.369 | 0 | 0 | 0.149 | 0.44 |
| DEPDC1B | KIF11 | 0.812 | 0 | 0 | 0.195 | 0.842 |
| DEPDC1B | ASF1B | 0.303 | 0 | 0 | 0.185 | 0.409 |
| DEPDC1B | SGO1 | 0.45 | 0 | 0 | 0.097 | 0.482 |
| DEPDC1B | KIF18B | 0.411 | 0 | 0 | 0.044 | 0.413 |
| DEPDC1B | RAD51 | 0.349 | 0 | 0 | 0.144 | 0.419 |
| DEPDC1B | CENPK | 0.378 | 0 | 0 | 0.122 | 0.431 |
| DEPDC1B | KNL1 | 0.43 | 0 | 0 | 0.109 | 0.471 |
| DEPDC1B | CDC25C | 0.45 | 0 | 0 | 0.097 | 0.482 |
| DEPDC1B | CDKN3 | 0.479 | 0 | 0 | 0.053 | 0.485 |
| DEPDC1B | ZWINT | 0.45 | 0 | 0 | 0.175 | 0.527 |
| DEPDC1B | CENPI | 0.299 | 0 | 0 | 0.354 | 0.528 |
| DEPDC1B | CENPF | 0.519 | 0 | 0 | 0.069 | 0.533 |
| DEPDC1B | BORA | 0.301 | 0 | 0 | 0.362 | 0.535 |
| DEPDC1B | SPAG5 | 0.51 | 0.049 | 0 | 0.091 | 0.539 |
| DEPDC1B | ECT2 | 0.366 | 0 | 0 | 0.328 | 0.556 |
| DEPDC1B | NEMP1 | 0.111 | 0 | 0 | 0.532 | 0.566 |
| DEPDC1B | SPC25 | 0.573 | 0 | 0 | 0 | 0.573 |
| DEPDC1B | KIF4A | 0.537 | 0 | 0 | 0.164 | 0.597 |
| DEPDC1B | CENPA | 0.563 | 0 | 0 | 0.116 | 0.597 |
| DEPDC1B | SKA3 | 0.567 | 0 | 0 | 0.117 | 0.601 |
| DEPDC1B | CCNB2 | 0.55 | 0 | 0 | 0.168 | 0.61 |
| DEPDC1B | MAD2L1 | 0.527 | 0 | 0 | 0.301 | 0.656 |
| DEPDC1B | TPX2 | 0.649 | 0 | 0 | 0.067 | 0.659 |
| DEPDC1B | CENPE | 0.575 | 0 | 0 | 0.261 | 0.672 |
| DEPDC1B | CCNA2 | 0.67 | 0 | 0 | 0.085 | 0.685 |
| DEPDC1B | UBE2T | 0.466 | 0.292 | 0 | 0.286 | 0.706 |
| DEPDC1B | NEK2 | 0.682 | 0 | 0 | 0.165 | 0.723 |
| DEPDC1B | CDK1 | 0.705 | 0.051 | 0 | 0.217 | 0.762 |
| DEPDC1B | DEPDC1 | 0.782 | 0 | 0 | 0.086 | 0.798 |
| DEPDC1B | NUF2 | 0.799 | 0 | 0 | 0.095 | 0.81 |
| DEPDC1B | HMMR | 0.781 | 0.049 | 0 | 0.171 | 0.812 |
| DEPDC1B | CEP55 | 0.789 | 0 | 0 | 0.249 | 0.834 |
| ECT2 | CDC6 | 0.349 | 0 | 0 | 0.287 | 0.516 |
| ECT2 | AURKA | 0.495 | 0.071 | 0 | 0.67 | 0.831 |
| ECT2 | GMNN | 0.241 | 0 | 0 | 0.503 | 0.607 |
| ECT2 | KNSTRN | 0.38 | 0 | 0 | 0.12 | 0.431 |
| ECT2 | PIMREG | 0.257 | 0 | 0 | 0.324 | 0.477 |
| ECT2 | NCAPG | 0.813 | 0 | 0 | 0.307 | 0.865 |
| ECT2 | CCNA1 | 0.294 | 0 | 0 | 0.191 | 0.405 |
| ECT2 | KIF11 | 0.823 | 0 | 0 | 0.41 | 0.891 |
| ECT2 | SGO1 | 0.469 | 0 | 0 | 0.166 | 0.538 |
| ECT2 | DEPDC1B | 0.366 | 0 | 0 | 0.328 | 0.556 |
| ECT2 | CENPE | 0.62 | 0 | 0 | 0.605 | 0.844 |
| ECT2 | NUF2 | 0.583 | 0 | 0 | 0.581 | 0.818 |
| ECT2 | SPC25 | 0.5 | 0 | 0 | 0 | 0.5 |
| ECT2 | CCNB2 | 0.582 | 0.071 | 0 | 0.583 | 0.824 |
| ECT2 | MAD2L1 | 0.529 | 0 | 0 | 0.551 | 0.779 |
| ECT2 | TPX2 | 0.518 | 0 | 0 | 0.503 | 0.75 |
| ECT2 | SKA3 | 0.332 | 0.198 | 0 | 0.145 | 0.502 |
| ECT2 | CDC25C | 0.329 | 0.106 | 0 | 0.276 | 0.528 |
| ECT2 | SPAG5 | 0.444 | 0.045 | 0 | 0.085 | 0.472 |
| ECT2 | NCAPD2 | 0.226 | 0.292 | 0 | 0.371 | 0.625 |
| ECT2 | CDKN3 | 0.395 | 0.071 | 0 | 0.118 | 0.462 |
| ECT2 | KNL1 | 0.269 | 0 | 0 | 0.468 | 0.595 |
| ECT2 | CENPA | 0.44 | 0 | 0 | 0.376 | 0.635 |
| ECT2 | CENPF | 0.614 | 0 | 0 | 0.429 | 0.77 |
| ECT2 | NEK2 | 0.702 | 0 | 0 | 0.42 | 0.82 |
| ECT2 | CEP55 | 0.654 | 0.292 | 0 | 0.673 | 0.913 |
| ECT2 | CENPI | 0.343 | 0 | 0 | 0.21 | 0.459 |
| ECT2 | ZWINT | 0.315 | 0 | 0 | 0.545 | 0.675 |
| ECT2 | KIF4A | 0.632 | 0 | 0 | 0.53 | 0.82 |
| ECT2 | CKS2 | 0.436 | 0 | 0 | 0.353 | 0.619 |
| ECT2 | KIF18B | 0.306 | 0 | 0 | 0.201 | 0.422 |
| ECT2 | AUNIP | 0.174 | 0 | 0 | 0.34 | 0.432 |
| ECT2 | RAD51 | 0.446 | 0 | 0 | 0.153 | 0.511 |
| ECT2 | UBE2T | 0.285 | 0 | 0 | 0.383 | 0.54 |
| ECT2 | TK1 | 0.23 | 0 | 0 | 0.446 | 0.555 |
| ECT2 | CENPK | 0.421 | 0 | 0 | 0.321 | 0.591 |
| ECT2 | PTTG1 | 0.404 | 0 | 0 | 0.394 | 0.623 |
| ECT2 | DEPDC1 | 0.533 | 0 | 0 | 0.279 | 0.649 |
| ECT2 | KPNA2 | 0.504 | 0.303 | 0 | 0.174 | 0.69 |
| ECT2 | BORA | 0.314 | 0 | 0 | 0.632 | 0.737 |
| ECT2 | CCNA2 | 0.639 | 0 | 0 | 0.33 | 0.748 |
| ECT2 | HMMR | 0.501 | 0.045 | 0 | 0.593 | 0.789 |
| ECT2 | CDK1 | 0.774 | 0.071 | 0 | 0.838 | 0.963 |
| EME1 | CDC6 | 0.273 | 0 | 0 | 0.208 | 0.4 |
| EME1 | ORC6 | 0.184 | 0 | 0 | 0.465 | 0.545 |
| EME1 | RAG1 | 0.06 | 0.047 | 0.54 | 0.09 | 0.575 |
| EME1 | RMI2 | 0.191 | 0 | 0.5 | 0.596 | 0.822 |
| EME1 | CDC25C | 0.273 | 0 | 0 | 0.242 | 0.426 |
| EME1 | SPAG5 | 0.284 | 0.105 | 0 | 0.153 | 0.41 |
| EME1 | RAD54L | 0.27 | 0.334 | 0 | 0.517 | 0.745 |
| EME1 | BRCA2 | 0.144 | 0 | 0.5 | 0.549 | 0.79 |
| EME1 | CCNA2 | 0.255 | 0.054 | 0 | 0.22 | 0.403 |
| EME1 | CDK1 | 0.269 | 0.045 | 0 | 0.543 | 0.653 |
| EME1 | UBE2T | 0.233 | 0.047 | 0.5 | 0.309 | 0.713 |
| EME1 | RAD51 | 0.363 | 0.051 | 0.5 | 0.655 | 0.881 |
| GMNN | CDC6 | 0.382 | 0 | 0.5 | 0.717 | 0.905 |
| GMNN | AURKA | 0.314 | 0.292 | 0 | 0.321 | 0.641 |
| GMNN | ORC6 | 0.236 | 0 | 0.5 | 0.745 | 0.894 |
| GMNN | WDR76 | 0.098 | 0 | 0 | 0.365 | 0.402 |
| GMNN | NCAPD2 | 0.189 | 0 | 0 | 0.3 | 0.409 |
| GMNN | TK1 | 0.297 | 0 | 0 | 0.206 | 0.418 |
| GMNN | SPAG5 | 0.353 | 0 | 0 | 0.153 | 0.429 |
| GMNN | RAD51 | 0.322 | 0 | 0 | 0.208 | 0.44 |
| GMNN | CENPK | 0.289 | 0 | 0 | 0.283 | 0.468 |
| GMNN | CENPE | 0.234 | 0.114 | 0 | 0.298 | 0.482 |
| GMNN | CENPF | 0.254 | 0 | 0 | 0.348 | 0.492 |
| GMNN | ZWINT | 0.5 | 0 | 0 | 0 | 0.5 |
| GMNN | NCAPG | 0.488 | 0 | 0 | 0.119 | 0.53 |
| GMNN | PTTG1 | 0.355 | 0 | 0 | 0.305 | 0.532 |
| GMNN | HMMR | 0.347 | 0.234 | 0 | 0.203 | 0.567 |
| GMNN | CCNB2 | 0.366 | 0 | 0 | 0.349 | 0.569 |
| GMNN | CCNA1 | 0.111 | 0 | 0 | 0.547 | 0.58 |
| GMNN | NUF2 | 0.398 | 0 | 0 | 0.353 | 0.594 |
| GMNN | ECT2 | 0.241 | 0 | 0 | 0.503 | 0.607 |
| GMNN | MAD2L1 | 0.523 | 0 | 0 | 0.223 | 0.614 |
| GMNN | KIF11 | 0.418 | 0 | 0 | 0.391 | 0.631 |
| GMNN | UBE2T | 0.626 | 0 | 0 | 0.212 | 0.693 |
| GMNN | CKS1B | 0.436 | 0.541 | 0 | 0.168 | 0.766 |
| GMNN | CKS2 | 0.36 | 0.549 | 0 | 0.323 | 0.787 |
| GMNN | CDK1 | 0.52 | 0.623 | 0 | 0.325 | 0.867 |
| GMNN | CCNA2 | 0.427 | 0.68 | 0 | 0.41 | 0.882 |
| HMMR | CDC6 | 0.43 | 0 | 0 | 0.299 | 0.584 |
| HMMR | CENPM | 0.475 | 0 | 0 | 0.302 | 0.618 |
| HMMR | AURKA | 0.794 | 0.292 | 0 | 0.811 | 0.97 |
| HMMR | GMNN | 0.347 | 0.234 | 0 | 0.203 | 0.567 |
| HMMR | KNSTRN | 0.379 | 0 | 0 | 0.078 | 0.403 |
| HMMR | PIMREG | 0.4 | 0 | 0 | 0.359 | 0.599 |
| HMMR | NCAPG | 0.817 | 0 | 0 | 0.523 | 0.909 |
| HMMR | TROAP | 0.516 | 0 | 0 | 0.118 | 0.555 |
| HMMR | KIF11 | 0.815 | 0.049 | 0 | 0.623 | 0.928 |
| HMMR | ASF1B | 0.343 | 0.089 | 0 | 0.279 | 0.531 |
| HMMR | SGO1 | 0.385 | 0 | 0 | 0.17 | 0.468 |
| HMMR | DEPDC1B | 0.781 | 0.049 | 0 | 0.171 | 0.812 |
| HMMR | CENPE | 0.807 | 0.057 | 0 | 0.625 | 0.926 |
| HMMR | NUF2 | 0.817 | 0.053 | 0 | 0.395 | 0.886 |
| HMMR | SPC25 | 0.786 | 0.053 | 0 | 0.112 | 0.804 |
| HMMR | CCNB2 | 0.796 | 0.091 | 0 | 0.446 | 0.888 |
| HMMR | MAD2L1 | 0.682 | 0.079 | 0 | 0.375 | 0.801 |
| HMMR | TPX2 | 0.799 | 0.328 | 0 | 0.856 | 0.979 |
| HMMR | CKS1B | 0.363 | 0 | 0 | 0.249 | 0.501 |
| HMMR | SKA3 | 0.652 | 0 | 0 | 0.642 | 0.87 |
| HMMR | CDC25C | 0.654 | 0 | 0 | 0.223 | 0.72 |
| HMMR | SPAG5 | 0.647 | 0 | 0 | 0.186 | 0.701 |
| HMMR | NCAPD2 | 0.332 | 0 | 0 | 0.264 | 0.487 |
| HMMR | CDKN3 | 0.801 | 0.089 | 0 | 0.154 | 0.833 |
| HMMR | KNL1 | 0.471 | 0 | 0 | 0.109 | 0.508 |
| HMMR | CENPA | 0.799 | 0.078 | 0 | 0.359 | 0.87 |
| HMMR | CENPF | 0.787 | 0.045 | 0 | 0.669 | 0.927 |
| HMMR | NEK2 | 0.779 | 0.043 | 0 | 0.436 | 0.87 |
| HMMR | CEP55 | 0.873 | 0 | 0 | 0.443 | 0.926 |
| HMMR | RAD54L | 0.378 | 0.099 | 0 | 0.232 | 0.532 |
| HMMR | CENPI | 0.314 | 0.128 | 0 | 0.147 | 0.446 |
| HMMR | ZWINT | 0.772 | 0 | 0 | 0.543 | 0.891 |
| HMMR | KIF4A | 0.797 | 0.091 | 0 | 0.388 | 0.877 |
| HMMR | CKS2 | 0.525 | 0 | 0 | 0.425 | 0.716 |
| HMMR | BRCA2 | 0.256 | 0 | 0 | 0.909 | 0.929 |
| HMMR | ECT2 | 0.501 | 0.045 | 0 | 0.593 | 0.789 |
| HMMR | KIF18B | 0.353 | 0.057 | 0 | 0.145 | 0.433 |
| HMMR | KPNA2 | 0.329 | 0.105 | 0 | 0.168 | 0.458 |
| HMMR | CENPK | 0.383 | 0 | 0 | 0.184 | 0.475 |
| HMMR | RAD51 | 0.444 | 0.046 | 0 | 0.216 | 0.548 |
| HMMR | TK1 | 0.454 | 0 | 0 | 0.222 | 0.557 |
| HMMR | UBE2T | 0.495 | 0.08 | 0 | 0.168 | 0.579 |
| HMMR | PTTG1 | 0.781 | 0 | 0 | 0.391 | 0.861 |
| HMMR | DEPDC1 | 0.781 | 0.049 | 0 | 0.592 | 0.907 |
| HMMR | CCNA2 | 0.802 | 0 | 0 | 0.619 | 0.921 |
| HMMR | CDK1 | 0.829 | 0.094 | 0 | 0.59 | 0.931 |
| KIF11 | CDC6 | 0.78 | 0 | 0 | 0.434 | 0.87 |
| KIF11 | CENPM | 0.512 | 0 | 0 | 0.606 | 0.799 |
| KIF11 | AURKA | 0.812 | 0.272 | 0 | 0.816 | 0.972 |
| KIF11 | ORC6 | 0.256 | 0 | 0 | 0.329 | 0.48 |
| KIF11 | GMNN | 0.418 | 0 | 0 | 0.391 | 0.631 |
| KIF11 | KNSTRN | 0.449 | 0 | 0 | 0.348 | 0.625 |
| KIF11 | PIMREG | 0.386 | 0 | 0 | 0.328 | 0.57 |
| KIF11 | NCAPG | 0.912 | 0.086 | 0 | 0.722 | 0.975 |
| KIF11 | CCNA1 | 0.595 | 0 | 0 | 0.295 | 0.703 |
| KIF11 | TROAP | 0.554 | 0 | 0 | 0.136 | 0.598 |
| KIF11 | BRCA2 | 0.365 | 0 | 0 | 0.189 | 0.463 |
| KIF11 | UBE2T | 0.438 | 0 | 0 | 0.179 | 0.519 |
| KIF11 | BORA | 0.468 | 0 | 0 | 0.175 | 0.543 |
| KIF11 | CKS1B | 0.449 | 0 | 0 | 0.243 | 0.565 |
| KIF11 | KPNA2 | 0.509 | 0 | 0 | 0.189 | 0.585 |
| KIF11 | WDR76 | 0.32 | 0.292 | 0 | 0.21 | 0.586 |
| KIF11 | CENPK | 0.503 | 0 | 0 | 0.335 | 0.656 |
| KIF11 | RAD54L | 0.488 | 0.068 | 0 | 0.339 | 0.657 |
| KIF11 | CDC25C | 0.51 | 0 | 0 | 0.335 | 0.66 |
| KIF11 | SKA3 | 0.559 | 0 | 0 | 0.292 | 0.675 |
| KIF11 | CKS2 | 0.516 | 0 | 0 | 0.355 | 0.675 |
| KIF11 | ASF1B | 0.507 | 0 | 0 | 0.368 | 0.675 |
| KIF11 | RAD51 | 0.596 | 0 | 0 | 0.276 | 0.695 |
| KIF11 | KNL1 | 0.506 | 0 | 0 | 0.534 | 0.759 |
| KIF11 | TK1 | 0.483 | 0 | 0 | 0.557 | 0.761 |
| KIF11 | SPC25 | 0.785 | 0 | 0 | 0.088 | 0.796 |
| KIF11 | CENPI | 0.523 | 0.438 | 0 | 0.344 | 0.809 |
| KIF11 | DEPDC1 | 0.792 | 0 | 0 | 0.258 | 0.839 |
| KIF11 | SGO1 | 0.714 | 0 | 0 | 0.467 | 0.841 |
| KIF11 | DEPDC1B | 0.812 | 0 | 0 | 0.195 | 0.842 |
| KIF11 | CDKN3 | 0.794 | 0.096 | 0 | 0.25 | 0.848 |
| KIF11 | PTTG1 | 0.569 | 0 | 0 | 0.687 | 0.859 |
| KIF11 | NCAPD2 | 0.752 | 0 | 0 | 0.461 | 0.86 |
| KIF11 | KIF18B | 0.593 | 0.158 | 0.54 | 0.278 | 0.871 |
| KIF11 | ECT2 | 0.823 | 0 | 0 | 0.41 | 0.891 |
| KIF11 | HMMR | 0.815 | 0.049 | 0 | 0.623 | 0.928 |
| KIF11 | ZWINT | 0.794 | 0 | 0 | 0.665 | 0.928 |
| KIF11 | CENPA | 0.832 | 0.078 | 0 | 0.658 | 0.942 |
| KIF11 | CCNB2 | 0.841 | 0.136 | 0 | 0.629 | 0.944 |
| KIF11 | KIF4A | 0.891 | 0 | 0.4 | 0.324 | 0.952 |
| KIF11 | SPAG5 | 0.883 | 0.071 | 0 | 0.613 | 0.954 |
| KIF11 | NEK2 | 0.845 | 0.054 | 0 | 0.745 | 0.959 |
| KIF11 | TPX2 | 0.843 | 0 | 0 | 0.79 | 0.965 |
| KIF11 | CENPF | 0.903 | 0 | 0 | 0.728 | 0.972 |
| KIF11 | MAD2L1 | 0.773 | 0.519 | 0 | 0.775 | 0.973 |
| KIF11 | NUF2 | 0.925 | 0.084 | 0 | 0.669 | 0.975 |
| KIF11 | CCNA2 | 0.913 | 0 | 0 | 0.762 | 0.978 |
| KIF11 | CDK1 | 0.858 | 0.364 | 0 | 0.869 | 0.987 |
| KIF11 | CENPE | 0.958 | 0.305 | 0.4 | 0.419 | 0.988 |
| KIF11 | CEP55 | 0.994 | 0 | 0 | 0.519 | 0.997 |
| KIF18B | CDC6 | 0.292 | 0 | 0 | 0.228 | 0.43 |
| KIF18B | CENPM | 0.264 | 0 | 0 | 0.255 | 0.428 |
| KIF18B | AURKA | 0.393 | 0.094 | 0 | 0.342 | 0.607 |
| KIF18B | KNSTRN | 0.328 | 0 | 0 | 0.225 | 0.457 |
| KIF18B | NCAPG | 0.378 | 0 | 0 | 0.423 | 0.626 |
| KIF18B | TROAP | 0.86 | 0 | 0 | 0.356 | 0.906 |
| KIF18B | KIF11 | 0.593 | 0.158 | 0.54 | 0.278 | 0.871 |
| KIF18B | PRR11 | 0.393 | 0 | 0 | 0.116 | 0.44 |
| KIF18B | ASF1B | 0.381 | 0 | 0 | 0.249 | 0.515 |
| KIF18B | SGO1 | 0.383 | 0.068 | 0 | 0.348 | 0.592 |
| KIF18B | DEPDC1B | 0.411 | 0 | 0 | 0.044 | 0.413 |
| KIF18B | CENPE | 0.386 | 0 | 0.4 | 0.221 | 0.688 |
| KIF18B | NUF2 | 0.386 | 0.06 | 0 | 0.568 | 0.729 |
| KIF18B | SPC25 | 0.367 | 0 | 0 | 0.091 | 0.4 |
| KIF18B | CCNB2 | 0.309 | 0.051 | 0 | 0.313 | 0.51 |
| KIF18B | MAD2L1 | 0.238 | 0.222 | 0 | 0.225 | 0.501 |
| KIF18B | TPX2 | 0.42 | 0 | 0 | 0.476 | 0.683 |
| KIF18B | SKA3 | 0.361 | 0 | 0 | 0.271 | 0.514 |
| KIF18B | CDC25C | 0.238 | 0.085 | 0 | 0.398 | 0.544 |
| KIF18B | SPAG5 | 0.563 | 0.057 | 0 | 0.37 | 0.718 |
| KIF18B | NCAPD2 | 0.269 | 0 | 0 | 0.26 | 0.436 |
| KIF18B | CENPA | 0.331 | 0.135 | 0 | 0.4 | 0.622 |
| KIF18B | CENPF | 0.524 | 0 | 0 | 0.392 | 0.698 |
| KIF18B | NEK2 | 0.447 | 0 | 0 | 0.201 | 0.539 |
| KIF18B | CEP55 | 0.463 | 0 | 0 | 0.586 | 0.768 |
| KIF18B | RAD54L | 0.259 | 0.066 | 0 | 0.409 | 0.555 |
| KIF18B | CENPI | 0.316 | 0.114 | 0 | 0.399 | 0.604 |
| KIF18B | ZWINT | 0.358 | 0.065 | 0 | 0.1 | 0.413 |
| KIF18B | KIF4A | 0.693 | 0 | 0.4 | 0.506 | 0.901 |
| KIF18B | ARHGEF39 | 0.4 | 0 | 0 | 0 | 0.4 |
| KIF18B | ECT2 | 0.306 | 0 | 0 | 0.201 | 0.422 |
| KIF18B | HMMR | 0.353 | 0.057 | 0 | 0.145 | 0.433 |
| KIF18B | PTTG1 | 0.275 | 0 | 0 | 0.23 | 0.418 |
| KIF18B | CDK1 | 0.321 | 0.096 | 0 | 0.306 | 0.537 |
| KIF18B | TONSL | 0.487 | 0.057 | 0 | 0 | 0.495 |
| KIF18B | DEPDC1 | 0.348 | 0 | 0 | 0.399 | 0.592 |
| KIF18B | KPNA2 | 0.179 | 0.147 | 0 | 0.258 | 0.436 |
| KIF18B | TK1 | 0.44 | 0 | 0 | 0.069 | 0.456 |
| KIF18B | CCNA2 | 0.461 | 0.091 | 0 | 0.273 | 0.613 |
| KIF4A | CDC6 | 0.427 | 0 | 0 | 0.268 | 0.563 |
| KIF4A | CENPM | 0.547 | 0 | 0 | 0.451 | 0.741 |
| KIF4A | AURKA | 0.692 | 0 | 0 | 0.642 | 0.885 |
| KIF4A | KNSTRN | 0.382 | 0 | 0 | 0.153 | 0.454 |
| KIF4A | PIMREG | 0.539 | 0 | 0 | 0.348 | 0.687 |
| KIF4A | NCAPG | 0.843 | 0 | 0 | 0.599 | 0.934 |
| KIF4A | TROAP | 0.668 | 0 | 0 | 0.527 | 0.836 |
| KIF4A | KIF11 | 0.891 | 0 | 0.4 | 0.324 | 0.952 |
| KIF4A | ASF1B | 0.496 | 0 | 0 | 0.27 | 0.616 |
| KIF4A | SGO1 | 0.519 | 0 | 0 | 0.397 | 0.698 |
| KIF4A | DEPDC1B | 0.537 | 0 | 0 | 0.164 | 0.597 |
| KIF4A | CENPE | 0.839 | 0 | 0.4 | 0.589 | 0.957 |
| KIF4A | NUF2 | 0.695 | 0 | 0 | 0.721 | 0.911 |
| KIF4A | SPC25 | 0.718 | 0 | 0 | 0.185 | 0.761 |
| KIF4A | CCNB2 | 0.724 | 0 | 0 | 0.529 | 0.864 |
| KIF4A | MAD2L1 | 0.526 | 0 | 0 | 0.447 | 0.726 |
| KIF4A | TPX2 | 0.861 | 0 | 0 | 0.764 | 0.965 |
| KIF4A | SKA3 | 0.676 | 0 | 0 | 0.445 | 0.813 |
| KIF4A | CDC25C | 0.648 | 0 | 0 | 0.45 | 0.798 |
| KIF4A | SPAG5 | 0.809 | 0.091 | 0 | 0.406 | 0.887 |
| KIF4A | NCAPD2 | 0.541 | 0.094 | 0 | 0.393 | 0.725 |
| KIF4A | CDKN3 | 0.787 | 0 | 0 | 0.111 | 0.803 |
| KIF4A | KNL1 | 0.375 | 0 | 0 | 0.369 | 0.588 |
| KIF4A | CENPA | 0.799 | 0.125 | 0 | 0.51 | 0.906 |
| KIF4A | CENPF | 0.73 | 0 | 0 | 0.571 | 0.879 |
| KIF4A | NEK2 | 0.649 | 0 | 0 | 0.611 | 0.858 |
| KIF4A | CEP55 | 0.814 | 0 | 0 | 0.748 | 0.951 |
| KIF4A | RAD54L | 0.416 | 0.065 | 0 | 0.325 | 0.599 |
| KIF4A | CENPI | 0.542 | 0 | 0 | 0.278 | 0.655 |
| KIF4A | ZWINT | 0.745 | 0 | 0 | 0.43 | 0.848 |
| KIF4A | KPNA2 | 0.37 | 0 | 0 | 0.18 | 0.461 |
| KIF4A | CENPK | 0.336 | 0 | 0 | 0.249 | 0.48 |
| KIF4A | BORA | 0.412 | 0 | 0 | 0.155 | 0.482 |
| KIF4A | CKS2 | 0.374 | 0 | 0 | 0.224 | 0.494 |
| KIF4A | UBE2T | 0.387 | 0.07 | 0 | 0.51 | 0.696 |
| KIF4A | TK1 | 0.521 | 0 | 0 | 0.436 | 0.718 |
| KIF4A | BRCA2 | 0.238 | 0.292 | 0 | 0.532 | 0.725 |
| KIF4A | PTTG1 | 0.535 | 0 | 0 | 0.559 | 0.786 |
| KIF4A | DEPDC1 | 0.702 | 0 | 0 | 0.348 | 0.797 |
| KIF4A | RAD51 | 0.456 | 0 | 0 | 0.654 | 0.803 |
| KIF4A | ECT2 | 0.632 | 0 | 0 | 0.53 | 0.82 |
| KIF4A | HMMR | 0.797 | 0.091 | 0 | 0.388 | 0.877 |
| KIF4A | CCNA2 | 0.828 | 0 | 0 | 0.417 | 0.895 |
| KIF4A | KIF18B | 0.693 | 0 | 0.4 | 0.506 | 0.901 |
| KIF4A | CDK1 | 0.82 | 0 | 0 | 0.527 | 0.911 |
| KNL1 | CENPM | 0.259 | 0 | 0.5 | 0.528 | 0.809 |
| KNL1 | AURKA | 0.306 | 0.063 | 0 | 0.568 | 0.695 |
| KNL1 | KNSTRN | 0.221 | 0 | 0 | 0.394 | 0.507 |
| KNL1 | NCAPG | 0.502 | 0 | 0 | 0.33 | 0.652 |
| KNL1 | KIF11 | 0.506 | 0 | 0 | 0.534 | 0.759 |
| KNL1 | SGO1 | 0.526 | 0 | 0 | 0.583 | 0.793 |
| KNL1 | DEPDC1B | 0.43 | 0 | 0 | 0.109 | 0.471 |
| KNL1 | CENPE | 0.466 | 0 | 0 | 0.82 | 0.9 |
| KNL1 | NUF2 | 0.513 | 0.827 | 0.54 | 0.823 | 0.992 |
| KNL1 | SPC25 | 0.394 | 0.845 | 0.54 | 0.142 | 0.958 |
| KNL1 | CCNB2 | 0.398 | 0.117 | 0.4 | 0.527 | 0.829 |
| KNL1 | MAD2L1 | 0.38 | 0.068 | 0.5 | 0.437 | 0.815 |
| KNL1 | TPX2 | 0.406 | 0 | 0 | 0.275 | 0.551 |
| KNL1 | SKA3 | 0.39 | 0 | 0 | 0.713 | 0.818 |
| KNL1 | CDC25C | 0.346 | 0 | 0 | 0.138 | 0.413 |
| KNL1 | SPAG5 | 0.367 | 0 | 0 | 0.352 | 0.572 |
| KNL1 | NCAPD2 | 0.224 | 0 | 0 | 0.381 | 0.499 |
| KNL1 | TK1 | 0.298 | 0 | 0 | 0.18 | 0.4 |
| KNL1 | PSMC3IP | 0.144 | 0.11 | 0 | 0.306 | 0.425 |
| KNL1 | HMMR | 0.471 | 0 | 0 | 0.109 | 0.508 |
| KNL1 | CCNA2 | 0.406 | 0 | 0 | 0.227 | 0.522 |
| KNL1 | NEK2 | 0.373 | 0 | 0 | 0.321 | 0.556 |
| KNL1 | RAD54L | 0.29 | 0.297 | 0 | 0.191 | 0.561 |
| KNL1 | DEPDC1 | 0.515 | 0 | 0 | 0.154 | 0.572 |
| KNL1 | PTTG1 | 0.244 | 0 | 0 | 0.478 | 0.588 |
| KNL1 | KIF4A | 0.375 | 0 | 0 | 0.369 | 0.588 |
| KNL1 | RAD51 | 0.37 | 0.31 | 0 | 0.091 | 0.589 |
| KNL1 | ECT2 | 0.269 | 0 | 0 | 0.468 | 0.595 |
| KNL1 | CEP55 | 0.413 | 0 | 0 | 0.364 | 0.611 |
| KNL1 | BRCA2 | 0.337 | 0.506 | 0 | 0.272 | 0.741 |
| KNL1 | CENPF | 0.498 | 0 | 0 | 0.588 | 0.784 |
| KNL1 | CDK1 | 0.479 | 0.068 | 0.4 | 0.512 | 0.838 |
| KNL1 | CENPK | 0.326 | 0 | 0.5 | 0.602 | 0.854 |
| KNL1 | CENPI | 0.433 | 0 | 0.5 | 0.671 | 0.898 |
| KNL1 | CENPA | 0.352 | 0.084 | 0.5 | 0.712 | 0.903 |
| KNL1 | ZWINT | 0.259 | 0.913 | 0.54 | 0.986 | 0.999 |
| KNSTRN | AURKA | 0.429 | 0 | 0 | 0.229 | 0.541 |
| KNSTRN | HMMR | 0.379 | 0 | 0 | 0.078 | 0.403 |
| KNSTRN | RAD51 | 0.374 | 0 | 0 | 0.091 | 0.406 |
| KNSTRN | SPC25 | 0.408 | 0 | 0 | 0 | 0.408 |
| KNSTRN | ZWINT | 0.27 | 0 | 0 | 0.224 | 0.409 |
| KNSTRN | DEPDC1 | 0.299 | 0 | 0 | 0.193 | 0.411 |
| KNSTRN | TPX2 | 0.341 | 0 | 0 | 0.145 | 0.413 |
| KNSTRN | PTTG1 | 0.335 | 0 | 0 | 0.179 | 0.43 |
| KNSTRN | MAD2L1 | 0.349 | 0 | 0 | 0.159 | 0.43 |
| KNSTRN | ECT2 | 0.38 | 0 | 0 | 0.12 | 0.431 |
| KNSTRN | CCNB2 | 0.382 | 0 | 0 | 0.126 | 0.437 |
| KNSTRN | PIMREG | 0.384 | 0 | 0 | 0.125 | 0.438 |
| KNSTRN | CDKN3 | 0.452 | 0 | 0 | 0 | 0.452 |
| KNSTRN | KIF4A | 0.382 | 0 | 0 | 0.153 | 0.454 |
| KNSTRN | KIF18B | 0.328 | 0 | 0 | 0.225 | 0.457 |
| KNSTRN | CENPK | 0.231 | 0 | 0 | 0.339 | 0.47 |
| KNSTRN | CENPI | 0.279 | 0 | 0 | 0.307 | 0.479 |
| KNSTRN | NCAPG | 0.415 | 0 | 0 | 0.17 | 0.494 |
| KNSTRN | KNL1 | 0.221 | 0 | 0 | 0.394 | 0.507 |
| KNSTRN | SGO1 | 0.371 | 0 | 0 | 0.276 | 0.525 |
| KNSTRN | CENPA | 0.444 | 0 | 0 | 0.287 | 0.587 |
| KNSTRN | CCNA2 | 0.587 | 0 | 0 | 0.112 | 0.618 |
| KNSTRN | CENPF | 0.411 | 0 | 0 | 0.385 | 0.623 |
| KNSTRN | KIF11 | 0.449 | 0 | 0 | 0.348 | 0.625 |
| KNSTRN | CDK1 | 0.543 | 0 | 0 | 0.247 | 0.641 |
| KNSTRN | SKA3 | 0.403 | 0 | 0 | 0.45 | 0.658 |
| KNSTRN | CEP55 | 0.465 | 0 | 0 | 0.477 | 0.708 |
| KNSTRN | NUF2 | 0.681 | 0 | 0 | 0.541 | 0.847 |
| KNSTRN | CENPE | 0.453 | 0 | 0 | 0.807 | 0.89 |
| KNSTRN | SPAG5 | 0.594 | 0.435 | 0 | 0.934 | 0.983 |
| KPNA2 | CDC6 | 0.367 | 0.14 | 0 | 0.209 | 0.532 |
| KPNA2 | AURKA | 0.469 | 0 | 0 | 0.306 | 0.616 |
| KPNA2 | NCAPG | 0.475 | 0 | 0 | 0.294 | 0.614 |
| KPNA2 | KIF11 | 0.509 | 0 | 0 | 0.189 | 0.585 |
| KPNA2 | SGO1 | 0.386 | 0.094 | 0 | 0.07 | 0.438 |
| KPNA2 | NUF2 | 0.484 | 0 | 0 | 0.128 | 0.531 |
| KPNA2 | CCNB2 | 0.67 | 0.128 | 0 | 0.343 | 0.794 |
| KPNA2 | MAD2L1 | 0.508 | 0 | 0 | 0.29 | 0.636 |
| KPNA2 | RAG1 | 0.047 | 0.466 | 0 | 0.619 | 0.789 |
| KPNA2 | TPX2 | 0.611 | 0.297 | 0 | 0.552 | 0.867 |
| KPNA2 | CKS1B | 0.376 | 0.096 | 0 | 0.199 | 0.51 |
| KPNA2 | SPAG5 | 0.458 | 0.105 | 0 | 0.126 | 0.539 |
| KPNA2 | NCAPD2 | 0.294 | 0 | 0 | 0.191 | 0.405 |
| KPNA2 | CENPA | 0.278 | 0.087 | 0 | 0.183 | 0.415 |
| KPNA2 | CENPF | 0.362 | 0 | 0 | 0.222 | 0.483 |
| KPNA2 | NEK2 | 0.439 | 0 | 0 | 0.193 | 0.528 |
| KPNA2 | CEP55 | 0.348 | 0 | 0 | 0.494 | 0.656 |
| KPNA2 | RAD54L | 0.295 | 0.134 | 0 | 0.151 | 0.437 |
| KPNA2 | ZWINT | 0.358 | 0 | 0 | 0.246 | 0.495 |
| KPNA2 | KIF4A | 0.37 | 0 | 0 | 0.18 | 0.461 |
| KPNA2 | CKS2 | 0.515 | 0.096 | 0 | 0.558 | 0.789 |
| KPNA2 | ECT2 | 0.504 | 0.303 | 0 | 0.174 | 0.69 |
| KPNA2 | HMMR | 0.329 | 0.105 | 0 | 0.168 | 0.458 |
| KPNA2 | PTTG1 | 0.549 | 0 | 0 | 0.255 | 0.65 |
| KPNA2 | CDK1 | 0.634 | 0.097 | 0 | 0.577 | 0.848 |
| KPNA2 | UBE2T | 0.385 | 0 | 0 | 0.066 | 0.402 |
| KPNA2 | KIF18B | 0.179 | 0.147 | 0 | 0.258 | 0.436 |
| KPNA2 | RAD51 | 0.442 | 0.046 | 0 | 0.148 | 0.507 |
| KPNA2 | CCNA2 | 0.448 | 0.093 | 0 | 0.241 | 0.587 |
| KPNA2 | TK1 | 0.675 | 0 | 0 | 0.137 | 0.707 |
| MAD2L1 | CDC6 | 0.501 | 0 | 0 | 0.528 | 0.754 |
| MAD2L1 | CENPM | 0.541 | 0 | 0.5 | 0.27 | 0.818 |
| MAD2L1 | AURKA | 0.647 | 0.1 | 0 | 0.796 | 0.929 |
| MAD2L1 | ORC6 | 0.48 | 0 | 0 | 0.34 | 0.642 |
| MAD2L1 | GMNN | 0.523 | 0 | 0 | 0.223 | 0.614 |
| MAD2L1 | KNSTRN | 0.349 | 0 | 0 | 0.159 | 0.43 |
| MAD2L1 | NCAPG | 0.574 | 0.088 | 0 | 0.614 | 0.837 |
| MAD2L1 | CCNA1 | 0.358 | 0 | 0.5 | 0.282 | 0.749 |
| MAD2L1 | KIF11 | 0.773 | 0.519 | 0 | 0.775 | 0.973 |
| MAD2L1 | ASF1B | 0.428 | 0 | 0 | 0.18 | 0.511 |
| MAD2L1 | SGO1 | 0.305 | 0.1 | 0.5 | 0.734 | 0.905 |
| MAD2L1 | DEPDC1B | 0.527 | 0 | 0 | 0.301 | 0.656 |
| MAD2L1 | CENPE | 0.45 | 0.222 | 0.5 | 0.762 | 0.942 |
| MAD2L1 | NUF2 | 0.91 | 0.107 | 0.5 | 0.581 | 0.981 |
| MAD2L1 | SPC25 | 0.737 | 0 | 0.5 | 0.395 | 0.913 |
| MAD2L1 | CCNB2 | 0.615 | 0.127 | 0.4 | 0.835 | 0.962 |
| MAD2L1 | BRCA2 | 0.201 | 0 | 0 | 0.286 | 0.406 |
| MAD2L1 | NEMP1 | 0.2 | 0 | 0 | 0.304 | 0.419 |
| MAD2L1 | PSMC3IP | 0.366 | 0 | 0 | 0.129 | 0.424 |
| MAD2L1 | BORA | 0.415 | 0 | 0 | 0.179 | 0.499 |
| MAD2L1 | KIF18B | 0.238 | 0.222 | 0 | 0.225 | 0.501 |
| MAD2L1 | NCAPD2 | 0.37 | 0 | 0 | 0.267 | 0.519 |
| MAD2L1 | RAD54L | 0.358 | 0.096 | 0 | 0.255 | 0.53 |
| MAD2L1 | SKA3 | 0.445 | 0 | 0 | 0.232 | 0.555 |
| MAD2L1 | DEPDC1 | 0.494 | 0 | 0 | 0.21 | 0.583 |
| MAD2L1 | KPNA2 | 0.508 | 0 | 0 | 0.29 | 0.636 |
| MAD2L1 | CDC25C | 0.396 | 0.075 | 0 | 0.406 | 0.639 |
| MAD2L1 | TK1 | 0.452 | 0 | 0 | 0.5 | 0.714 |
| MAD2L1 | CKS1B | 0.647 | 0 | 0 | 0.245 | 0.722 |
| MAD2L1 | KIF4A | 0.526 | 0 | 0 | 0.447 | 0.726 |
| MAD2L1 | CEP55 | 0.567 | 0 | 0 | 0.402 | 0.73 |
| MAD2L1 | SPAG5 | 0.407 | 0.096 | 0 | 0.562 | 0.745 |
| MAD2L1 | ECT2 | 0.529 | 0 | 0 | 0.551 | 0.779 |
| MAD2L1 | RAD51 | 0.695 | 0.088 | 0 | 0.327 | 0.796 |
| MAD2L1 | HMMR | 0.682 | 0.079 | 0 | 0.375 | 0.801 |
| MAD2L1 | KNL1 | 0.38 | 0.068 | 0.5 | 0.437 | 0.815 |
| MAD2L1 | CKS2 | 0.655 | 0 | 0 | 0.499 | 0.82 |
| MAD2L1 | UBE2T | 0.783 | 0.059 | 0 | 0.273 | 0.838 |
| MAD2L1 | CENPK | 0.618 | 0 | 0.5 | 0.268 | 0.848 |
| MAD2L1 | TPX2 | 0.543 | 0.075 | 0 | 0.701 | 0.863 |
| MAD2L1 | CENPA | 0.521 | 0.085 | 0.5 | 0.457 | 0.865 |
| MAD2L1 | CDKN3 | 0.78 | 0.226 | 0 | 0.297 | 0.87 |
| MAD2L1 | PTTG1 | 0.539 | 0 | 0 | 0.774 | 0.891 |
| MAD2L1 | ZWINT | 0.542 | 0.191 | 0.5 | 0.523 | 0.899 |
| MAD2L1 | CENPI | 0.44 | 0.306 | 0.5 | 0.549 | 0.9 |
| MAD2L1 | CENPF | 0.657 | 0 | 0.5 | 0.544 | 0.915 |
| MAD2L1 | CCNA2 | 0.849 | 0 | 0.5 | 0.853 | 0.988 |
| MAD2L1 | CDK1 | 0.857 | 0.113 | 0.5 | 0.896 | 0.992 |
| MAD2L1 | NEK2 | 0.59 | 0.994 | 0.5 | 0.487 | 0.999 |
| MTFR2 | NUF2 | 0.534 | 0 | 0 | 0.048 | 0.537 |
| MTFR2 | SPC25 | 0.616 | 0.071 | 0 | 0 | 0.628 |
| MTFR2 | CCNB2 | 0.248 | 0.1 | 0 | 0.191 | 0.405 |
| MTFR2 | SKA3 | 0.419 | 0 | 0 | 0.184 | 0.505 |
| MTFR2 | CDC25C | 0.337 | 0.045 | 0 | 0.128 | 0.4 |
| MTFR2 | CENPA | 0.337 | 0.073 | 0 | 0.231 | 0.486 |
| MTFR2 | CEP55 | 0.419 | 0 | 0 | 0.099 | 0.454 |
| MTFR2 | CDK1 | 0.348 | 0.09 | 0 | 0.1 | 0.42 |
| MTFR2 | DEPDC1 | 0.337 | 0 | 0 | 0.145 | 0.408 |
| MTFR2 | UBE2T | 0.293 | 0.064 | 0 | 0.204 | 0.427 |
| NCAPD2 | CDC6 | 0.426 | 0 | 0 | 0.47 | 0.683 |
| NCAPD2 | AURKA | 0.447 | 0.112 | 0 | 0.397 | 0.678 |
| NCAPD2 | GMNN | 0.189 | 0 | 0 | 0.3 | 0.409 |
| NCAPD2 | PIMREG | 0.253 | 0 | 0 | 0.476 | 0.592 |
| NCAPD2 | NCAPG | 0.799 | 0.978 | 0.9 | 0.995 | 0.999 |
| NCAPD2 | KIF11 | 0.752 | 0 | 0 | 0.461 | 0.86 |
| NCAPD2 | CENPE | 0.315 | 0 | 0 | 0.416 | 0.583 |
| NCAPD2 | NUF2 | 0.368 | 0 | 0 | 0.398 | 0.604 |
| NCAPD2 | SPC25 | 0.446 | 0 | 0 | 0.195 | 0.535 |
| NCAPD2 | CCNB2 | 0.439 | 0 | 0.4 | 0.287 | 0.739 |
| NCAPD2 | MAD2L1 | 0.37 | 0 | 0 | 0.267 | 0.519 |
| NCAPD2 | TPX2 | 0.478 | 0 | 0 | 0.266 | 0.6 |
| NCAPD2 | CKS1B | 0.229 | 0 | 0 | 0.39 | 0.51 |
| NCAPD2 | CDC25C | 0.292 | 0 | 0 | 0.211 | 0.418 |
| NCAPD2 | SPAG5 | 0.441 | 0 | 0 | 0.324 | 0.606 |
| NCAPD2 | KPNA2 | 0.294 | 0 | 0 | 0.191 | 0.405 |
| NCAPD2 | CENPK | 0.321 | 0.091 | 0 | 0.157 | 0.434 |
| NCAPD2 | KIF18B | 0.269 | 0 | 0 | 0.26 | 0.436 |
| NCAPD2 | ZWINT | 0.242 | 0 | 0 | 0.291 | 0.439 |
| NCAPD2 | CKS2 | 0.208 | 0 | 0 | 0.345 | 0.459 |
| NCAPD2 | NEK2 | 0.267 | 0 | 0 | 0.316 | 0.477 |
| NCAPD2 | HMMR | 0.332 | 0 | 0 | 0.264 | 0.487 |
| NCAPD2 | CEP55 | 0.348 | 0 | 0 | 0.249 | 0.49 |
| NCAPD2 | KNL1 | 0.224 | 0 | 0 | 0.381 | 0.499 |
| NCAPD2 | RAD51 | 0.376 | 0 | 0 | 0.267 | 0.523 |
| NCAPD2 | CENPA | 0.291 | 0 | 0 | 0.385 | 0.546 |
| NCAPD2 | PTTG1 | 0.279 | 0.124 | 0 | 0.392 | 0.583 |
| NCAPD2 | CENPF | 0.384 | 0 | 0 | 0.375 | 0.599 |
| NCAPD2 | CCNA2 | 0.485 | 0 | 0 | 0.29 | 0.619 |
| NCAPD2 | ECT2 | 0.226 | 0.292 | 0 | 0.371 | 0.625 |
| NCAPD2 | KIF4A | 0.541 | 0.094 | 0 | 0.393 | 0.725 |
| NCAPD2 | CDK1 | 0.298 | 0.292 | 0.4 | 0.375 | 0.789 |
| NCAPG | CDC6 | 0.477 | 0 | 0 | 0.39 | 0.667 |
| NCAPG | CENPM | 0.55 | 0 | 0 | 0.219 | 0.634 |
| NCAPG | AURKA | 0.816 | 0.103 | 0 | 0.735 | 0.952 |
| NCAPG | GMNN | 0.488 | 0 | 0 | 0.119 | 0.53 |
| NCAPG | KNSTRN | 0.415 | 0 | 0 | 0.17 | 0.494 |
| NCAPG | PIMREG | 0.439 | 0 | 0 | 0.434 | 0.669 |
| NCAPG | CKS1B | 0.305 | 0 | 0 | 0.22 | 0.434 |
| NCAPG | ASF1B | 0.385 | 0 | 0 | 0.193 | 0.483 |
| NCAPG | BORA | 0.463 | 0 | 0 | 0.083 | 0.487 |
| NCAPG | CDC25C | 0.48 | 0 | 0 | 0.194 | 0.563 |
| NCAPG | UBE2T | 0.503 | 0.059 | 0 | 0.162 | 0.574 |
| NCAPG | RAD51 | 0.501 | 0 | 0 | 0.189 | 0.578 |
| NCAPG | TK1 | 0.469 | 0 | 0 | 0.267 | 0.595 |
| NCAPG | KPNA2 | 0.475 | 0 | 0 | 0.294 | 0.614 |
| NCAPG | KIF18B | 0.378 | 0 | 0 | 0.423 | 0.626 |
| NCAPG | CENPK | 0.455 | 0 | 0 | 0.381 | 0.649 |
| NCAPG | KNL1 | 0.502 | 0 | 0 | 0.33 | 0.652 |
| NCAPG | CKS2 | 0.497 | 0 | 0 | 0.351 | 0.66 |
| NCAPG | RAD54L | 0.526 | 0.067 | 0 | 0.313 | 0.669 |
| NCAPG | CENPI | 0.38 | 0.113 | 0 | 0.471 | 0.684 |
| NCAPG | TROAP | 0.544 | 0 | 0 | 0.348 | 0.69 |
| NCAPG | SGO1 | 0.555 | 0.047 | 0 | 0.392 | 0.719 |
| NCAPG | DEPDC1B | 0.657 | 0 | 0 | 0.27 | 0.739 |
| NCAPG | SPAG5 | 0.607 | 0 | 0 | 0.372 | 0.742 |
| NCAPG | SKA3 | 0.69 | 0 | 0 | 0.316 | 0.779 |
| NCAPG | PTTG1 | 0.664 | 0 | 0 | 0.44 | 0.804 |
| NCAPG | CDKN3 | 0.792 | 0 | 0 | 0.15 | 0.816 |
| NCAPG | NEK2 | 0.589 | 0 | 0 | 0.587 | 0.823 |
| NCAPG | DEPDC1 | 0.784 | 0 | 0 | 0.22 | 0.824 |
| NCAPG | MAD2L1 | 0.574 | 0.088 | 0 | 0.614 | 0.837 |
| NCAPG | ZWINT | 0.657 | 0 | 0 | 0.587 | 0.852 |
| NCAPG | ECT2 | 0.813 | 0 | 0 | 0.307 | 0.865 |
| NCAPG | SPC25 | 0.839 | 0 | 0 | 0.205 | 0.866 |
| NCAPG | CENPA | 0.743 | 0.092 | 0 | 0.487 | 0.87 |
| NCAPG | CEP55 | 0.825 | 0 | 0 | 0.447 | 0.899 |
| NCAPG | HMMR | 0.817 | 0 | 0 | 0.523 | 0.909 |
| NCAPG | CENPE | 0.859 | 0 | 0 | 0.459 | 0.92 |
| NCAPG | CENPF | 0.827 | 0 | 0 | 0.582 | 0.924 |
| NCAPG | NUF2 | 0.865 | 0 | 0 | 0.505 | 0.93 |
| NCAPG | KIF4A | 0.843 | 0 | 0 | 0.599 | 0.934 |
| NCAPG | TPX2 | 0.827 | 0 | 0 | 0.72 | 0.949 |
| NCAPG | CCNA2 | 0.854 | 0 | 0 | 0.668 | 0.949 |
| NCAPG | CCNB2 | 0.823 | 0 | 0.4 | 0.742 | 0.97 |
| NCAPG | KIF11 | 0.912 | 0.086 | 0 | 0.722 | 0.975 |
| NCAPG | CDK1 | 0.866 | 0.292 | 0.4 | 0.708 | 0.981 |
| NCAPG | NCAPD2 | 0.799 | 0.978 | 0.9 | 0.995 | 0.999 |
| NEK2 | CDC6 | 0.446 | 0 | 0 | 0.392 | 0.648 |
| NEK2 | CENPM | 0.468 | 0 | 0 | 0.201 | 0.557 |
| NEK2 | AURKA | 0.641 | 0 | 0 | 0.501 | 0.829 |
| NEK2 | ORC6 | 0.178 | 0 | 0 | 0.454 | 0.533 |
| NEK2 | PIMREG | 0.379 | 0 | 0 | 0.359 | 0.586 |
| NEK2 | NCAPG | 0.589 | 0 | 0 | 0.587 | 0.823 |
| NEK2 | CCNA1 | 0.298 | 0 | 0 | 0.534 | 0.659 |
| NEK2 | TROAP | 0.478 | 0 | 0 | 0.423 | 0.686 |
| NEK2 | KIF11 | 0.845 | 0.054 | 0 | 0.745 | 0.959 |
| NEK2 | ASF1B | 0.351 | 0 | 0 | 0.167 | 0.436 |
| NEK2 | SGO1 | 0.748 | 0 | 0 | 0.43 | 0.85 |
| NEK2 | DEPDC1B | 0.682 | 0 | 0 | 0.165 | 0.723 |
| NEK2 | CENPE | 0.586 | 0 | 0 | 0.616 | 0.834 |
| NEK2 | NUF2 | 0.856 | 0 | 0 | 0.682 | 0.952 |
| NEK2 | SPC25 | 0.593 | 0 | 0 | 0.156 | 0.642 |
| NEK2 | CCNB2 | 0.67 | 0 | 0 | 0.717 | 0.902 |
| NEK2 | MAD2L1 | 0.59 | 0.994 | 0.5 | 0.487 | 0.999 |
| NEK2 | TPX2 | 0.818 | 0 | 0 | 0.696 | 0.942 |
| NEK2 | CKS1B | 0.323 | 0 | 0 | 0.435 | 0.602 |
| NEK2 | SKA3 | 0.391 | 0 | 0 | 0.262 | 0.532 |
| NEK2 | CDC25C | 0.652 | 0 | 0 | 0.486 | 0.814 |
| NEK2 | SPAG5 | 0.76 | 0.043 | 0 | 0.392 | 0.848 |
| NEK2 | NCAPD2 | 0.267 | 0 | 0 | 0.316 | 0.477 |
| NEK2 | CDKN3 | 0.67 | 0 | 0 | 0.191 | 0.722 |
| NEK2 | KNL1 | 0.373 | 0 | 0 | 0.321 | 0.556 |
| NEK2 | CENPA | 0.691 | 0 | 0 | 0.535 | 0.85 |
| NEK2 | CENPF | 0.882 | 0 | 0 | 0.759 | 0.97 |
| NEK2 | BRCA2 | 0.196 | 0 | 0 | 0.338 | 0.445 |
| NEK2 | CENPK | 0.323 | 0 | 0 | 0.248 | 0.469 |
| NEK2 | TK1 | 0.394 | 0 | 0 | 0.173 | 0.478 |
| NEK2 | RAD54L | 0.362 | 0.052 | 0 | 0.21 | 0.481 |
| NEK2 | RAD51 | 0.394 | 0 | 0 | 0.252 | 0.527 |
| NEK2 | KPNA2 | 0.439 | 0 | 0 | 0.193 | 0.528 |
| NEK2 | KIF18B | 0.447 | 0 | 0 | 0.201 | 0.539 |
| NEK2 | CKS2 | 0.321 | 0 | 0 | 0.392 | 0.569 |
| NEK2 | UBE2T | 0.438 | 0.093 | 0 | 0.246 | 0.582 |
| NEK2 | CENPI | 0.426 | 0 | 0 | 0.329 | 0.599 |
| NEK2 | BORA | 0.37 | 0 | 0.5 | 0.178 | 0.718 |
| NEK2 | ZWINT | 0.39 | 0 | 0 | 0.662 | 0.785 |
| NEK2 | ECT2 | 0.702 | 0 | 0 | 0.42 | 0.82 |
| NEK2 | DEPDC1 | 0.789 | 0 | 0 | 0.303 | 0.847 |
| NEK2 | KIF4A | 0.649 | 0 | 0 | 0.611 | 0.858 |
| NEK2 | HMMR | 0.779 | 0.043 | 0 | 0.436 | 0.87 |
| NEK2 | PTTG1 | 0.569 | 0 | 0 | 0.755 | 0.89 |
| NEK2 | CEP55 | 0.816 | 0 | 0 | 0.541 | 0.912 |
| NEK2 | CDK1 | 0.832 | 0 | 0 | 0.493 | 0.919 |
| NEK2 | CCNA2 | 0.829 | 0 | 0 | 0.681 | 0.943 |
| NEMP1 | DEPDC1B | 0.111 | 0 | 0 | 0.532 | 0.566 |
| NEMP1 | MAD2L1 | 0.2 | 0 | 0 | 0.304 | 0.419 |
| NUF2 | CDC6 | 0.566 | 0 | 0 | 0.374 | 0.717 |
| NUF2 | CENPM | 0.39 | 0 | 0 | 0.526 | 0.698 |
| NUF2 | AURKA | 0.674 | 0.292 | 0 | 0.574 | 0.893 |
| NUF2 | ORC6 | 0.282 | 0 | 0 | 0.322 | 0.493 |
| NUF2 | GMNN | 0.398 | 0 | 0 | 0.353 | 0.594 |
| NUF2 | KNSTRN | 0.681 | 0 | 0 | 0.541 | 0.847 |
| NUF2 | PIMREG | 0.411 | 0 | 0 | 0.509 | 0.698 |
| NUF2 | NCAPG | 0.865 | 0 | 0 | 0.505 | 0.93 |
| NUF2 | CCNA1 | 0.321 | 0 | 0 | 0.193 | 0.429 |
| NUF2 | TROAP | 0.375 | 0 | 0 | 0.254 | 0.514 |
| NUF2 | KIF11 | 0.925 | 0.084 | 0 | 0.669 | 0.975 |
| NUF2 | ASF1B | 0.388 | 0 | 0 | 0.2 | 0.489 |
| NUF2 | SGO1 | 0.74 | 0 | 0 | 0.478 | 0.858 |
| NUF2 | DEPDC1B | 0.799 | 0 | 0 | 0.095 | 0.81 |
| NUF2 | CENPE | 0.839 | 0.484 | 0 | 0.958 | 0.996 |
| NUF2 | BORA | 0.394 | 0 | 0 | 0.101 | 0.432 |
| NUF2 | RAD54L | 0.41 | 0.053 | 0 | 0.187 | 0.506 |
| NUF2 | KPNA2 | 0.484 | 0 | 0 | 0.128 | 0.531 |
| NUF2 | MTFR2 | 0.534 | 0 | 0 | 0.048 | 0.537 |
| NUF2 | NCAPD2 | 0.368 | 0 | 0 | 0.398 | 0.604 |
| NUF2 | CKS1B | 0.512 | 0 | 0 | 0.262 | 0.625 |
| NUF2 | CDC25C | 0.556 | 0 | 0 | 0.266 | 0.66 |
| NUF2 | TK1 | 0.525 | 0 | 0 | 0.383 | 0.694 |
| NUF2 | CKS2 | 0.554 | 0 | 0 | 0.358 | 0.701 |
| NUF2 | KIF18B | 0.386 | 0.06 | 0 | 0.568 | 0.729 |
| NUF2 | RAD51 | 0.749 | 0 | 0 | 0.19 | 0.788 |
| NUF2 | CENPK | 0.594 | 0 | 0 | 0.511 | 0.793 |
| NUF2 | SPAG5 | 0.648 | 0.076 | 0 | 0.447 | 0.804 |
| NUF2 | PTTG1 | 0.623 | 0 | 0 | 0.517 | 0.81 |
| NUF2 | ECT2 | 0.583 | 0 | 0 | 0.581 | 0.818 |
| NUF2 | CDKN3 | 0.811 | 0.064 | 0 | 0.264 | 0.858 |
| NUF2 | CENPI | 0.454 | 0.078 | 0 | 0.787 | 0.883 |
| NUF2 | HMMR | 0.817 | 0.053 | 0 | 0.395 | 0.886 |
| NUF2 | SKA3 | 0.642 | 0 | 0 | 0.713 | 0.893 |
| NUF2 | DEPDC1 | 0.802 | 0 | 0 | 0.506 | 0.898 |
| NUF2 | UBE2T | 0.801 | 0 | 0 | 0.521 | 0.901 |
| NUF2 | KIF4A | 0.695 | 0 | 0 | 0.721 | 0.911 |
| NUF2 | CCNB2 | 0.744 | 0 | 0.4 | 0.594 | 0.932 |
| NUF2 | CENPA | 0.618 | 0.058 | 0 | 0.836 | 0.936 |
| NUF2 | CCNA2 | 0.91 | 0 | 0 | 0.37 | 0.94 |
| NUF2 | CEP55 | 0.835 | 0 | 0 | 0.693 | 0.947 |
| NUF2 | TPX2 | 0.838 | 0 | 0 | 0.686 | 0.947 |
| NUF2 | NEK2 | 0.856 | 0 | 0 | 0.682 | 0.952 |
| NUF2 | CENPF | 0.875 | 0 | 0 | 0.632 | 0.952 |
| NUF2 | CDK1 | 0.867 | 0 | 0.4 | 0.51 | 0.957 |
| NUF2 | MAD2L1 | 0.91 | 0.107 | 0.5 | 0.581 | 0.981 |
| NUF2 | ZWINT | 0.641 | 0.887 | 0.54 | 0.606 | 0.991 |
| NUF2 | KNL1 | 0.513 | 0.827 | 0.54 | 0.823 | 0.992 |
| NUF2 | SPC25 | 0.862 | 0.995 | 0.9 | 0.947 | 0.999 |
| ORC6 | CDC6 | 0.261 | 0.927 | 0.9 | 0.989 | 0.999 |
| ORC6 | CENPM | 0.162 | 0 | 0 | 0.435 | 0.506 |
| ORC6 | SPC25 | 0.367 | 0 | 0 | 0.139 | 0.432 |
| ORC6 | CENPA | 0.185 | 0 | 0 | 0.345 | 0.444 |
| ORC6 | KIF11 | 0.256 | 0 | 0 | 0.329 | 0.48 |
| ORC6 | TK1 | 0.209 | 0 | 0 | 0.37 | 0.48 |
| ORC6 | NUF2 | 0.282 | 0 | 0 | 0.322 | 0.493 |
| ORC6 | CEP55 | 0.203 | 0 | 0 | 0.394 | 0.496 |
| ORC6 | RAD51 | 0.349 | 0 | 0 | 0.27 | 0.504 |
| ORC6 | CENPF | 0.234 | 0 | 0 | 0.413 | 0.531 |
| ORC6 | CCNB2 | 0.254 | 0 | 0 | 0.397 | 0.531 |
| ORC6 | ASF1B | 0.267 | 0 | 0 | 0.387 | 0.532 |
| ORC6 | NEK2 | 0.178 | 0 | 0 | 0.454 | 0.533 |
| ORC6 | RAD54L | 0.269 | 0 | 0 | 0.4 | 0.543 |
| ORC6 | EME1 | 0.184 | 0 | 0 | 0.465 | 0.545 |
| ORC6 | SPAG5 | 0.216 | 0.453 | 0 | 0.108 | 0.584 |
| ORC6 | CCNA1 | 0.086 | 0 | 0.4 | 0.327 | 0.599 |
| ORC6 | MAD2L1 | 0.48 | 0 | 0 | 0.34 | 0.642 |
| ORC6 | PTTG1 | 0.21 | 0 | 0 | 0.619 | 0.686 |
| ORC6 | CCNA2 | 0.251 | 0 | 0.4 | 0.429 | 0.721 |
| ORC6 | CDK1 | 0.284 | 0 | 0.5 | 0.34 | 0.743 |
| ORC6 | GMNN | 0.236 | 0 | 0.5 | 0.745 | 0.894 |
| PIMREG | CDC6 | 0.269 | 0 | 0 | 0.231 | 0.414 |
| PIMREG | CENPM | 0.409 | 0 | 0 | 0.238 | 0.53 |
| PIMREG | AURKA | 0.411 | 0 | 0 | 0.223 | 0.523 |
| PIMREG | KNSTRN | 0.384 | 0 | 0 | 0.125 | 0.438 |
| PIMREG | UBE2T | 0.341 | 0 | 0 | 0.149 | 0.415 |
| PIMREG | ASF1B | 0.372 | 0 | 0 | 0.114 | 0.42 |
| PIMREG | SGO1 | 0.32 | 0 | 0 | 0.2 | 0.433 |
| PIMREG | TK1 | 0.365 | 0 | 0 | 0.147 | 0.436 |
| PIMREG | CKS2 | 0.252 | 0 | 0 | 0.291 | 0.447 |
| PIMREG | CKS1B | 0.274 | 0 | 0 | 0.276 | 0.452 |
| PIMREG | ZWINT | 0.334 | 0 | 0 | 0.213 | 0.453 |
| PIMREG | ECT2 | 0.257 | 0 | 0 | 0.324 | 0.477 |
| PIMREG | CDKN3 | 0.464 | 0 | 0 | 0.086 | 0.489 |
| PIMREG | SKA3 | 0.36 | 0 | 0 | 0.257 | 0.504 |
| PIMREG | PTTG1 | 0.384 | 0 | 0 | 0.247 | 0.516 |
| PIMREG | SPAG5 | 0.466 | 0.1 | 0 | 0.097 | 0.528 |
| PIMREG | CCNA2 | 0.472 | 0 | 0 | 0.197 | 0.558 |
| PIMREG | KIF11 | 0.386 | 0 | 0 | 0.328 | 0.57 |
| PIMREG | CENPE | 0.385 | 0 | 0 | 0.347 | 0.582 |
| PIMREG | NEK2 | 0.379 | 0 | 0 | 0.359 | 0.586 |
| PIMREG | NCAPD2 | 0.253 | 0 | 0 | 0.476 | 0.592 |
| PIMREG | CDC25C | 0.473 | 0 | 0 | 0.258 | 0.593 |
| PIMREG | HMMR | 0.4 | 0 | 0 | 0.359 | 0.599 |
| PIMREG | TPX2 | 0.438 | 0 | 0 | 0.316 | 0.599 |
| PIMREG | DEPDC1 | 0.41 | 0 | 0 | 0.37 | 0.612 |
| PIMREG | TROAP | 0.436 | 0 | 0 | 0.359 | 0.623 |
| PIMREG | NCAPG | 0.439 | 0 | 0 | 0.434 | 0.669 |
| PIMREG | CENPF | 0.436 | 0 | 0 | 0.445 | 0.673 |
| PIMREG | CEP55 | 0.511 | 0 | 0 | 0.383 | 0.685 |
| PIMREG | KIF4A | 0.539 | 0 | 0 | 0.348 | 0.687 |
| PIMREG | NUF2 | 0.411 | 0 | 0 | 0.509 | 0.698 |
| PIMREG | CDK1 | 0.485 | 0 | 0 | 0.53 | 0.748 |
| PIMREG | CCNB2 | 0.465 | 0 | 0 | 0.559 | 0.754 |
| PIMREG | CENPA | 0.783 | 0 | 0 | 0.395 | 0.863 |
| PRR11 | CEP55 | 0.24 | 0 | 0 | 0.251 | 0.406 |
| PRR11 | CCNB2 | 0.313 | 0 | 0 | 0.187 | 0.418 |
| PRR11 | KIF18B | 0.393 | 0 | 0 | 0.116 | 0.44 |
| PSMC3IP | MAD2L1 | 0.366 | 0 | 0 | 0.129 | 0.424 |
| PSMC3IP | KNL1 | 0.144 | 0.11 | 0 | 0.306 | 0.425 |
| PSMC3IP | RAD54L | 0.293 | 0 | 0 | 0.27 | 0.462 |
| PSMC3IP | BRCA2 | 0.128 | 0 | 0.4 | 0.243 | 0.569 |
| PSMC3IP | RAD51 | 0.326 | 0.354 | 0.4 | 0.689 | 0.908 |
| PTTG1 | CDC6 | 0.331 | 0 | 0 | 0.528 | 0.671 |
| PTTG1 | CENPM | 0.498 | 0 | 0 | 0.37 | 0.67 |
| PTTG1 | AURKA | 0.68 | 0.329 | 0.5 | 0.73 | 0.967 |
| PTTG1 | ORC6 | 0.21 | 0 | 0 | 0.619 | 0.686 |
| PTTG1 | GMNN | 0.355 | 0 | 0 | 0.305 | 0.532 |
| PTTG1 | KNSTRN | 0.335 | 0 | 0 | 0.179 | 0.43 |
| PTTG1 | PIMREG | 0.384 | 0 | 0 | 0.247 | 0.516 |
| PTTG1 | NCAPG | 0.664 | 0 | 0 | 0.44 | 0.804 |
| PTTG1 | CCNA1 | 0.251 | 0 | 0 | 0.61 | 0.695 |
| PTTG1 | TROAP | 0.497 | 0 | 0 | 0.138 | 0.637 |
| PTTG1 | KIF11 | 0.569 | 0 | 0 | 0.687 | 0.859 |
| PTTG1 | ASF1B | 0.493 | 0 | 0 | 0.31 | 0.636 |
| PTTG1 | SGO1 | 0.443 | 0 | 0 | 0.789 | 0.877 |
| PTTG1 | CENPE | 0.505 | 0 | 0 | 0.606 | 0.796 |
| PTTG1 | NUF2 | 0.623 | 0 | 0 | 0.517 | 0.81 |
| PTTG1 | SPC25 | 0.486 | 0 | 0 | 0.248 | 0.597 |
| PTTG1 | CCNB2 | 0.863 | 0 | 0 | 0.79 | 0.97 |
| PTTG1 | MAD2L1 | 0.539 | 0 | 0 | 0.774 | 0.891 |
| PTTG1 | TPX2 | 0.77 | 0 | 0 | 0.673 | 0.921 |
| PTTG1 | CKS1B | 0.792 | 0 | 0 | 0.327 | 0.854 |
| PTTG1 | SKA3 | 0.395 | 0.126 | 0 | 0.229 | 0.557 |
| PTTG1 | CDC25C | 0.48 | 0 | 0 | 0.477 | 0.716 |
| PTTG1 | SPAG5 | 0.68 | 0 | 0 | 0.519 | 0.84 |
| PTTG1 | NCAPD2 | 0.279 | 0.124 | 0 | 0.392 | 0.583 |
| PTTG1 | CDKN3 | 0.8 | 0 | 0 | 0.23 | 0.84 |
| PTTG1 | KNL1 | 0.244 | 0 | 0 | 0.478 | 0.588 |
| PTTG1 | CENPA | 0.59 | 0 | 0 | 0.44 | 0.761 |
| PTTG1 | CENPF | 0.574 | 0 | 0 | 0.611 | 0.827 |
| PTTG1 | NEK2 | 0.569 | 0 | 0 | 0.755 | 0.89 |
| PTTG1 | CEP55 | 0.653 | 0 | 0 | 0.702 | 0.892 |
| PTTG1 | RAD54L | 0.309 | 0 | 0 | 0.357 | 0.537 |
| PTTG1 | ZWINT | 0.537 | 0 | 0 | 0.431 | 0.725 |
| PTTG1 | KIF4A | 0.535 | 0 | 0 | 0.559 | 0.786 |
| PTTG1 | CKS2 | 0.801 | 0 | 0 | 0.459 | 0.888 |
| PTTG1 | ECT2 | 0.404 | 0 | 0 | 0.394 | 0.623 |
| PTTG1 | HMMR | 0.781 | 0 | 0 | 0.391 | 0.861 |
| PTTG1 | KIF18B | 0.275 | 0 | 0 | 0.23 | 0.418 |
| PTTG1 | CENPK | 0.365 | 0.124 | 0 | 0.251 | 0.547 |
| PTTG1 | DEPDC1 | 0.412 | 0 | 0 | 0.29 | 0.565 |
| PTTG1 | RAD51 | 0.358 | 0.17 | 0 | 0.293 | 0.59 |
| PTTG1 | KPNA2 | 0.549 | 0 | 0 | 0.255 | 0.65 |
| PTTG1 | TK1 | 0.449 | 0 | 0 | 0.632 | 0.788 |
| PTTG1 | UBE2T | 0.786 | 0 | 0 | 0.348 | 0.854 |
| PTTG1 | CCNA2 | 0.808 | 0 | 0 | 0.672 | 0.934 |
| PTTG1 | CDK1 | 0.856 | 0 | 0 | 0.902 | 0.985 |
| RAD51 | CDC6 | 0.655 | 0 | 0 | 0.508 | 0.823 |
| RAD51 | CENPM | 0.424 | 0 | 0 | 0.249 | 0.549 |
| RAD51 | AURKA | 0.451 | 0.071 | 0 | 0.407 | 0.671 |
| RAD51 | ORC6 | 0.349 | 0 | 0 | 0.27 | 0.504 |
| RAD51 | GMNN | 0.322 | 0 | 0 | 0.208 | 0.44 |
| RAD51 | KNSTRN | 0.374 | 0 | 0 | 0.091 | 0.406 |
| RAD51 | NCAPG | 0.501 | 0 | 0 | 0.189 | 0.578 |
| RAD51 | CCNA1 | 0.236 | 0 | 0 | 0.421 | 0.539 |
| RAD51 | KIF11 | 0.596 | 0 | 0 | 0.276 | 0.695 |
| RAD51 | ASF1B | 0.415 | 0.129 | 0 | 0.43 | 0.684 |
| RAD51 | SGO1 | 0.467 | 0.162 | 0 | 0.162 | 0.593 |
| RAD51 | DEPDC1B | 0.349 | 0 | 0 | 0.144 | 0.419 |
| RAD51 | CENPE | 0.376 | 0 | 0 | 0.467 | 0.653 |
| RAD51 | NUF2 | 0.749 | 0 | 0 | 0.19 | 0.788 |
| RAD51 | SPC25 | 0.564 | 0 | 0 | 0.054 | 0.57 |
| RAD51 | CCNB2 | 0.522 | 0.189 | 0 | 0.552 | 0.811 |
| RAD51 | MAD2L1 | 0.695 | 0.088 | 0 | 0.327 | 0.796 |
| RAD51 | TPX2 | 0.479 | 0 | 0 | 0.473 | 0.714 |
| RAD51 | RMI2 | 0.39 | 0 | 0.5 | 0.571 | 0.858 |
| RAD51 | CKS1B | 0.298 | 0.067 | 0 | 0.18 | 0.416 |
| RAD51 | SKA3 | 0.446 | 0 | 0 | 0.184 | 0.529 |
| RAD51 | CDC25C | 0.361 | 0 | 0 | 0.384 | 0.59 |
| RAD51 | SPAG5 | 0.475 | 0.046 | 0 | 0.125 | 0.523 |
| RAD51 | NCAPD2 | 0.376 | 0 | 0 | 0.267 | 0.523 |
| RAD51 | CDKN3 | 0.346 | 0 | 0 | 0.215 | 0.465 |
| RAD51 | KNL1 | 0.37 | 0.31 | 0 | 0.091 | 0.589 |
| RAD51 | CENPA | 0.36 | 0.096 | 0 | 0.609 | 0.754 |
| RAD51 | CENPF | 0.372 | 0 | 0 | 0.329 | 0.561 |
| RAD51 | NEK2 | 0.394 | 0 | 0 | 0.252 | 0.527 |
| RAD51 | CEP55 | 0.518 | 0 | 0 | 0.296 | 0.646 |
| RAD51 | RAD54L | 0.737 | 0.709 | 0 | 0.903 | 0.992 |
| RAD51 | CENPI | 0.399 | 0 | 0 | 0.179 | 0.486 |
| RAD51 | ZWINT | 0.535 | 0 | 0 | 0.456 | 0.736 |
| RAD51 | KIF4A | 0.456 | 0 | 0 | 0.654 | 0.803 |
| RAD51 | CKS2 | 0.33 | 0.067 | 0 | 0.179 | 0.442 |
| RAD51 | BRCA2 | 0.28 | 0.996 | 0.9 | 0.999 | 0.999 |
| RAD51 | ECT2 | 0.446 | 0 | 0 | 0.153 | 0.511 |
| RAD51 | EME1 | 0.363 | 0.051 | 0.5 | 0.655 | 0.881 |
| RAD51 | PSMC3IP | 0.326 | 0.354 | 0.4 | 0.689 | 0.908 |
| RAD51 | HMMR | 0.444 | 0.046 | 0 | 0.216 | 0.548 |
| RAD51 | PTTG1 | 0.358 | 0.17 | 0 | 0.293 | 0.59 |
| RAD51 | CDK1 | 0.6 | 0.071 | 0 | 0.596 | 0.837 |
| RAD51 | CENPK | 0.448 | 0 | 0 | 0 | 0.448 |
| RAD51 | TONSL | 0.229 | 0 | 0 | 0.4 | 0.518 |
| RAD51 | DEPDC1 | 0.348 | 0 | 0 | 0.141 | 0.417 |
| RAD51 | KPNA2 | 0.442 | 0.046 | 0 | 0.148 | 0.507 |
| RAD51 | AUNIP | 0.368 | 0 | 0 | 0.144 | 0.436 |
| RAD51 | TK1 | 0.627 | 0 | 0 | 0.342 | 0.744 |
| RAD51 | CCNA2 | 0.616 | 0 | 0 | 0.479 | 0.791 |
| RAD51 | UBE2T | 0.436 | 0.114 | 0 | 0.5 | 0.728 |
| RAD54L | CDC6 | 0.43 | 0.091 | 0 | 0.292 | 0.601 |
| RAD54L | CENPM | 0.458 | 0 | 0 | 0.452 | 0.69 |
| RAD54L | AURKA | 0.377 | 0.051 | 0 | 0.252 | 0.519 |
| RAD54L | ORC6 | 0.269 | 0 | 0 | 0.4 | 0.543 |
| RAD54L | NCAPG | 0.526 | 0.067 | 0 | 0.313 | 0.669 |
| RAD54L | TROAP | 0.478 | 0 | 0 | 0.087 | 0.503 |
| RAD54L | KIF11 | 0.488 | 0.068 | 0 | 0.339 | 0.657 |
| RAD54L | ASF1B | 0.364 | 0.097 | 0 | 0.45 | 0.657 |
| RAD54L | CENPE | 0.35 | 0.066 | 0 | 0.186 | 0.463 |
| RAD54L | NUF2 | 0.41 | 0.053 | 0 | 0.187 | 0.506 |
| RAD54L | SPC25 | 0.449 | 0.053 | 0 | 0.125 | 0.503 |
| RAD54L | CCNB2 | 0.4 | 0.094 | 0 | 0.345 | 0.613 |
| RAD54L | MAD2L1 | 0.358 | 0.096 | 0 | 0.255 | 0.53 |
| RAD54L | TPX2 | 0.457 | 0.045 | 0 | 0.35 | 0.633 |
| RAD54L | RMI2 | 0.321 | 0 | 0 | 0.442 | 0.605 |
| RAD54L | SKA3 | 0.493 | 0.046 | 0 | 0.23 | 0.595 |
| RAD54L | CDC25C | 0.425 | 0.067 | 0 | 0.27 | 0.574 |
| RAD54L | SPAG5 | 0.562 | 0.099 | 0 | 0.216 | 0.664 |
| RAD54L | KNL1 | 0.29 | 0.297 | 0 | 0.191 | 0.561 |
| RAD54L | CENPA | 0.361 | 0.15 | 0 | 0.239 | 0.551 |
| RAD54L | CENPF | 0.382 | 0 | 0 | 0.382 | 0.602 |
| RAD54L | NEK2 | 0.362 | 0.052 | 0 | 0.21 | 0.481 |
| RAD54L | CEP55 | 0.4 | 0 | 0 | 0.391 | 0.619 |
| RAD54L | AUNIP | 0.345 | 0 | 0 | 0.134 | 0.409 |
| RAD54L | CENPK | 0.284 | 0.066 | 0 | 0.196 | 0.415 |
| RAD54L | DEPDC1 | 0.343 | 0.059 | 0 | 0.152 | 0.43 |
| RAD54L | KPNA2 | 0.295 | 0.134 | 0 | 0.151 | 0.437 |
| RAD54L | CENPI | 0.268 | 0.131 | 0 | 0.219 | 0.46 |
| RAD54L | PSMC3IP | 0.293 | 0 | 0 | 0.27 | 0.462 |
| RAD54L | ZWINT | 0.393 | 0 | 0 | 0.261 | 0.532 |
| RAD54L | HMMR | 0.378 | 0.099 | 0 | 0.232 | 0.532 |
| RAD54L | PTTG1 | 0.309 | 0 | 0 | 0.357 | 0.537 |
| RAD54L | KIF18B | 0.259 | 0.066 | 0 | 0.409 | 0.555 |
| RAD54L | UBE2T | 0.342 | 0.099 | 0 | 0.331 | 0.569 |
| RAD54L | CCNA2 | 0.461 | 0 | 0 | 0.269 | 0.589 |
| RAD54L | TK1 | 0.367 | 0 | 0 | 0.386 | 0.596 |
| RAD54L | KIF4A | 0.416 | 0.065 | 0 | 0.325 | 0.599 |
| RAD54L | EME1 | 0.27 | 0.334 | 0 | 0.517 | 0.745 |
| RAD54L | CDK1 | 0.438 | 0.107 | 0 | 0.596 | 0.78 |
| RAD54L | BRCA2 | 0.219 | 0.073 | 0 | 0.723 | 0.782 |
| RAD54L | RAD51 | 0.737 | 0.709 | 0 | 0.903 | 0.992 |
| RAG1 | EME1 | 0.06 | 0.047 | 0.54 | 0.09 | 0.575 |
| RAG1 | KPNA2 | 0.047 | 0.466 | 0 | 0.619 | 0.789 |
| RMI2 | TPX2 | 0.378 | 0 | 0 | 0.088 | 0.408 |
| RMI2 | TK1 | 0.323 | 0 | 0 | 0.163 | 0.409 |
| RMI2 | CCNA2 | 0.326 | 0 | 0 | 0.17 | 0.417 |
| RMI2 | CDK1 | 0.324 | 0 | 0 | 0.235 | 0.461 |
| RMI2 | RAD54L | 0.321 | 0 | 0 | 0.442 | 0.605 |
| RMI2 | UBE2T | 0.462 | 0 | 0 | 0.455 | 0.694 |
| RMI2 | BRCA2 | 0.092 | 0 | 0.5 | 0.426 | 0.717 |
| RMI2 | EME1 | 0.191 | 0 | 0.5 | 0.596 | 0.822 |
| RMI2 | RAD51 | 0.39 | 0 | 0.5 | 0.571 | 0.858 |
| SGO1 | CDC6 | 0.38 | 0 | 0 | 0.252 | 0.516 |
| SGO1 | CENPM | 0.304 | 0.131 | 0 | 0.22 | 0.487 |
| SGO1 | AURKA | 0.634 | 0 | 0 | 0.345 | 0.75 |
| SGO1 | KNSTRN | 0.371 | 0 | 0 | 0.276 | 0.525 |
| SGO1 | PIMREG | 0.32 | 0 | 0 | 0.2 | 0.433 |
| SGO1 | NCAPG | 0.555 | 0.047 | 0 | 0.392 | 0.719 |
| SGO1 | CCNA1 | 0.401 | 0 | 0 | 0.215 | 0.51 |
| SGO1 | TROAP | 0.412 | 0 | 0 | 0.077 | 0.434 |
| SGO1 | KIF11 | 0.714 | 0 | 0 | 0.467 | 0.841 |
| SGO1 | ASF1B | 0.418 | 0 | 0 | 0.207 | 0.518 |
| SGO1 | ARHGEF39 | 0.403 | 0 | 0 | 0 | 0.403 |
| SGO1 | ZWINT | 0.127 | 0 | 0 | 0.363 | 0.42 |
| SGO1 | KPNA2 | 0.386 | 0.094 | 0 | 0.07 | 0.438 |
| SGO1 | BORA | 0.312 | 0 | 0 | 0.252 | 0.464 |
| SGO1 | HMMR | 0.385 | 0 | 0 | 0.17 | 0.468 |
| SGO1 | DEPDC1B | 0.45 | 0 | 0 | 0.097 | 0.482 |
| SGO1 | DEPDC1 | 0.427 | 0 | 0 | 0.16 | 0.498 |
| SGO1 | ECT2 | 0.469 | 0 | 0 | 0.166 | 0.538 |
| SGO1 | CENPK | 0.346 | 0.143 | 0 | 0.245 | 0.54 |
| SGO1 | CDC25C | 0.462 | 0 | 0 | 0.191 | 0.546 |
| SGO1 | KIF18B | 0.383 | 0.068 | 0 | 0.348 | 0.592 |
| SGO1 | RAD51 | 0.467 | 0.162 | 0 | 0.162 | 0.593 |
| SGO1 | TPX2 | 0.457 | 0 | 0 | 0.314 | 0.611 |
| SGO1 | SPC25 | 0.478 | 0 | 0 | 0.308 | 0.623 |
| SGO1 | KIF4A | 0.519 | 0 | 0 | 0.397 | 0.698 |
| SGO1 | SKA3 | 0.49 | 0.171 | 0 | 0.381 | 0.716 |
| SGO1 | CEP55 | 0.623 | 0 | 0 | 0.355 | 0.746 |
| SGO1 | CENPF | 0.58 | 0 | 0 | 0.419 | 0.746 |
| SGO1 | CCNA2 | 0.698 | 0 | 0 | 0.317 | 0.785 |
| SGO1 | KNL1 | 0.526 | 0 | 0 | 0.583 | 0.793 |
| SGO1 | SPAG5 | 0.657 | 0.099 | 0 | 0.42 | 0.805 |
| SGO1 | CENPE | 0.566 | 0.091 | 0 | 0.61 | 0.833 |
| SGO1 | CCNB2 | 0.526 | 0.045 | 0.4 | 0.464 | 0.835 |
| SGO1 | NEK2 | 0.748 | 0 | 0 | 0.43 | 0.85 |
| SGO1 | CENPI | 0.615 | 0.099 | 0 | 0.617 | 0.856 |
| SGO1 | NUF2 | 0.74 | 0 | 0 | 0.478 | 0.858 |
| SGO1 | PTTG1 | 0.443 | 0 | 0 | 0.789 | 0.877 |
| SGO1 | CENPA | 0.574 | 0.042 | 0 | 0.73 | 0.88 |
| SGO1 | CDK1 | 0.668 | 0.128 | 0.4 | 0.501 | 0.902 |
| SGO1 | MAD2L1 | 0.305 | 0.1 | 0.5 | 0.734 | 0.905 |
| SKA3 | CDC6 | 0.465 | 0 | 0 | 0.174 | 0.539 |
| SKA3 | CENPM | 0.304 | 0.097 | 0 | 0.197 | 0.451 |
| SKA3 | AURKA | 0.452 | 0 | 0 | 0.199 | 0.542 |
| SKA3 | KNSTRN | 0.403 | 0 | 0 | 0.45 | 0.658 |
| SKA3 | PIMREG | 0.36 | 0 | 0 | 0.257 | 0.504 |
| SKA3 | NCAPG | 0.69 | 0 | 0 | 0.316 | 0.779 |
| SKA3 | TROAP | 0.404 | 0 | 0 | 0.199 | 0.502 |
| SKA3 | KIF11 | 0.559 | 0 | 0 | 0.292 | 0.675 |
| SKA3 | ASF1B | 0.381 | 0 | 0 | 0.071 | 0.4 |
| SKA3 | SGO1 | 0.49 | 0.171 | 0 | 0.381 | 0.716 |
| SKA3 | DEPDC1B | 0.567 | 0 | 0 | 0.117 | 0.601 |
| SKA3 | CENPE | 0.556 | 0 | 0 | 0.679 | 0.851 |
| SKA3 | NUF2 | 0.642 | 0 | 0 | 0.713 | 0.893 |
| SKA3 | SPC25 | 0.548 | 0 | 0 | 0.514 | 0.771 |
| SKA3 | CCNB2 | 0.568 | 0 | 0 | 0.225 | 0.651 |
| SKA3 | MAD2L1 | 0.445 | 0 | 0 | 0.232 | 0.555 |
| SKA3 | TPX2 | 0.529 | 0 | 0 | 0.576 | 0.791 |
| SKA3 | BORA | 0.411 | 0 | 0 | 0 | 0.411 |
| SKA3 | TK1 | 0.407 | 0 | 0 | 0.068 | 0.424 |
| SKA3 | CKS2 | 0.34 | 0 | 0 | 0.199 | 0.449 |
| SKA3 | CDKN3 | 0.462 | 0 | 0 | 0.046 | 0.465 |
| SKA3 | AUNIP | 0.356 | 0 | 0 | 0.22 | 0.477 |
| SKA3 | ECT2 | 0.332 | 0.198 | 0 | 0.145 | 0.502 |
| SKA3 | MTFR2 | 0.419 | 0 | 0 | 0.184 | 0.505 |
| SKA3 | KIF18B | 0.361 | 0 | 0 | 0.271 | 0.514 |
| SKA3 | RAD51 | 0.446 | 0 | 0 | 0.184 | 0.529 |
| SKA3 | NEK2 | 0.391 | 0 | 0 | 0.262 | 0.532 |
| SKA3 | UBE2T | 0.461 | 0 | 0 | 0.169 | 0.533 |
| SKA3 | PTTG1 | 0.395 | 0.126 | 0 | 0.229 | 0.557 |
| SKA3 | SPAG5 | 0.437 | 0 | 0 | 0.263 | 0.568 |
| SKA3 | CENPK | 0.374 | 0.137 | 0 | 0.29 | 0.583 |
| SKA3 | CDC25C | 0.513 | 0 | 0 | 0.189 | 0.588 |
| SKA3 | RAD54L | 0.493 | 0.046 | 0 | 0.23 | 0.595 |
| SKA3 | CENPI | 0.499 | 0 | 0 | 0.248 | 0.607 |
| SKA3 | CEP55 | 0.543 | 0 | 0 | 0.22 | 0.628 |
| SKA3 | CENPA | 0.47 | 0 | 0 | 0.38 | 0.657 |
| SKA3 | DEPDC1 | 0.512 | 0 | 0 | 0.439 | 0.714 |
| SKA3 | ZWINT | 0.495 | 0.172 | 0 | 0.398 | 0.726 |
| SKA3 | CCNA2 | 0.528 | 0 | 0 | 0.559 | 0.783 |
| SKA3 | CDK1 | 0.681 | 0 | 0 | 0.402 | 0.801 |
| SKA3 | KIF4A | 0.676 | 0 | 0 | 0.445 | 0.813 |
| SKA3 | KNL1 | 0.39 | 0 | 0 | 0.713 | 0.818 |
| SKA3 | HMMR | 0.652 | 0 | 0 | 0.642 | 0.87 |
| SKA3 | CENPF | 0.461 | 0 | 0.9 | 0.458 | 0.968 |
| SPAG5 | CDC6 | 0.48 | 0 | 0 | 0.193 | 0.562 |
| SPAG5 | CENPM | 0.358 | 0 | 0 | 0.248 | 0.497 |
| SPAG5 | AURKA | 0.581 | 0.294 | 0 | 0.376 | 0.8 |
| SPAG5 | ORC6 | 0.216 | 0.453 | 0 | 0.108 | 0.584 |
| SPAG5 | GMNN | 0.353 | 0 | 0 | 0.153 | 0.429 |
| SPAG5 | KNSTRN | 0.594 | 0.435 | 0 | 0.934 | 0.983 |
| SPAG5 | PIMREG | 0.466 | 0.1 | 0 | 0.097 | 0.528 |
| SPAG5 | NCAPG | 0.607 | 0 | 0 | 0.372 | 0.742 |
| SPAG5 | CCNA1 | 0.545 | 0 | 0 | 0.128 | 0.587 |
| SPAG5 | TROAP | 0.732 | 0 | 0 | 0.332 | 0.813 |
| SPAG5 | KIF11 | 0.883 | 0.071 | 0 | 0.613 | 0.954 |
| SPAG5 | ASF1B | 0.638 | 0.113 | 0 | 0.081 | 0.679 |
| SPAG5 | SGO1 | 0.657 | 0.099 | 0 | 0.42 | 0.805 |
| SPAG5 | DEPDC1B | 0.51 | 0.049 | 0 | 0.091 | 0.539 |
| SPAG5 | CENPE | 0.664 | 0.091 | 0 | 0.431 | 0.811 |
| SPAG5 | NUF2 | 0.648 | 0.076 | 0 | 0.447 | 0.804 |
| SPAG5 | SPC25 | 0.488 | 0.053 | 0 | 0.147 | 0.551 |
| SPAG5 | CCNB2 | 0.586 | 0.146 | 0 | 0.376 | 0.76 |
| SPAG5 | MAD2L1 | 0.407 | 0.096 | 0 | 0.562 | 0.745 |
| SPAG5 | TPX2 | 0.745 | 0.091 | 0 | 0.421 | 0.854 |
| SPAG5 | SKA3 | 0.437 | 0 | 0 | 0.263 | 0.568 |
| SPAG5 | CDC25C | 0.464 | 0 | 0 | 0.287 | 0.602 |
| SPAG5 | BORA | 0.254 | 0 | 0 | 0.24 | 0.409 |
| SPAG5 | EME1 | 0.284 | 0.105 | 0 | 0.153 | 0.41 |
| SPAG5 | ECT2 | 0.444 | 0.045 | 0 | 0.085 | 0.472 |
| SPAG5 | CENPI | 0.275 | 0.128 | 0 | 0.289 | 0.512 |
| SPAG5 | CKS2 | 0.379 | 0 | 0 | 0.261 | 0.522 |
| SPAG5 | RAD51 | 0.475 | 0.046 | 0 | 0.125 | 0.523 |
| SPAG5 | KPNA2 | 0.458 | 0.105 | 0 | 0.126 | 0.539 |
| SPAG5 | KNL1 | 0.367 | 0 | 0 | 0.352 | 0.572 |
| SPAG5 | CDKN3 | 0.524 | 0.089 | 0 | 0.117 | 0.584 |
| SPAG5 | DEPDC1 | 0.507 | 0.049 | 0 | 0.223 | 0.604 |
| SPAG5 | NCAPD2 | 0.441 | 0 | 0 | 0.324 | 0.606 |
| SPAG5 | UBE2T | 0.613 | 0.08 | 0 | 0.05 | 0.632 |
| SPAG5 | RAD54L | 0.562 | 0.099 | 0 | 0.216 | 0.664 |
| SPAG5 | ZWINT | 0.554 | 0 | 0 | 0.282 | 0.666 |
| SPAG5 | HMMR | 0.647 | 0 | 0 | 0.186 | 0.701 |
| SPAG5 | KIF18B | 0.563 | 0.057 | 0 | 0.37 | 0.718 |
| SPAG5 | TK1 | 0.506 | 0 | 0 | 0.47 | 0.727 |
| SPAG5 | CENPA | 0.746 | 0.094 | 0 | 0.3 | 0.825 |
| SPAG5 | PTTG1 | 0.68 | 0 | 0 | 0.519 | 0.84 |
| SPAG5 | NEK2 | 0.76 | 0.043 | 0 | 0.392 | 0.848 |
| SPAG5 | CEP55 | 0.743 | 0 | 0 | 0.537 | 0.876 |
| SPAG5 | KIF4A | 0.809 | 0.091 | 0 | 0.406 | 0.887 |
| SPAG5 | CCNA2 | 0.828 | 0 | 0 | 0.451 | 0.901 |
| SPAG5 | CENPF | 0.838 | 0.045 | 0 | 0.436 | 0.905 |
| SPAG5 | CDK1 | 0.761 | 0.354 | 0 | 0.566 | 0.927 |
| SPC25 | CDC6 | 0.471 | 0 | 0 | 0 | 0.471 |
| SPC25 | CENPM | 0.544 | 0 | 0 | 0.159 | 0.6 |
| SPC25 | AURKA | 0.474 | 0 | 0 | 0.165 | 0.542 |
| SPC25 | ORC6 | 0.367 | 0 | 0 | 0.139 | 0.432 |
| SPC25 | KNSTRN | 0.408 | 0 | 0 | 0 | 0.408 |
| SPC25 | NCAPG | 0.839 | 0 | 0 | 0.205 | 0.866 |
| SPC25 | TROAP | 0.331 | 0 | 0 | 0.187 | 0.432 |
| SPC25 | KIF11 | 0.785 | 0 | 0 | 0.088 | 0.796 |
| SPC25 | ASF1B | 0.374 | 0 | 0 | 0.148 | 0.444 |
| SPC25 | SGO1 | 0.478 | 0 | 0 | 0.308 | 0.623 |
| SPC25 | DEPDC1B | 0.573 | 0 | 0 | 0 | 0.573 |
| SPC25 | CENPE | 0.599 | 0 | 0 | 0.191 | 0.662 |
| SPC25 | NUF2 | 0.862 | 0.995 | 0.9 | 0.947 | 0.999 |
| SPC25 | KIF18B | 0.367 | 0 | 0 | 0.091 | 0.4 |
| SPC25 | CKS2 | 0.404 | 0 | 0 | 0 | 0.404 |
| SPC25 | CENPI | 0.372 | 0 | 0 | 0.135 | 0.433 |
| SPC25 | CDC25C | 0.463 | 0 | 0 | 0.069 | 0.478 |
| SPC25 | BORA | 0.364 | 0 | 0 | 0.216 | 0.48 |
| SPC25 | ECT2 | 0.5 | 0 | 0 | 0 | 0.5 |
| SPC25 | RAD54L | 0.449 | 0.053 | 0 | 0.125 | 0.503 |
| SPC25 | NCAPD2 | 0.446 | 0 | 0 | 0.195 | 0.535 |
| SPC25 | UBE2T | 0.478 | 0.069 | 0 | 0.134 | 0.542 |
| SPC25 | SPAG5 | 0.488 | 0.053 | 0 | 0.147 | 0.551 |
| SPC25 | RAD51 | 0.564 | 0 | 0 | 0.054 | 0.57 |
| SPC25 | PTTG1 | 0.486 | 0 | 0 | 0.248 | 0.597 |
| SPC25 | MTFR2 | 0.616 | 0.071 | 0 | 0 | 0.628 |
| SPC25 | NEK2 | 0.593 | 0 | 0 | 0.156 | 0.642 |
| SPC25 | CENPK | 0.593 | 0 | 0 | 0.175 | 0.649 |
| SPC25 | TK1 | 0.689 | 0 | 0 | 0 | 0.689 |
| SPC25 | CENPF | 0.668 | 0 | 0 | 0.232 | 0.734 |
| SPC25 | CCNB2 | 0.493 | 0 | 0.4 | 0.207 | 0.738 |
| SPC25 | DEPDC1 | 0.756 | 0 | 0 | 0 | 0.756 |
| SPC25 | KIF4A | 0.718 | 0 | 0 | 0.185 | 0.761 |
| SPC25 | SKA3 | 0.548 | 0 | 0 | 0.514 | 0.771 |
| SPC25 | CENPA | 0.779 | 0 | 0 | 0.148 | 0.804 |
| SPC25 | HMMR | 0.786 | 0.053 | 0 | 0.112 | 0.804 |
| SPC25 | CCNA2 | 0.805 | 0 | 0 | 0.068 | 0.811 |
| SPC25 | CEP55 | 0.795 | 0 | 0 | 0.117 | 0.811 |
| SPC25 | CDKN3 | 0.78 | 0 | 0 | 0.2 | 0.817 |
| SPC25 | TPX2 | 0.812 | 0 | 0 | 0.167 | 0.837 |
| SPC25 | CDK1 | 0.832 | 0 | 0.4 | 0.046 | 0.895 |
| SPC25 | MAD2L1 | 0.737 | 0 | 0.5 | 0.395 | 0.913 |
| SPC25 | KNL1 | 0.394 | 0.845 | 0.54 | 0.142 | 0.958 |
| SPC25 | ZWINT | 0.665 | 0.88 | 0.54 | 0.281 | 0.984 |
| TK1 | CDC6 | 0.542 | 0 | 0 | 0.348 | 0.689 |
| TK1 | CENPM | 0.547 | 0 | 0 | 0.451 | 0.74 |
| TK1 | AURKA | 0.43 | 0 | 0 | 0.325 | 0.598 |
| TK1 | ORC6 | 0.209 | 0 | 0 | 0.37 | 0.48 |
| TK1 | GMNN | 0.297 | 0 | 0 | 0.206 | 0.418 |
| TK1 | PIMREG | 0.365 | 0 | 0 | 0.147 | 0.436 |
| TK1 | NCAPG | 0.469 | 0 | 0 | 0.267 | 0.595 |
| TK1 | TROAP | 0.332 | 0 | 0 | 0.185 | 0.432 |
| TK1 | KIF11 | 0.483 | 0 | 0 | 0.557 | 0.761 |
| TK1 | ASF1B | 0.436 | 0 | 0 | 0.401 | 0.648 |
| TK1 | CENPE | 0.365 | 0 | 0 | 0.151 | 0.438 |
| TK1 | NUF2 | 0.525 | 0 | 0 | 0.383 | 0.694 |
| TK1 | SPC25 | 0.689 | 0 | 0 | 0 | 0.689 |
| TK1 | CCNB2 | 0.728 | 0 | 0 | 0.578 | 0.88 |
| TK1 | MAD2L1 | 0.452 | 0 | 0 | 0.5 | 0.714 |
| TK1 | TPX2 | 0.532 | 0.07 | 0 | 0.56 | 0.792 |
| TK1 | RMI2 | 0.323 | 0 | 0 | 0.163 | 0.409 |
| TK1 | CKS1B | 0.383 | 0 | 0 | 0.185 | 0.476 |
| TK1 | SKA3 | 0.407 | 0 | 0 | 0.068 | 0.424 |
| TK1 | CDC25C | 0.361 | 0 | 0 | 0.164 | 0.443 |
| TK1 | SPAG5 | 0.506 | 0 | 0 | 0.47 | 0.727 |
| TK1 | CDKN3 | 0.486 | 0 | 0 | 0.205 | 0.574 |
| TK1 | KNL1 | 0.298 | 0 | 0 | 0.18 | 0.4 |
| TK1 | CENPA | 0.407 | 0 | 0 | 0.247 | 0.534 |
| TK1 | CENPF | 0.434 | 0 | 0 | 0.63 | 0.782 |
| TK1 | NEK2 | 0.394 | 0 | 0 | 0.173 | 0.478 |
| TK1 | CEP55 | 0.498 | 0 | 0 | 0.622 | 0.802 |
| TK1 | RAD54L | 0.367 | 0 | 0 | 0.386 | 0.596 |
| TK1 | ZWINT | 0.776 | 0 | 0 | 0.567 | 0.898 |
| TK1 | KIF4A | 0.521 | 0 | 0 | 0.436 | 0.718 |
| TK1 | CKS2 | 0.354 | 0 | 0 | 0.486 | 0.653 |
| TK1 | ECT2 | 0.23 | 0 | 0 | 0.446 | 0.555 |
| TK1 | HMMR | 0.454 | 0 | 0 | 0.222 | 0.557 |
| TK1 | PTTG1 | 0.449 | 0 | 0 | 0.632 | 0.788 |
| TK1 | CDK1 | 0.576 | 0 | 0 | 0.638 | 0.84 |
| TK1 | CENPK | 0.304 | 0 | 0 | 0.188 | 0.41 |
| TK1 | DEPDC1 | 0.357 | 0 | 0 | 0.182 | 0.452 |
| TK1 | KPNA2 | 0.675 | 0 | 0 | 0.137 | 0.707 |
| TK1 | KIF18B | 0.44 | 0 | 0 | 0.069 | 0.456 |
| TK1 | UBE2T | 0.478 | 0 | 0 | 0.459 | 0.705 |
| TK1 | RAD51 | 0.627 | 0 | 0 | 0.342 | 0.744 |
| TK1 | CCNA2 | 0.482 | 0 | 0 | 0.593 | 0.78 |
| TONSL | ASF1B | 0.153 | 0.795 | 0 | 0.626 | 0.929 |
| TONSL | CENPI | 0.241 | 0.321 | 0 | 0.08 | 0.485 |
| TONSL | KIF18B | 0.487 | 0.057 | 0 | 0 | 0.495 |
| TONSL | RAD51 | 0.229 | 0 | 0 | 0.4 | 0.518 |
| TPX2 | CDC6 | 0.539 | 0 | 0 | 0.338 | 0.681 |
| TPX2 | CENPM | 0.478 | 0 | 0 | 0.333 | 0.637 |
| TPX2 | AURKA | 0.846 | 0.988 | 0.9 | 0.995 | 0.999 |
| TPX2 | KNSTRN | 0.341 | 0 | 0 | 0.145 | 0.413 |
| TPX2 | PIMREG | 0.438 | 0 | 0 | 0.316 | 0.599 |
| TPX2 | NCAPG | 0.827 | 0 | 0 | 0.72 | 0.949 |
| TPX2 | TROAP | 0.572 | 0 | 0 | 0.274 | 0.676 |
| TPX2 | KIF11 | 0.843 | 0 | 0 | 0.79 | 0.965 |
| TPX2 | ASF1B | 0.458 | 0 | 0 | 0.284 | 0.596 |
| TPX2 | SGO1 | 0.457 | 0 | 0 | 0.314 | 0.611 |
| TPX2 | DEPDC1B | 0.649 | 0 | 0 | 0.067 | 0.659 |
| TPX2 | CENPE | 0.804 | 0 | 0 | 0.48 | 0.894 |
| TPX2 | NUF2 | 0.838 | 0 | 0 | 0.686 | 0.947 |
| TPX2 | SPC25 | 0.812 | 0 | 0 | 0.167 | 0.837 |
| TPX2 | CCNB2 | 0.835 | 0.045 | 0 | 0.725 | 0.953 |
| TPX2 | MAD2L1 | 0.543 | 0.075 | 0 | 0.701 | 0.863 |
| TPX2 | RMI2 | 0.378 | 0 | 0 | 0.088 | 0.408 |
| TPX2 | CENPK | 0.367 | 0 | 0 | 0.284 | 0.528 |
| TPX2 | BRCA2 | 0.217 | 0 | 0 | 0.431 | 0.535 |
| TPX2 | CENPI | 0.406 | 0.045 | 0 | 0.246 | 0.535 |
| TPX2 | KNL1 | 0.406 | 0 | 0 | 0.275 | 0.551 |
| TPX2 | CKS1B | 0.397 | 0 | 0 | 0.31 | 0.566 |
| TPX2 | CKS2 | 0.455 | 0 | 0 | 0.29 | 0.596 |
| TPX2 | NCAPD2 | 0.478 | 0 | 0 | 0.266 | 0.6 |
| TPX2 | RAD54L | 0.457 | 0.045 | 0 | 0.35 | 0.633 |
| TPX2 | CDC25C | 0.541 | 0 | 0 | 0.256 | 0.644 |
| TPX2 | KIF18B | 0.42 | 0 | 0 | 0.476 | 0.683 |
| TPX2 | RAD51 | 0.479 | 0 | 0 | 0.473 | 0.714 |
| TPX2 | BORA | 0.313 | 0 | 0 | 0.624 | 0.73 |
| TPX2 | ECT2 | 0.518 | 0 | 0 | 0.503 | 0.75 |
| TPX2 | SKA3 | 0.529 | 0 | 0 | 0.576 | 0.791 |
| TPX2 | TK1 | 0.532 | 0.07 | 0 | 0.56 | 0.792 |
| TPX2 | UBE2T | 0.559 | 0.042 | 0 | 0.562 | 0.799 |
| TPX2 | CDKN3 | 0.787 | 0.044 | 0 | 0.178 | 0.818 |
| TPX2 | SPAG5 | 0.745 | 0.091 | 0 | 0.421 | 0.854 |
| TPX2 | KPNA2 | 0.611 | 0.297 | 0 | 0.552 | 0.867 |
| TPX2 | CENPA | 0.791 | 0 | 0 | 0.46 | 0.882 |
| TPX2 | DEPDC1 | 0.782 | 0 | 0 | 0.573 | 0.903 |
| TPX2 | ZWINT | 0.771 | 0 | 0 | 0.639 | 0.913 |
| TPX2 | PTTG1 | 0.77 | 0 | 0 | 0.673 | 0.921 |
| TPX2 | NEK2 | 0.818 | 0 | 0 | 0.696 | 0.942 |
| TPX2 | CEP55 | 0.804 | 0 | 0 | 0.727 | 0.944 |
| TPX2 | CDK1 | 0.825 | 0.045 | 0 | 0.736 | 0.952 |
| TPX2 | CENPF | 0.829 | 0 | 0 | 0.746 | 0.954 |
| TPX2 | CCNA2 | 0.86 | 0 | 0 | 0.691 | 0.954 |
| TPX2 | KIF4A | 0.861 | 0 | 0 | 0.764 | 0.965 |
| TPX2 | HMMR | 0.799 | 0.328 | 0 | 0.856 | 0.979 |
| TROAP | CENPM | 0.369 | 0 | 0 | 0.16 | 0.448 |
| TROAP | AURKA | 0.497 | 0 | 0 | 0.165 | 0.563 |
| TROAP | PIMREG | 0.436 | 0 | 0 | 0.359 | 0.623 |
| TROAP | NCAPG | 0.544 | 0 | 0 | 0.348 | 0.69 |
| TROAP | UBE2T | 0.324 | 0 | 0 | 0.164 | 0.411 |
| TROAP | TK1 | 0.332 | 0 | 0 | 0.185 | 0.432 |
| TROAP | SPC25 | 0.331 | 0 | 0 | 0.187 | 0.432 |
| TROAP | SGO1 | 0.412 | 0 | 0 | 0.077 | 0.434 |
| TROAP | DEPDC1B | 0.369 | 0 | 0 | 0.149 | 0.44 |
| TROAP | CDKN3 | 0.47 | 0 | 0 | 0 | 0.47 |
| TROAP | ASF1B | 0.42 | 0 | 0 | 0.137 | 0.478 |
| TROAP | DEPDC1 | 0.433 | 0 | 0 | 0.145 | 0.494 |
| TROAP | SKA3 | 0.404 | 0 | 0 | 0.199 | 0.502 |
| TROAP | RAD54L | 0.478 | 0 | 0 | 0.087 | 0.503 |
| TROAP | NUF2 | 0.375 | 0 | 0 | 0.254 | 0.514 |
| TROAP | CDK1 | 0.49 | 0 | 0 | 0.148 | 0.547 |
| TROAP | HMMR | 0.516 | 0 | 0 | 0.118 | 0.555 |
| TROAP | CENPE | 0.522 | 0 | 0 | 0.146 | 0.575 |
| TROAP | KIF11 | 0.554 | 0 | 0 | 0.136 | 0.598 |
| TROAP | CEP55 | 0.487 | 0 | 0 | 0.274 | 0.612 |
| TROAP | PTTG1 | 0.497 | 0 | 0 | 0.138 | 0.637 |
| TROAP | CENPA | 0.586 | 0 | 0 | 0.203 | 0.656 |
| TROAP | CDC25C | 0.553 | 0 | 0 | 0.265 | 0.657 |
| TROAP | CCNA2 | 0.601 | 0 | 0 | 0.185 | 0.661 |
| TROAP | CCNB2 | 0.564 | 0 | 0 | 0.282 | 0.674 |
| TROAP | TPX2 | 0.572 | 0 | 0 | 0.274 | 0.676 |
| TROAP | NEK2 | 0.478 | 0 | 0 | 0.423 | 0.686 |
| TROAP | CENPF | 0.669 | 0 | 0 | 0.315 | 0.764 |
| TROAP | SPAG5 | 0.732 | 0 | 0 | 0.332 | 0.813 |
| TROAP | KIF4A | 0.668 | 0 | 0 | 0.527 | 0.836 |
| TROAP | KIF18B | 0.86 | 0 | 0 | 0.356 | 0.906 |
| UBE2T | CDC6 | 0.346 | 0.077 | 0 | 0.327 | 0.558 |
| UBE2T | CENPM | 0.409 | 0 | 0 | 0.108 | 0.451 |
| UBE2T | AURKA | 0.461 | 0.067 | 0 | 0.358 | 0.649 |
| UBE2T | GMNN | 0.626 | 0 | 0 | 0.212 | 0.693 |
| UBE2T | PIMREG | 0.341 | 0 | 0 | 0.149 | 0.415 |
| UBE2T | NCAPG | 0.503 | 0.059 | 0 | 0.162 | 0.574 |
| UBE2T | TROAP | 0.324 | 0 | 0 | 0.164 | 0.411 |
| UBE2T | KIF11 | 0.438 | 0 | 0 | 0.179 | 0.519 |
| UBE2T | ASF1B | 0.363 | 0.095 | 0 | 0.189 | 0.492 |
| UBE2T | DEPDC1B | 0.466 | 0.292 | 0 | 0.286 | 0.706 |
| UBE2T | CENPE | 0.344 | 0.045 | 0 | 0.123 | 0.403 |
| UBE2T | NUF2 | 0.801 | 0 | 0 | 0.521 | 0.901 |
| UBE2T | SPC25 | 0.478 | 0.069 | 0 | 0.134 | 0.542 |
| UBE2T | CCNB2 | 0.487 | 0.072 | 0 | 0.346 | 0.662 |
| UBE2T | MAD2L1 | 0.783 | 0.059 | 0 | 0.273 | 0.838 |
| UBE2T | TPX2 | 0.559 | 0.042 | 0 | 0.562 | 0.799 |
| UBE2T | RMI2 | 0.462 | 0 | 0 | 0.455 | 0.694 |
| UBE2T | CKS1B | 0.688 | 0.045 | 0 | 0.127 | 0.717 |
| UBE2T | SKA3 | 0.461 | 0 | 0 | 0.169 | 0.533 |
| UBE2T | CDC25C | 0.343 | 0.048 | 0 | 0.164 | 0.432 |
| UBE2T | SPAG5 | 0.613 | 0.08 | 0 | 0.05 | 0.632 |
| UBE2T | CDKN3 | 0.78 | 0.047 | 0 | 0.105 | 0.796 |
| UBE2T | CENPA | 0.362 | 0.116 | 0 | 0.159 | 0.484 |
| UBE2T | CENPF | 0.788 | 0 | 0 | 0.333 | 0.853 |
| UBE2T | NEK2 | 0.438 | 0.093 | 0 | 0.246 | 0.582 |
| UBE2T | CEP55 | 0.465 | 0 | 0 | 0.585 | 0.768 |
| UBE2T | RAD54L | 0.342 | 0.099 | 0 | 0.331 | 0.569 |
| UBE2T | ZWINT | 0.63 | 0 | 0 | 0.3 | 0.73 |
| UBE2T | KIF4A | 0.387 | 0.07 | 0 | 0.51 | 0.696 |
| UBE2T | CKS2 | 0.537 | 0.045 | 0 | 0.188 | 0.61 |
| UBE2T | BRCA2 | 0.149 | 0 | 0 | 0.597 | 0.643 |
| UBE2T | ECT2 | 0.285 | 0 | 0 | 0.383 | 0.54 |
| UBE2T | EME1 | 0.233 | 0.047 | 0.5 | 0.309 | 0.713 |
| UBE2T | HMMR | 0.495 | 0.08 | 0 | 0.168 | 0.579 |
| UBE2T | PTTG1 | 0.786 | 0 | 0 | 0.348 | 0.854 |
| UBE2T | CDK1 | 0.783 | 0.091 | 0 | 0.275 | 0.844 |
| UBE2T | CENPK | 0.371 | 0 | 0 | 0.113 | 0.419 |
| UBE2T | MTFR2 | 0.293 | 0.064 | 0 | 0.204 | 0.427 |
| UBE2T | DEPDC1 | 0.373 | 0 | 0 | 0.182 | 0.466 |
| UBE2T | KPNA2 | 0.385 | 0 | 0 | 0.066 | 0.402 |
| UBE2T | TK1 | 0.478 | 0 | 0 | 0.459 | 0.705 |
| UBE2T | CCNA2 | 0.474 | 0.101 | 0 | 0.294 | 0.637 |
| UBE2T | RAD51 | 0.436 | 0.114 | 0 | 0.5 | 0.728 |
| WDR76 | GMNN | 0.098 | 0 | 0 | 0.365 | 0.402 |
| WDR76 | KIF11 | 0.32 | 0.292 | 0 | 0.21 | 0.586 |
| ZWINT | CDC6 | 0.63 | 0 | 0 | 0.248 | 0.71 |
| ZWINT | CENPM | 0.655 | 0.169 | 0 | 0.402 | 0.814 |
| ZWINT | AURKA | 0.431 | 0 | 0 | 0.642 | 0.787 |
| ZWINT | GMNN | 0.5 | 0 | 0 | 0 | 0.5 |
| ZWINT | KNSTRN | 0.27 | 0 | 0 | 0.224 | 0.409 |
| ZWINT | PIMREG | 0.334 | 0 | 0 | 0.213 | 0.453 |
| ZWINT | NCAPG | 0.657 | 0 | 0 | 0.587 | 0.852 |
| ZWINT | KIF11 | 0.794 | 0 | 0 | 0.665 | 0.928 |
| ZWINT | ASF1B | 0.369 | 0 | 0 | 0.22 | 0.487 |
| ZWINT | SGO1 | 0.127 | 0 | 0 | 0.363 | 0.42 |
| ZWINT | DEPDC1B | 0.45 | 0 | 0 | 0.175 | 0.527 |
| ZWINT | CENPE | 0.488 | 0.087 | 0 | 0.76 | 0.878 |
| ZWINT | NUF2 | 0.641 | 0.887 | 0.54 | 0.606 | 0.991 |
| ZWINT | SPC25 | 0.665 | 0.88 | 0.54 | 0.281 | 0.984 |
| ZWINT | CCNB2 | 0.539 | 0 | 0.4 | 0.735 | 0.92 |
| ZWINT | MAD2L1 | 0.542 | 0.191 | 0.5 | 0.523 | 0.899 |
| ZWINT | TPX2 | 0.771 | 0 | 0 | 0.639 | 0.913 |
| ZWINT | CKS1B | 0.342 | 0 | 0 | 0.18 | 0.437 |
| ZWINT | SKA3 | 0.495 | 0.172 | 0 | 0.398 | 0.726 |
| ZWINT | CDC25C | 0.382 | 0 | 0 | 0.248 | 0.515 |
| ZWINT | SPAG5 | 0.554 | 0 | 0 | 0.282 | 0.666 |
| ZWINT | NCAPD2 | 0.242 | 0 | 0 | 0.291 | 0.439 |
| ZWINT | CDKN3 | 0.639 | 0 | 0 | 0.138 | 0.676 |
| ZWINT | KNL1 | 0.259 | 0.913 | 0.54 | 0.986 | 0.999 |
| ZWINT | CENPA | 0.5 | 0 | 0 | 0.825 | 0.908 |
| ZWINT | CENPF | 0.638 | 0 | 0 | 0.877 | 0.953 |
| ZWINT | NEK2 | 0.39 | 0 | 0 | 0.662 | 0.785 |
| ZWINT | CEP55 | 0.781 | 0 | 0 | 0.418 | 0.867 |
| ZWINT | RAD54L | 0.393 | 0 | 0 | 0.261 | 0.532 |
| ZWINT | CENPI | 0.197 | 0 | 0 | 0.654 | 0.71 |
| ZWINT | KIF18B | 0.358 | 0.065 | 0 | 0.1 | 0.413 |
| ZWINT | KPNA2 | 0.358 | 0 | 0 | 0.246 | 0.495 |
| ZWINT | DEPDC1 | 0.425 | 0 | 0 | 0.202 | 0.521 |
| ZWINT | CKS2 | 0.396 | 0 | 0 | 0.394 | 0.618 |
| ZWINT | CENPK | 0.305 | 0.171 | 0 | 0.436 | 0.646 |
| ZWINT | ECT2 | 0.315 | 0 | 0 | 0.545 | 0.675 |
| ZWINT | PTTG1 | 0.537 | 0 | 0 | 0.431 | 0.725 |
| ZWINT | UBE2T | 0.63 | 0 | 0 | 0.3 | 0.73 |
| ZWINT | RAD51 | 0.535 | 0 | 0 | 0.456 | 0.736 |
| ZWINT | KIF4A | 0.745 | 0 | 0 | 0.43 | 0.848 |
| ZWINT | HMMR | 0.772 | 0 | 0 | 0.543 | 0.891 |
| ZWINT | TK1 | 0.776 | 0 | 0 | 0.567 | 0.898 |
| ZWINT | CCNA2 | 0.774 | 0 | 0 | 0.58 | 0.901 |
| ZWINT | CDK1 | 0.79 | 0 | 0.4 | 0.638 | 0.95 |
